# Costs of multimorbidity: a systematic review and meta-analyses

**DOI:** 10.1186/s12916-022-02427-9

**Published:** 2022-07-19

**Authors:** Phuong Bich Tran, Joseph Kazibwe, Georgios F. Nikolaidis, Ismo Linnosmaa, Mieke Rijken, Josefien van Olmen

**Affiliations:** 1 Department of Family Medicine and Population Health, Faculty of Medicine and Health Sciences, University of Antwerp, Antwerp, Belgium; 2 Department of Clinical Sciences, Faculty of Medicine, Lund University, Lund, Sweden; 3 Department of Global Health, Faculty of Public Health and Policy, London School of Hygiene and Tropical Medicine, London, UK; 4 IQVIA Ltd., 37 North Wharf Road, London, W2 1AF UK; 5 Department of Health and Social Management, University of Eastern Finland, Kuopio, Finland; 6 Netherlands Institute for Health Services Research, Utrecht, The Netherlands

**Keywords:** Multimorbidity, Chronic diseases, Cost, Economic burden, Health system

## Abstract

**Background:**

Multimorbidity is a rising global phenomenon, placing strains on countries’ population health and finances. This systematic review provides insight into the costs of multimorbidity through addressing the following primary and secondary research questions: What evidence exists on the costs of multimorbidity? How do costs of specific disease combinations vary across countries? How do multimorbidity costs vary across disease combinations? What “cost ingredients” are most commonly included in these multimorbidity studies?

**Methods:**

We conducted a systematic review (PROSPERO: CRD42020204871) of studies published from January 2010 to January 2022, which reported on costs associated with combinations of at least two specified conditions. Systematic string-based searches were conducted in MEDLINE, The Cochrane Library, SCOPUS, Global Health, Web of Science, and Business Source Complete. We explored the association between costs of multimorbidity and country Gross Domestic Product (GDP) per capita using a linear mixed model with random intercept. Annual mean direct medical costs per capita were pooled in fixed-effects meta-analyses for each of the frequently reported dyads. Costs are reported in 2021 International Dollars (I$).

**Results:**

Fifty-nine studies were included in the review, the majority of which were from high-income countries, particularly the United States. (1) Reported annual costs of multimorbidity per person ranged from I$800 to I$150,000, depending on disease combination, country, cost ingredients, and other study characteristics. (2) Our results further demonstrated that increased country GDP per capita was associated with higher costs of multimorbidity. (3) Meta-analyses of 15 studies showed that on average, dyads which featured Hypertension were among the least expensive to manage, with the most expensive dyads being Respiratory and Mental Health condition (I$36,840), Diabetes and Heart/vascular condition (I$37,090), and Cancer and Mental Health condition in the first year after cancer diagnosis (I$85,820). (4) Most studies reported only direct medical costs, such as costs of hospitalization, outpatient care, emergency care, and drugs.

**Conclusions:**

Multimorbidity imposes a large economic burden on both the health system and society, most notably for patients with cancer and mental health condition in the first year after cancer diagnosis. Whether the cost of a disease combination is more or less than the additive costs of the component diseases needs to be further explored. Multimorbidity costing studies typically consider only a limited number of disease combinations, and few have been conducted in low- and middle-income countries and Europe. Rigorous and standardized methods of data collection and costing for multimorbidity should be developed to provide more comprehensive and comparable evidence for the costs of multimorbidity.

**Supplementary Information:**

The online version contains supplementary material available at 10.1186/s12916-022-02427-9.

## Background

Across the world, the prevalence of multimorbidity is increasing, especially among older populations. Studies reporting the prevalence of multimorbidity have given rates ranging from 32% of patients attending general practices in the Netherlands [1] to 99% of patients attending ambulatory care in Canada [2]. Across Europe, many countries have seen 40–60% of those aged 50 years or older living with multimorbidity [3–5]. In low- and middle-income countries (LMICs), the prevalence of multimorbidity ranges from 3.2 to 90.5% depending on the age group [6]. While multimorbidity risk increases with age, in some LMICs, more cases of multimorbidity can be found among those under the age of 65, due to expansive population pyramids [7] and a higher prevalence of risk factors [8, 9]. Multimorbidity is thus a major public health problem to be urgently addressed.

Beyond the prevalence of the individual “combiners” — conditions found in a multimorbity combination — the prevalence of a disease combination is typically linked to both the age structure of the population [10] and behavioral risk factors [11]. Common combiners — including diabetes, hypertension, osteoarthritis, and mental health conditions such as depression — usually appear across different disease combinations [12–14]. Epidemiological research on the distribution and underlying determinants of multimorbidity is still evolving [7], yet its impact on healthcare systems and societies is becoming increasingly clear.

Treating multimorbidity is generally more complex than treating single diseases, thereby increasing the demand for healthcare resources. Such treatment often requires additional customization for the patient and often lasts longer [8, 15, 16]. Many multimorbid patients also experience functional limitations and are required to spend more time visiting healthcare providers. It is common for these issues to interfere with a patient’s work, which in turn contributes to societal costs [17]. Consequently, as the prevalence of multimorbidity continues to rise, countries face not only the challenges of providing quality multimorbidity care, but must also make preparations to shoulder the economic burden it brings about [18, 19].

While there is much research exploring costs associated with single chronic conditions, studies into multimorbidity costs are limited in scope and number. Among these studies, most have tended to focus on either the number of conditions or the severity of multimorbidity, the latter typically estimated using measures such as the *Charlson Comorbidity Index* [20]. There have been two systematic reviews on the costs of multimorbidity per-disease-count, with inclusion periods of 1996–2013 and 1992–2010 [21, 22]. While both studies reported that patients with multimorbidity incurred higher costs than those without, neither explored the costs of specific disease combinations.

In line with global health priorities, we have reviewed the evidence on the costs of multimorbidity to address the question, “Which disease clusters result in the greatest costs?” [7]. In conducting the review, we further addressed the following primary research question (#1) and secondary research questions (#2–4):

1. What evidence exists on the costs of multimorbidity?
2. How do costs of specific disease combinations vary across countries?
3. How do multimorbidity costs vary across disease combinations?
4. What “cost ingredients” are most commonly included in these multimorbidity studies?

The findings of this review are important to disentangle our understanding of the economic burden of multimorbidity, which in turn will inform both the implementation of health interventions and the optimization of healthcare delivery.

## Methods

We firstly conducted a systematic review applying a narrative synthesis of included studies, following the Preferred Reporting Items for Systematic Reviews and Meta-Analyses (PRISMA) guidelines [23]. Following this, we conducted meta-analyses including those studies which had provided comparable information. This review is registered with PROSPERO (*ref CRD42020204871*).

### Databases and search terms

The initial search was conducted on 9 October 2020, with a follow-up search on 4 January 2022. Databases searched were *Web of Science*, *Global Health*, *MEDLINE*, *SCOPUS*, *The Cochrane Library*, and *Business Source Complete*. A snowballing technique was adopted to identify further articles from within the reference lists of eligible studies. The database-specific search strings are detailed in Additional file 1 — Search strings.

### Study selection

**Fig. 1:**
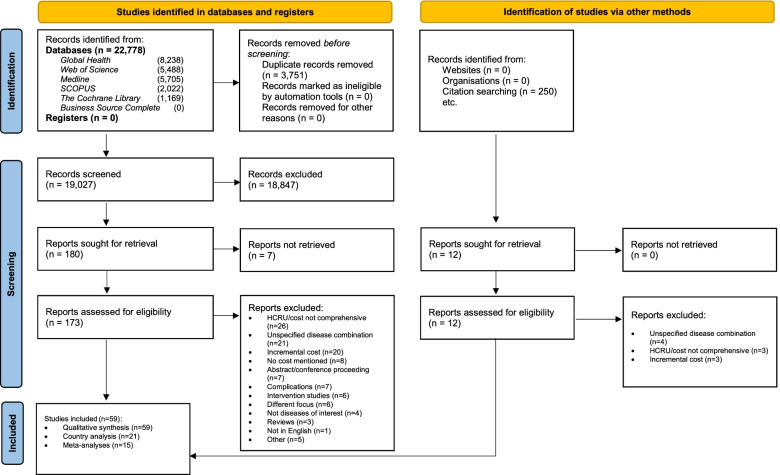
Flow diagram of the study selection process

The search included all quantitative studies described in full-text papers, published in English between 1 January 2010 and 4 January 2022. Papers were eligible for inclusion if they reported the costs associated with the co-existence of at least two chronic conditions. We excluded studies not specifying combinations of chronic conditions and those which had estimated incremental costs only (for full list of exclusion criteria, see Fig. 1).

Following the removal of duplicates, articles were screened based upon their titles and abstracts, using *Rayyan QCRI* software [24] (phase 1). Articles were subsequently transferred to *Endnote X9* for full-text screening and reference management (phase 2). Both screening phases were conducted by two authors (PT and JK) using a checklist (Additional file 2 — Screening checklists), and conflicts resolved with the senior author’s input (JVO).

### Quality assessment

The quality of studies was assessed by two authors independently (PT and JK) using the *Newcastle–Ottawa Quality Assessment Scale (NOS)* [25]. In the case of discrepancies, consensus was reached with input from other authors.

### Data extraction and classification

A REDCap form, which had previously been piloted and refined, was used to facilitate the data extraction process. REDCap was chosen due to its level of data encryption, user-friendliness, and its capabilities for customization in data collection [26]. The following data were extracted: study characteristics, sample size, disease characteristics, care setting, and costs. The data cleaning process is presented in Additional file 3 — Data cleaning flow chart.

### Cost parameter

This study’s primary outcome measure is the *average annual cost per patient per disease combination*, comprising of direct medical costs, direct non-medical costs, and indirect costs. Direct medical cost is the cost of a defined health service or intervention and all follow-up costs for medication and medical equipment (diagnostics, hospitalization, outpatient, emergency, drugs, and equipment) [27]. Direct non-medical cost is the cost incurred in the process of seeking and after receiving health services that are not involved in the direct purchasing of medical products or services (transportation/travel costs, food, accommodation, and additional paid caregiver time) [27]. Indirect costs are those incurred as a result of losses from the disease(s) or disease management (time loss, wage loss, interest from debts/loans) [27]. Other terminologies are explained in Additional file 4 — Definition of terminologies. All costs are reported in 2021 International Dollars (denoted by I$), which is a hypothetical currency with the same purchasing power in every country, using the US as a reference [28]. First, the reported cost was inflated to 2021 local currency unit [29]; then, it was converted to International Dollar using 2021 *Purchasing Power Parities (PPP)* [30]*.*

The costing perspective is important in a costing study as it determines which costs are included (direct medical/non-medical, indirect costs), the source of data, and the scope of the study. The costing perspective may reflect a patient (often out-of-pocket), an organization (provider), a health system (public or private), or all of society [31]. This review includes all costing perspectives, but mainly reports on the health system perspective as it is accounted for in the majority of studies. The health system perspective entails formal direct medical costs paid for by third-party payers or by patients [32].

### Analysis and presentation of results

To address research question 1, we tabulated all combinations of conditions and their costs as described in the studies. For one study that reported costs at baseline and follow-ups, costs were pooled to arrive at an average estimate [33].

Related conditions were grouped together. For example, *Type 1* and *Type 2 diabetes* were classified as “Diabetes”. *Mental disorder*, *anxiety disorder*, and *depression* were grouped as “Mental Health conditions”. *Asthma*, *chronic obstructive pulmonary disease (COPD)*, and *tuberculosis (TB)* were combined under the category “Respiratory diseases”. *Cardiovascular disease*, *coronary atherosclerosis*, *congestive heart failure*, *coronary heart disease*, *atrial fibrillation*, *coronary artery disease*, *peripheral artery disease*, *myocardial infarction*, *heart disease/failure*, *cerebrovascular disease*, *conduction disorder or cardiac dysrhythmia, valvular disease, peripheral vascular disorders*, and *pulmonary circulation disorders* were classified as “Heart/vascular conditions”. “Cancers” included *thyroid*, *stomach*, *breast*, *uterus*, *kidney*, *colon and rectum*, *esophagus*, *pancreas*, *head and neck*, *other gastrointestinal*, *liver*, *ovarian*, *multiple myeloma*, and *any malignancy/tumor*. Alternate groupings would have been possible, and those following a more treatment-focused perspective may have led to variation in results. However, we resorted to this approach in order to reduce the number of combinations and condense information for ease of interpretations. Grouping these conditions at an organ system level makes sense from a health system/organizational perspective considering that they show similarities related to medical specialties. For example, *lung cancer* is grouped together with other cancer sites and not with the respiratory diseases, considering that cancers are treated by oncologists and most other (severe) lung or respiratory conditions are treated by pulmonologists. On the other hand, we categorized *hypertension* separately from the heart/vascular group as it is the leading metabolic risk factor globally [34]. Moreover, most studies also report hypertension separately; therefore, following this approach allowed for cross-comparison between studies. Lastly, we did not include stroke in the heart/vascular group as it is considered a chronic disease with acute exacerbations, in which the cascade of care is important in contextualizing the costs across the patient care pathway; therefore, the cost of stroke cannot be interpreted together with other heart/vascular conditions [35].

This resulted in six main disease categories: (1) Diabetes, (2) Heart/vascular conditions, (3) Respiratory diseases, (4) Cancers, (5) Mental Health conditions, and (6) Hypertension. The first four and mental health conditions are classified by the World Health Organization as major noncommunicable diseases (NCDs) [36].

Research question 2 aimed to contextualize the variability in costs using country GDP per capita in 2020 (latest available data) [37, 38]. For this analysis, we included only the most frequently reported dyads. We used the same study eligibility criteria as for the meta-analyses (see below), with several conditions relaxed. The criteria that studies must have had the same design, and reported measures of distribution and all-cause costs were relaxed, as we could control for these factors in the model. First, we ran a linear model with *annual mean direct medical costs per capita* as the dependent variable and *GDP per capita* as the independent variable taking on fixed effects. Subsequently, we incorporated different study characteristics as random effects. Potential study characteristics that may affect costs are study, study design, data source, and country. We performed log10 transformations on costs and GDP to normalize the distribution and to stabilize the variation within groups. After testing different models and observing variance, *p*-value and Akaike Information Criterion (AIC), the best fit model consisted of *GDP* as fixed effects and *study* and *data source* as random effects. The analysis was performed in RStudio version 2021.09.2 [39].

To compare the costs of disease combinations and to identify those that resulted in high costs (research question 3), meta-analyses were conducted for the most frequently reported dyads. Studies were categorized to ensure similarities within each sub-group meta-analysis. The criteria for homogeneity were (1) same cost perspective and study design, (2) reporting annual mean direct medical cost, (3) reporting measures of distribution, (4) comparability of cost ingredients determined by recurring ingredients (hospitalization, outpatient care, emergency care, drugs), (5) studies assessed together having either all specified all-cause healthcare cost or not specified at all, and (6) studies that only assessed costs specific to the disease(s) of interest were not included. For studies that reported more than one estimate for the same dyad, these estimates were pooled before being entered into the meta-analysis — provided that the mean cost, its standard error, and the sample size corresponding to each were provided [40–42]. Where appropriate, costs per month or per 6 months were multiplied by 12 or 2, respectively, to arrive at the estimates for 12 months [40, 42, 43].

Mean cost data were meta-analyzed assuming a normal likelihood for study-specific mean costs. Despite the non-normal nature of healthcare costs [44], the distribution of sample mean costs will approximate a normal distribution as the number of studies increases due to the Central Limit Theorem. Given the low number of studies that were available for some disease combinations, a fixed-effects model was prioritized on practical grounds, acknowledging the strength of the imposed assumption (i.e., a common underlying true cost across all studies). Random-effects models were also attempted, noting that the low number of studies may lead to convergence issues and unrealistic estimates of the between-study heterogeneity [45]. The extent of heterogeneity was estimated and presented by the means of *I*^2^ [46]. All synthesis models were implemented in OpenBUGS version 3.2.3 [47] using three Markov Chain Monte Carlo chains with different starting values. Estimates were obtained from 70,000 iterations (including 20,000 burn-in). Convergence was checked using the Gelman-Rubin diagnostic, specifically with the multivariate potential scale reduction factor [32], and visually by assessing the history, chains, and autocorrelation. Vague priors were used for all parameters.

Finally, cost ingredients such as *diagnostics*, *outpatient care*, *hospitalization*, *emergency care*, and *medications* were tabulated and descriptively analyzed.

## Results

**Fig. 2:**
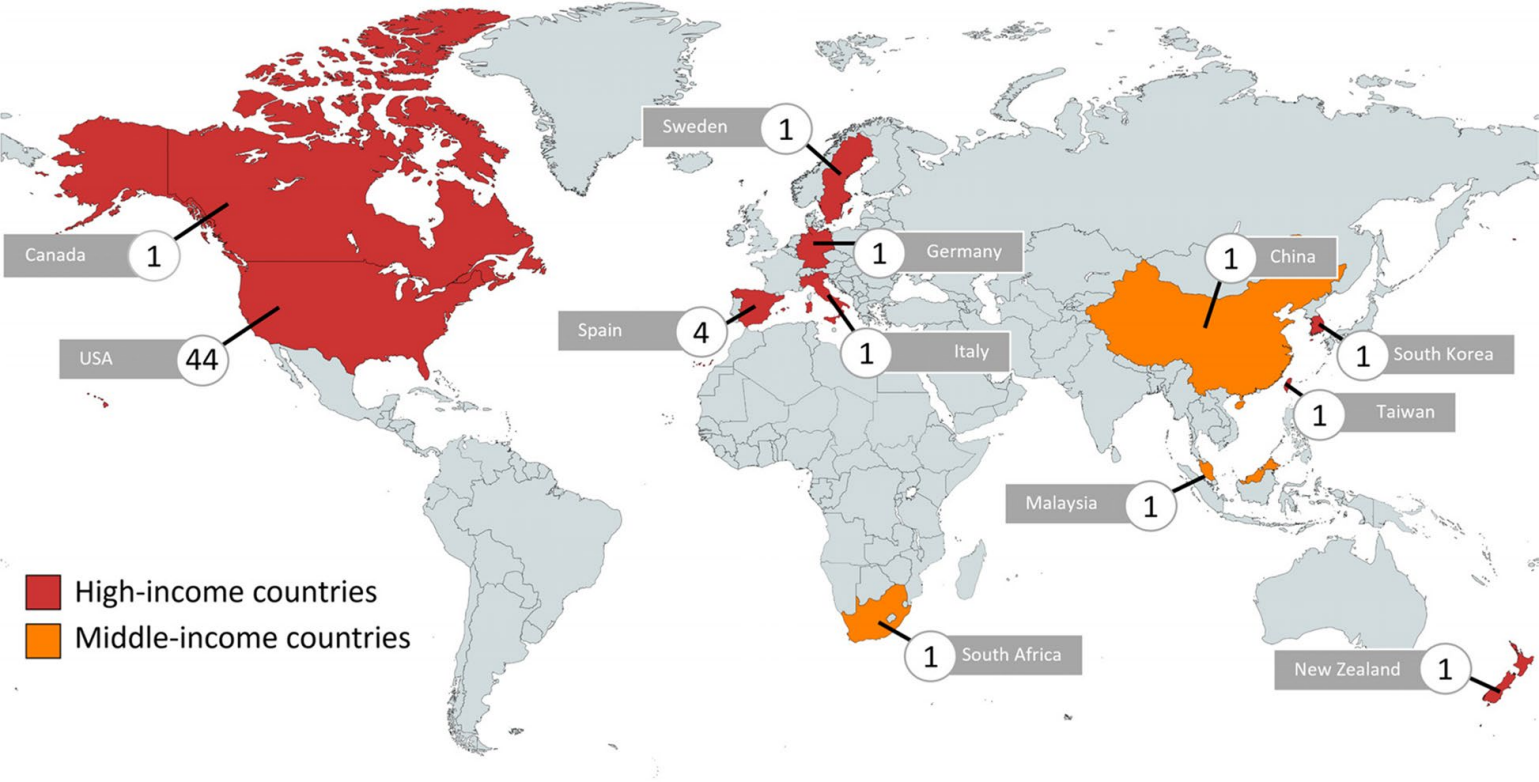
Geographic locations of study settings and number of studies in each setting. Legend: We mapped out countries where the studies were conducted and the respective number of studies identified for each country. The color codes show the different levels of country income, according to the World Bank classification in 2021

Our search identified 22,778 publications from six databases and a further 12 studies from reference searches. Following deduplication, 19,027 titles/abstracts were screened with 173 eligible for full-text review. In all, 59 studies were selected in this review (Fig. 2).

### Study characteristics

Charting of the study characteristics is further presented in Additional file 5. Twelve countries were represented in the 59 studies, mostly high-income countries (95%), primarily the United States (US) (*n* = 44) and Spain (*n* = 4) and one multi-country study (Table 1).

**Table 1:** Summary characteristics of studies (*N* = 59)

Study characteristic	Articles ***n*** (%)
**Type of study (*N*** **= 59)**	
Cross-sectional	32 (54%)
Cohort/longitudinal	26 (44%)
Case-control	1 (2%)
**World Bank classification (*n*** **= 59)**	
High-income country	56 (95%)
Middle-income country	3 (5%)
Low-income country	0 (0)
**Source of data (*n*** = 59)*	
Insurance claim database	37 (63%)
Hospital/medical database	19 (32%)
Patient survey	20 (34%)
Linked database	20 (34%)
Other	6 (10%)
**Type of costs included (*n*** = 59)*	
Direct medical costs	58 (98%)
Direct non-medical costs	2 (3%)
Indirect costs	7 (12%)
**Costing perspective (*n*** = 59)*	
Public	48 (81%)
Household/patient	12 (20%)
Societal	8 (14%)
Provider	1 (2%)
Number of disease combination sets from all studies* (*n* **= 325)**	
Included in narrative synthesis (research questions 1 and 4)	229 (70%)
Included in country comparison (research question 2)	41 (13%)
Included in meta-analyses (research question 3)	34 (10%)
**Number of conditions in each disease combination set (*n*** **= 325)**	
Two	282 (87%)
More than two	43 (13%)
Frequently appeared in disease combination sets* (*n* **= 325)**	
Diabetes	96 (30%)
Heart/vascular conditions	93 (29%)
Mental health conditions	67 (21%)
Hypertension	59 (18%)
Respiratory conditions	52 (16%)
Cancers	38 (12%)
**Quality score**	**Mean**
Cross-sectional studies (*n* = 32)	7.8 out of 10
Cohort studies (*n* = 26)	7.9 out of 9
Case-control studies (*n* = 1)	7.0 out of 9

*Studies can belong to more than one category, meaning individual percentages at times do not sum to 100%

Over half of studies were cross-sectional (54%), followed by longitudinal/cohort (44%). Across the 59 studies, the primary source of data was insurance claim databases (63%). Patient survey data were used in 20 studies (34%), so were medical databases. A third of all studies linked data across multiple databases. The “other” category comprises sub-databases, for example, the cancer registry, the national inpatient sample, and the drug prescription registry [48–50]. Most articles reported direct medical costs (98%), with few reporting direct non-medical (3%) [51, 52] or indirect costs (12%) [51–57].

Quality assessment scores tended to be higher among cohort/longitudinal studies, and those which had used linked databases. All studies achieved the inclusion threshold of 5 points. Patient surveys had been used in a third of all studies. As acknowledged to contain a risk of bias (recall, language, time), most of the included studies which used patient survey (i.e., Medical Expenditure Panel Survey (MEPS)) [41, 58–63] had cross-checked collected data with administrative data [64]. Several ambiguities were observed among studies. Those using data from insurance claims databases often did not specify whether reported estimates included copayments or deductibles [65–67]. Many studies also did not specify whether the reported estimates were of adjusted or unadjusted costs, all-cause healthcare costs or disease-specific costs [60–62]. A small number of studies did not clearly specify the year of currency, the timeframe of reported costs (per patient per month/year/two years), the sample size related to the estimate, or the measure of distribution [58, 61, 65, 68]. Finally, challenges were present in making comparisons of cost ingredients across studies, with some having broader categorizations (inpatient, outpatient, emergency care, pharmacy) and some having smaller categories (diagnostics, physician, specialist) or a mix of both [43, 65, 69].

#### Focus of multimorbidity

While five studies had explored a wide range of disease combinations from the population or sub-population level [48, 55, 60, 66, 70], the majority of studies focused on one [33, 41, 43, 52, 53, 56, 59, 65, 71–88] or several disease combinations relating to an index disease of interest [40, 42, 49–51, 54, 57, 58, 60–62, 67–69, 73, 89–103].

Of the 325 disease combinations featured across all studies, 87% were combinations of two co-existing conditions (dyads), while 13% were combinations involving three (triads) or more co-existing conditions. A large proportion of these combinations involved diabetes (30% of combinations) and heart/vascular conditions (29%). Other diseases which featured prominently in disease combinations were mental health conditions (21%), hypertension (18%), respiratory conditions (16%), and cancers (12%).

To condense information, we selected 229 sets of disease combinations, each of which involved at least one of the above frequently reported diseases to include in the narrative synthesis. The remaining sets (highlighted in Additional file 6 — Cost conversion table) are those which either did not include one of these frequently reported diseases, those that included comorbidities not originally in our search strings, or those which had not met our criteria for reporting mean direct cost per capita. We focused mostly on dyads. Of the 229 sets of disease combinations, 41 were selected for the country comparison and 34 for the meta-analyses. The following 11 groups were the most frequently reported combinations:

**Table:** 

1	Cancer + Mental health condition (first year after cancer diagnosis)
2	Diabetes + Heart/vascular condition
3	Diabetes + Hypertension
4	Diabetes + Kidney disease
5	Diabetes + Mental health condition
6	Hypertension + Heart/vascular condition
7	Hypertension + Kidney disease
8	Hypertension + Musculoskeletal disorder
9	Hypertension + Respiratory condition
10	Respiratory condition + Heart/vascular condition
11	Respiratory condition + Mental health condition

### Research question 1: Evidence on the costs of multimorbidity

The variation in reporting in the underlying studies presented challenges in comparing costs between different disease combinations in a homogenous way. Across the 229 sets of disease combinations, costs per year ranged from I$827 for a TB-diabetes patient in Malaysia [71] to I$147,784 for a patient with HIV and pulmonary circulation disorders in the US (Additional file 7: Table S2) [99]. Several studies reported high costs for multimorbidity in the last 6 months of life or the 6 months immediately after diagnosis. For example, cost in the last 6 months of life for a patient in the US with heart failure and diabetes is I$51,145 [72], considerably higher than that of similar patients earlier in life [33, 60, 90]. Reported costs are also high when they incorporate direct non-medical and indirect costs. In the case of patients diagnosed with stroke and diabetes in Spain, the annual cost reported per patient, which included indirect costs, was I$52,606 [51]. Of this total, direct medical costs account for merely a quarter.

Table S2: Available evidence on the costs of multimorbidity (Additional file 7)

### Research question 2: The variation in costs across countries

The list of included studies in this section is in Additional file 8 — Checklist and data used for the country comparison. Studies selected for this analysis were of the same cost perspective, reported annual mean direct medical cost, and had similar cost ingredients determined by recurring ingredients (hospitalization, outpatient care, emergency care, drugs). Where appropriate, costs per month and per 24 months were multiplied by 12 and divided by 2, respectively, to arrive at the annual estimates [40, 43, 98]. Two studies reported costs from a societal perspective; hence, we removed the indirect cost from total costs for the purpose of this analysis [53, 57].

**Fig. 3:**
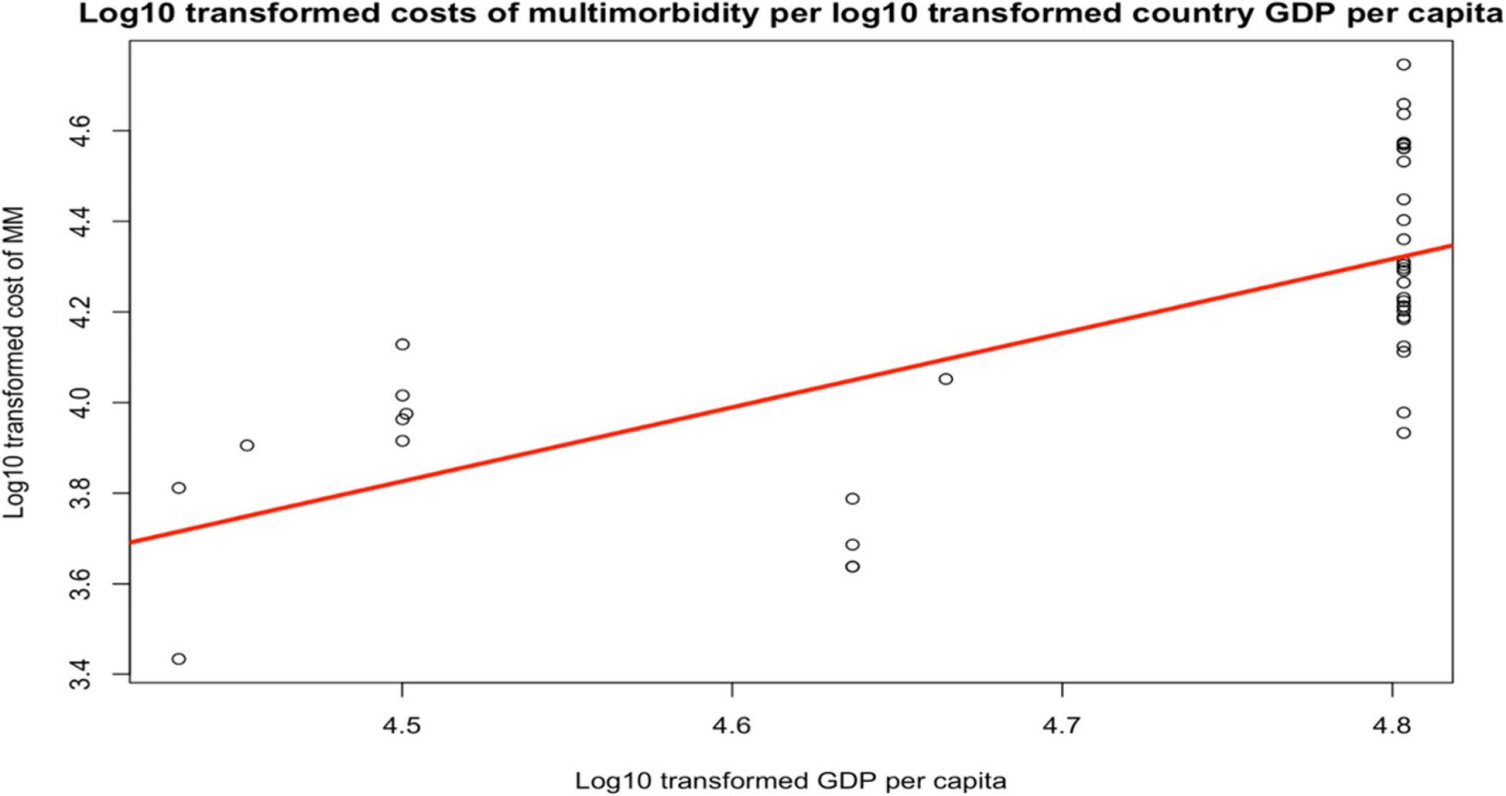
Log10 transformed costs of multimorbidity per log10 transformed country GDP per capita. Legend: Log10 transformed cost per capita of multimorbidity was plotted against log10 transformed country GDP per capita using a fixed-effects model. The red line depicts the relationship

First, log10 transformed cost per capita of multimorbidity was plotted against log10 transformed country GDP per capita using a fixed-effects model (Fig. 3). In the fixed-effects model, country GDP per capita accounts for under half of the variation in cost estimates (*R*^2^ = 0.47; *p* < 0.001). Every International Dollar increase in GDP on the log10 scale is associated with an expected increase of 1.64 International Dollars in the cost of multimorbidity on the log10 scale (*p* < 0.001).

Next, we used a linear mixed model with random intercept to adjust for covariates (Table 2). Country GDP per capita accounts for a high proportion of the variation in cost estimates (*R*^2^ = 0.94; *p* < 0.001). Every International Dollar increase in GDP on the log10 scale is associated with an expected increase of 1.91 International Dollars in the cost of multimorbidity on the log10 scale. In addition, given that the observations derived from different studies, *study* and *data source* accounted for 40% and 47% of the remaining variation in costs, respectively.

**Table 2:** Relationship between direct costs and GDP, adjusted model

**Predictor**	**Estimate**	**Standard error**
Intercept	−4.86*	1.26
Log10 GDP	1.91*	0.27
**Random effects**	**Variance**	
Study	0.02029	
Data source	0.02365	
Residual	0.00684	
AIC	−31.87	
*R-squared adjusted*	0.94	
*N*	41	

Legend: We ran a linear mixed model with various study characteristics as random effects to assess the relationship between direct costs of multimorbidity and country GDP. *p < 0.001

### Research question 3: Meta-analyses of the costs of most frequently reported disease combinations and their variation

The 15 studies in the meta-analyses were selected using stringent homogeneity criteria (checklist and study details in Additional file 9 — Homogeneity checklist and data used for the meta-analyses). This resulted in a small number of data points in each sub-analysis. Random-effects models did not converge for disease combinations with a low number of studies and were not pursued further. Therefore, only results of fixed-effects models are presented for consistency. All meta-analyses resulted in *I*^2^ values above 95%. In the absence of other analyses utilizing a richer and more homogenous evidence base, these estimates may be the most informative to date.

Of the 11 most frequently reported dyads, patients with Cancer + Mental Health condition within the first year of cancer diagnosis had the highest average annual direct medical costs (I$85,820) and greatest intra-group variability (Table 3). This is the only care-cascade-specific sub-group among the 11 most frequently reported dyads. The cost of this dyad is two to six times higher than that of other dyads.

**Table 3:** Results of meta-analyses

Dyad	Pooled mean cost (I$)	95%CI
Hypertension + Musculoskeletal disorder	13,270	(12,960–13,580)
Hypertension + Diabetes	14,300	(13,940–14,660)
Hypertension + Respiratory condition	17,180	(16,530–17,830)
Hypertension + Kidney disease	17,740	(17,060–18,420)
Hypertension + Heart/vascular condition	17,880	(17,520–18,250)
Diabetes + Mental health condition	20,750	(19,830–21,660)
Diabetes + Kidney disease	32,410	(32,370–32,440)
Respiratory + Heart/vascular condition	35,070	(34,890–35,260)
Respiratory + Mental health condition	36,840	(36,440–37,250)
Diabetes + Heart/vascular condition	37,090	(35,870–38,310)
Cancer + Mental health condition *(first year after cancer diagnosis)*	85,820	(81,930–89,690)

Legend: Mean cost data were meta-analyzed using a fixed-effects model assuming a normal likelihood for study-specific mean costs. All meta-analyses resulted in *I*^2^ values above 95%. Costs are presented in 2021 International Dollars

Patients with Hypertension + Musculoskeletal disorder incurred the lowest average annual direct medical costs (I$13,270), followed by patients with hypertension comorbid with diabetes (I$14,300), respiratory condition (I$17,180), kidney disease (I$17,740), and heart/vascular condition (I$17,880). In general, dyads involving hypertension were among the least expensive to manage.

Kidney disease when comorbid with hypertension resulted in a lower mean cost than when comorbid with diabetes [I$17,740 (95% CI: 17,060–18,420) *vs* I$32,410 (95%CI: 32,370–32,440)]. The 95% CI of the two mean cost estimates do not overlap; hence, the difference is statistically significant.

Mental health condition when comorbid with diabetes incurred lower mean cost than when comorbid with a respiratory condition [I$20,750 (95% CI: 19,830–21,660) *vs* I$36,840 (95% CI: 36,440–37,250)] — the difference is statistically significant. However, when comorbid with heart/vascular condition, diabetes incurred higher mean cost than respiratory condition [I$37,090 (95% CI: 35,870–38,310) *vs* I$35,070 (95% CI: 34,890–35,260)] — the difference is statistically significant.

Our results further demonstrate that the costs of multimorbidity may not be additive of individual diseases. For example, the *average* cost of treating a patient with Diabetes + Kidney Disease (I$32,410) is observed to be larger than the *summed cost* of treating both {a patient with Hypertension + Diabetes (I$14,300) and a patient with Hypertension + Kidney Disease (I$17,740)}; however, this result is not statistically significant.

### Research question 4: Commonly included “cost ingredients”

Studies reporting total direct medical costs most frequently included costs of hospitalization (*n* = 57), outpatient care (*n* = 56), emergency care (*n* = 50), and drugs (*n* = 40). Other cost ingredients which were less common were those for consultations, diagnostics, surgery, medical equipment, specialist services, and therapy. For studies that reported direct non-medical costs (*n* = 2), food and transport costs were reported frequently, while costs of social care were rarely reported [51, 52]. Indirect costs (*n* = 7) principally focused on productivity loss, including wage and/or time loss, with a single study additionally including the interest from debts/loans [51–57, 89]. Detailed charting for cost ingredients is presented in Additional file 10.

## Discussion

Managing multimorbidity is expensive and imposes a considerable economic burden on both the health system and society. The finding that multimorbidity costs are positively associated with a country’s wealth highlights an urgent need for more evidence on the drivers of these costs. Our review further reveals a lack of balance in multimorbidity cost literature globally, which is dominated by US-based research, accounting for seven in every ten studies. Our review further examines how existing costing studies for multimorbidity are designed, and makes important recommendations for standardization in future research.

### Research question 1: Evidence on the costs of multimorbidity — and discussion of methodological issues and best practices in future costing studies

The availability of accurate and detailed costing studies will be essential in the coming decades to support global efforts to address multimorbidity. This review demonstrates, however, that there is a narrow scope of research and lack of methodological standardization contributing to variability between studies and impeding meaningful comparisons.

Specifically, focusing on index disease(s) and a few pre-selected comorbidities —for example [54, 66, 67, 69, 95] — rather than exploring the costs of possible combinations from a broader population perspective limits findings to few combinations. This makes it difficult to understand the full spectrum of the economic burden of multimorbidity in a country and create a basis for comparison between countries.

Moreover, the majority of studies obtained their costs from standalone data sources. Such an approach cannot provide a holistic view of healthcare costs including aspects such as non-medical and indirect costs [104]. A “bottom-up” approach to estimating the costs of chronic diseases using a medico-administrative database would be optimal in costing for multimorbidity [105]. Patient surveys have been found to underestimate costs, and ultimately, these should be used in conjunction with other data sources to correct for recall bias and to capture any costs unknown to healthcare users [106]. The practice of linking health data across multiple interfaces [15] and steps to linking population health data with administrative claims database have been endorsed [107].

Furthermore, with costs markedly higher in the 6-month windows before death and after diagnosis, further studies taking a longitudinal design would provide a clearer understanding of how costs and drivers evolve along the disease trajectory [21]. Understanding these transition stages is essential in the design and targeting of appropriate interventions [27].

The clear lack of agreement on the scope of health services to include, or which ingredients should be costed, along with a diverse array of methodologies and definitions contribute to heterogeneity between studies. A recommended guideline to collecting and estimating costs of multimorbidity may help in standardizing the definitions, process, and components and reducing the level of variability between studies [21, 48].

Finally, the scarcity of studies on the cost of multimorbidity from LMICs raises concern over equity. LMICs have become the new hub for NCDs and more research is imperative to setting out priority health agendas and informing the re-organization of healthcare delivery to support patients with multiple needs [13].

### Research question 2: The variation in costs across countries

To contextualize the possible drivers of the difference in costs among different country settings, we used GDP per capita as a determinant. While the data available for these analyses were again skewed by the predominance of American studies (14 out of 28 data points from the US), our regression showed that costs were generally higher in countries with higher GDP per capita [70]. Other studies on the costs of single chronic diseases such as diabetes have also pointed to similar findings [108]. This is not to be unexpected, and consistent with the fact that countries with higher GDP tend to have higher government spending on healthcare, driving up healthcare utilizations and costs [108]. However, the large variation in cost within a country (in this case, the US) also calls for in-depth assessment of the cost drivers of multimorbidity within a health system (system-level factors). The organization of care, e.g., standard *vs* integrated care, may play an important factor, though was not reported in the studies.

The high in-group variability of the costs of dyads (even when they all originate from the US — see Additional file 8) is in part due to the fact that these dyads were constructed as generic disease groups. For example, there are various cancer sites and mental health conditions with different levels of severity, treatment, duration, etc., which results in variability in costs. Additionally, the in-group variability for costs in the US might be explained by the use of different data sources and other study characteristics (study-level factors).

### Research question 3: Meta-analyses of the costs of the most frequently reported disease combinations and their variation

Although the cost of multimorbidity in part depends on the stage of the diseases alongside other factors, the knowledge of high-cost/low-cost disease combinations may inform the development of new integrated care models, where patients are classified according to their short-term/long-term need for specialist/routine care.

Using a fixed-effects model, results from the meta-analyses showed that patients with Cancer + Mental Health within the first year of cancer diagnosis had the highest average annual direct medical costs. This figure (I$85,820) is double that of another US study included in this review, which reported the cost of cancer and mental health not specific to any stage of the cascade of care (I$43,320) [103]. Indeed, treating cancers is expensive as research and development for cancer drugs and therapies are still ongoing [109] and it is even higher still during the 6–12 months following diagnosis compared to the period before or after that [110–112]. When comorbid with depression, the overall healthcare cost of treating cancer patients has been found to increase by 113% compared to non-depressed cancer patients [113].

Dyads involving hypertension were among the least expensive to manage. For patients with kidney disease, having a comorbidity of hypertension costs less than half of having a comorbidity of diabetes. Similarly, in the case of patients with a heart/vascular condition, having hypertension costs around half of having a respiratory condition. Specifically among hypertensive patients, treatment for musculoskeletal disorder *or* diabetes is cheaper than kidney disease *or* heart/vascular condition. A study found that based on the number of episodes that occurred within a period, and the frequency of transition from primary to specialist or emergency care, conditions such as hypertension may indicate “low severity of healthcare impact” and conditions such as kidney disease, heart/vascular disease, and respiratory disease may indicate “high severity”; thus the effect on costs [13]. Many chronic conditions feature commonly in multimorbidity dyads, and whether the cost of a disease combination is more or less than the additive costs of the component diseases needs to be further explored.

Although all studies included in the meta-analyses originated from the US, our findings provide valuable data on the differences in costs for treating different disease combinations within the American healthcare system. Though healthcare systems vary considerably between countries, the difference in costs between these 11 dyads in the US provides an indication of how they may be in other countries; however, more research in this area is needed.

### Research question 4: Commonly included “cost ingredients”

Our review highlights several missing pieces of the multimorbidity burden puzzle, notably that non-medical and indirect costs of multimorbidity are often not costed for. Particularly, social care is considered an important element of care for multimorbid patients given the long-term spectrum and complexity of illness, which may have lasting effects on their care needs [114]. In some settings, research has shown that social care cost may drive total care cost more than healthcare cost itself [18]. However, costing studies on multimorbidity which examine the component of social care/home care are still by and large limited [18, 115, 116]. This aspect is critical to understanding the full spectrum of multimorbidity costs to the system, especially when this has implications for vulnerable groups in society [27].

The most frequently reported cost ingredients identified were outpatient, inpatient, emergency care, and drugs. However, across studies, certain cost ingredients may overlap with one another (e.g., outpatient and GP visits, hospitalization and inpatient services, diagnostics and testing). Due to the ambiguous use of terms, it is difficult to clearly pinpoint the definition of the specific cost ingredients and what they entail.

### Strengths and limitations

To the best of our knowledge, our review is the first to systematically collect and quantitatively synthesize costs for disease combinations with comparability. We screened a large number of articles having conducted an extensive search. Beyond answering our research questions, we have additionally provided valuable insights into commonalities and inconsistencies in underlying methodologies between studies, and the ways in which these studies reported costs.

Our study also has several limitations. Firstly, our categorizations of diseases were made in line with health system organizational considerations, grouping conditions at an organ system level within medical specialties. This enabled us to reduce the number of combinations enabling meaningful interpretations; however, alternate groupings would have been possible, and those following a more treatment-focused perspective may have led to variation in results. On the other hand, we categorized *hypertension* separately from the heart/vascular group as it is a major risk factor for the latter. Most studies also report hypertension separately; therefore, following this approach allows for cross-comparison between studies.

In the meta-analyses, the degree of heterogeneity and variation of the underlying studies limited the number of studies included, all of which were from the US. Several sub-group analyses (e.g., Respiratory + Mental health, Respiratory + Heart/vascular condition) comprised only two data points. This may have limited the plausibility of the assumed approximated normality of mean costs. Furthermore, high *I*^2^ values were estimated for all disease combinations indicating a high degree of between-studies heterogeneity. Despite this, these results may still provide the best available insights though need to be interpreted in light of the highlighted methodological challenges.

The limited availability of studies from other countries led to the country comparison being dominated by studies from the US, with variation in these costs. More data from other countries are required to further examine this relationship.

Overall, we acknowledge that costs of multimorbidity may depend on many different factors including and beyond those that have been discussed in this paper. These may include individual-level factors (e.g., socio-demographic characteristics of patients, the number and severity of comorbidities, type and duration of healthcare intervention, access to care), system-level factors (e.g., the country, health system financing structure, level of care — e.g., public *vs* private, primary *vs* secondary care), and study-level factors (e.g., sampling error, costing perspective, data source, choice of cost ingredients). These details, however, were frequently unavailable, or not available to a sufficient level (e.g., severity of conditions, duration of care, type of health facility, etc.) to allow for structural reporting. Therefore, we were unable to control for some of these factors in the analysis. Despite these limitations, our review has revealed important findings.

## Conclusions

This review provides valuable insights into the costs across multimorbid health profiles, highlighting where priorities should be aligned to combat the escalating and complex economic burden of multimorbidity. These timely findings are essential for informing both the implementation of health interventions and the restructuring of healthcare delivery for multimorbid patients.

Multimorbidity imposes a large economic burden on both the health system and society, most notably for patients with cancer and mental health condition in the first year after cancer diagnosis. Whether the cost of a disease combination is more or less than the additive costs of the component diseases needs to be further explored.

The scope of research on the costs of multimorbidity is still narrow. Previous studies have typically only considered a narrow range of disease combinations, with data often obtained from single sources. Research to date has originated from a small pool of countries, with a striking lack of costing studies on multimorbidity from LMICs and Europe. Further rigorous and standardized methods of data collection and costing are essential to provide more comprehensive and comparable evidence of the cost of multimorbidity.

## Supplementary Information

**Additional file 1.** Search strings

**Additional file 2.** Screening checklists

**Additional file 3.** Data cleaning flow chart

**Additional file 4.** Definition of terminologies

**Additional file 5.** Charting of study characteristics

**Additional file 6.** The cost conversion table

**Additional file 7: Table S2.** Evidence on the costs of multimorbidity

**Additional file 8.** Checklist and data used for the country comparison

**Additional file 9.** Homogeneity checklist and data used for the meta-analyses

**Additional file 10.** Charting of cost ingredients

## Data Availability

Data and materials will be made available upon request.

## Abbreviations

AIC: Akaike Information Criterion
COPD: Chronic obstructive pulmonary disease
GDP: Gross Domestic Product
I$: International dollars
LMICs: Low- and middle-income countries
MEPS: Medical Expenditure Panel Survey
NCDs: Noncommunicable diseases
NOS: Newcastle–Ottawa Quality Assessment
PPP: Purchasing Power Parities
PRISMA: Preferred Reporting Items for Systematic Reviews and Meta-Analyses
TB: Tuberculosis
US: United States
