# Supplementary material for: Costs of multimorbidity: a systematic review and meta-analyses
Source: BMC Med. 2022 Jul 19;20:234. doi: 10.1186/s12916-022-02427-9 (PMC9295506; doi:10.1186/s12916-022-02427-9)
Supplement: Supplementary file 1 — Additional file 1. Search strings [file 12916_2022_2427_MOESM1_ESM.docx]

Additional file 2: Search strings

Cochrane / Evidence based medicine reviews

1 (pulmonary disease, chronic obstructive/ or (copd or (pulmonary adj2 (disease? or disorder?))).ti,ab.) and (diabetes mellitus/ or diabet*.ti,ab. or hypertension/ or (hypertens* or "high blood pressure?").ti,ab. or neoplasms/ or (neoplasm? or cancer?).ti,ab. or coronary disease/ or ((cardiac or cardiovascular or coronary) adj1 (disease? or disorder? or failure)).ti,ab. or exp myocardial infarction/ or (myocardial infarct* or cardiovascular strok*).ti,ab. or exp heart failure/ or ((heart or cardiac or myocardial) adj1 failure).ti,ab. or asthma/ or asthma*.ti,ab. or anxiety/ or depression/ or (((mental or anxiety or mood or psychological or sleep) adj1 (disease? or disorder?)) or depression?).ti,ab. or osteoporosis/ or osteoporosis.ti,ab. or exp dyslipidemias/ or (dyslipid?emia* or hyperlipid?emia* or hypercholesterolemia* or hypertriglyceridemia*).ti,ab. or exp thyroid diseases/ or ((thyroid adj1 (disease? or disorder?)) or hyperthyroid* or hypothyroid*).ti,ab. or joint diseases/ or osteoarthritis/ or arthritis rheumatoid/ or (joint disease* or rheumatoid arthritis or osteoarthritis).ti,ab. or exp kidney diseases/ or (kidney adj1 (disease? or disorder?)).ti,ab. or epilepsy/ or (epilep* or seizure?).ti,ab. or hiv infections/ or (hiv or acquired immun* deficiency syndrome? or aids).ti,ab. or exp liver diseases/ or (liver adj1 (disease? or disorder?)).ti,ab. or stroke/ or stroke*.ti,ab. or dementia/ or dementia.ti,ab. or exp arrhythmias, cardiac/ or cardiac arrhythmia*.ti,ab. or anemia/ or an?emia*.ti,ab. or obesity/ or (obesity or obese).ti,ab. or prostatic hyperplasia/ or (prostatic adj1 (hyperplasia or hypertrophy)).ti,ab. or tuberculosis/ or tuberculosis.ti,ab. or exp chronic hepatitis/ or chronic hepatitis.ti,ab. or substance-related disorders/ or (((substance or drug or marijuana or cocaine or amphetamine) adj2 abuse) or "substance abuse" or addiction?).ti,ab. or inflammatory bowel diseases/ or (inflammatory bowel disease? or ulcerative colitis or crohn or ibd).ti,ab. or deafness/ or deaf*.ti,ab. or blindness/ or (blind* or (visual adj1 loss)).ti,ab. or parkinson disease/ or parkinson*.ti,ab. or exp autism spectrum disorder/ or autis*.ti,ab.)

2 (diabetes mellitus/ or diabet*.ti,ab.) AND (hypertension/ or (hypertens* or "high blood pressure?").ti,ab. or neoplasms/ or (neoplasm? or cancer?).ti,ab. or coronary disease/ or ((cardiac or cardiovascular or coronary) adj1 (disease? or disorder? or failure)).ti,ab. or exp myocardial infarction/ or (myocardial infarct* or cardiovascular strok*).ti,ab. or exp heart failure/ or ((heart or cardiac or myocardial) adj1 failure).ti,ab. or asthma/ or asthma*.ti,ab. or anxiety/ or depression/ or (((mental or anxiety or mood or psychological or sleep) adj1 (disease? or disorder?)) or depression?).ti,ab. or osteoporosis/ or osteoporosis.ti,ab. or exp dyslipidemias/ or (dyslipid?emia* or hyperlipid?emia* or hypercholesterolemia* or hypertriglyceridemia*).ti,ab. or exp thyroid diseases/ or ((thyroid adj1 (disease? or disorder?)) or hyperthyroid* or hypothyroid*).ti,ab. or joint diseases/ or osteoarthritis/ or arthritis rheumatoid/ or (joint disease* or rheumatoid arthritis or osteoarthritis).ti,ab. or exp kidney diseases/ or (kidney adj1 (disease? or disorder?)).ti,ab. or epilepsy/ or (epilep* or seizure?).ti,ab. or hiv infections/ or (hiv or acquired immun* deficiency syndrome? or aids).ti,ab. or exp liver diseases/ or (liver adj1 (disease? or disorder?)).ti,ab. or stroke/ or stroke*.ti,ab. or dementia/ or dementia.ti,ab. or exp arrhythmias, cardiac/ or cardiac arrhythmia*.ti,ab. or anemia/ or an?emia*.ti,ab. or obesity/ or (obesity or obese).ti,ab. or prostatic hyperplasia/ or (prostatic adj1 (hyperplasia or hypertrophy)).ti,ab. or tuberculosis/ or tuberculosis.ti,ab. or exp chronic hepatitis/ or chronic hepatitis.ti,ab. or substance-related disorders/ or (((substance or drug or marijuana or cocaine or amphetamine) adj2 abuse) or "substance abuse" or addiction?).ti,ab. or inflammatory bowel diseases/ or (inflammatory bowel disease? or ulcerative colitis or crohn or ibd).ti,ab. or deafness/ or deaf*.ti,ab. or blindness/ or (blind* or (visual adj1 loss)).ti,ab. or parkinson disease/ or parkinson*.ti,ab. or exp autism spectrum disorder/ or autis*.ti,ab.)

3 (neoplasms/ or (neoplasm? or cancer?).ti,ab.) and (coronary disease/ or ((cardiac or cardiovascular or coronary) adj1 (disease? or disorder? or failure)).ti,ab. or exp myocardial infarction/ or (myocardial infarct* or cardiovascular strok*).ti,ab. or exp heart failure/ or ((heart or cardiac or myocardial) adj1 failure).ti,ab. or asthma/ or asthma*.ti,ab. or anxiety/ or depression/ or (((mental or anxiety or mood or psychological or sleep) adj1 (disease? or disorder?)) or depression?).ti,ab. or osteoporosis/ or osteoporosis.ti,ab. or exp dyslipidemias/ or (dyslipid?emia* or hyperlipid?emia* or hypercholesterolemia* or hypertriglyceridemia*).ti,ab. or exp thyroid diseases/ or ((thyroid adj1 (disease? or disorder?)) or hyperthyroid* or hypothyroid*).ti,ab. or joint diseases/ or osteoarthritis/ or arthritis rheumatoid/ or (joint disease* or rheumatoid arthritis or osteoarthritis).ti,ab. or exp kidney diseases/ or (kidney adj1 (disease? or disorder?)).ti,ab. or epilepsy/ or (epilep* or seizure?).ti,ab. or hiv infections/ or (hiv or acquired immun* deficiency syndrome? or aids).ti,ab. or exp liver diseases/ or (liver adj1 (disease? or disorder?)).ti,ab. or stroke/ or stroke*.ti,ab. or dementia/ or dementia.ti,ab. or exp arrhythmias, cardiac/ or cardiac arrhythmia*.ti,ab. or anemia/ or an?emia*.ti,ab. or obesity/ or (obesity or obese).ti,ab. or prostatic hyperplasia/ or (prostatic adj1 (hyperplasia or hypertrophy)).ti,ab. or tuberculosis/ or tuberculosis.ti,ab. or exp chronic hepatitis/ or chronic hepatitis.ti,ab. or substance-related disorders/ or (((substance or drug or marijuana or cocaine or amphetamine) adj2 abuse) or "substance abuse" or addiction?).ti,ab. or inflammatory bowel diseases/ or (inflammatory bowel disease? or ulcerative colitis or crohn or ibd).ti,ab. or deafness/ or deaf*.ti,ab. or blindness/ or (blind* or (visual adj1 loss)).ti,ab. or parkinson disease/ or parkinson*.ti,ab. or exp autism spectrum disorder/ or autis*.ti,ab.)

4 (hypertension/ or (hypertens* or "high blood pressure?").ti,ab.) and (neoplasms/ or (neoplasm? or cancer?).ti,ab. or coronary disease/ or ((cardiac or cardiovascular or coronary) adj1 (disease? or disorder? or failure)).ti,ab. or exp myocardial infarction/ or (myocardial infarct* or cardiovascular strok*).ti,ab. or exp heart failure/ or ((heart or cardiac or myocardial) adj1 failure).ti,ab. or asthma/ or asthma*.ti,ab. or anxiety/ or depression/ or (((mental or anxiety or mood or psychological or sleep) adj1 (disease? or disorder?)) or depression?).ti,ab. or osteoporosis/ or osteoporosis.ti,ab. or exp dyslipidemias/ or (dyslipid?emia* or hyperlipid?emia* or hypercholesterolemia* or hypertriglyceridemia*).ti,ab. or exp thyroid diseases/ or ((thyroid adj1 (disease? or disorder?)) or hyperthyroid* or hypothyroid*).ti,ab. or joint diseases/ or osteoarthritis/ or arthritis rheumatoid/ or (joint disease* or rheumatoid arthritis or osteoarthritis).ti,ab. or exp kidney diseases/ or (kidney adj1 (disease? or disorder?)).ti,ab. or epilepsy/ or (epilep* or seizure?).ti,ab. or hiv infections/ or (hiv or acquired immun* deficiency syndrome? or aids).ti,ab. or exp liver diseases/ or (liver adj1 (disease? or disorder?)).ti,ab. or stroke/ or stroke*.ti,ab. or dementia/ or dementia.ti,ab. or exp arrhythmias, cardiac/ or cardiac arrhythmia*.ti,ab. or anemia/ or an?emia*.ti,ab. or obesity/ or (obesity or obese).ti,ab. or prostatic hyperplasia/ or (prostatic adj1 (hyperplasia or hypertrophy)).ti,ab. or tuberculosis/ or tuberculosis.ti,ab. or exp chronic hepatitis/ or chronic hepatitis.ti,ab. or substance-related disorders/ or (((substance or drug or marijuana or cocaine or amphetamine) adj2 abuse) or "substance abuse" or addiction?).ti,ab. or inflammatory bowel diseases/ or (inflammatory bowel disease? or ulcerative colitis or crohn or ibd).ti,ab. or deafness/ or deaf*.ti,ab. or blindness/ or (blind* or (visual adj1 loss)).ti,ab. or parkinson disease/ or parkinson*.ti,ab. or exp autism spectrum disorder/ or autis*.ti,ab.)

5 (coronary disease/ or ((cardiac or cardiovascular or coronary) adj1 (disease? or disorder? or failure)).ti,ab.) and (exp Myocardial Infarction/ or (Myocardial Infarct* or cardiovascular strok*).ti,ab. or exp heart failure/ or ((heart or cardiac or myocardial) adj1 failure).ti,ab. or asthma/ or asthma*.ti,ab. or anxiety/ or depression/ or (((mental or anxiety or mood or psychological or sleep) adj1 (disease? or disorder?)) or depression?).ti,ab. or osteoporosis/ or osteoporosis.ti,ab. or exp dyslipidemias/ or (dyslipid?emia* or hyperlipid?emia* or hypercholesterolemia* or hypertriglyceridemia*).ti,ab. or exp thyroid diseases/ or ((thyroid adj1 (disease? or disorder?)) or hyperthyroid* or hypothyroid*).ti,ab. or joint diseases/ or osteoarthritis/ or arthritis rheumatoid/ or (joint disease* or rheumatoid arthritis or osteoarthritis).ti,ab. or exp kidney diseases/ or (kidney adj1 (disease? or disorder?)).ti,ab. or epilepsy/ or (epilep* or seizure?).ti,ab. or hiv infections/ or (HIV or acquired immun* deficiency syndrome? or aids).ti,ab. or exp liver diseases/ or (liver adj1 (disease? or disorder?)).ti,ab. or stroke/ or stroke*.ti,ab. or dementia/ or dementia.ti,ab. or exp arrhythmias, cardiac/ or cardiac arrhythmia*.ti,ab. or anemia/ or an?emia*.ti,ab. or obesity/ or (obesity or obese).ti,ab. or prostatic hyperplasia/ or (prostatic adj1 (hyperplasia or hypertrophy)).ti,ab. or tuberculosis/ or tuberculosis.ti,ab. or exp chronic hepatitis/ or chronic hepatitis.ti,ab. or Substance-Related Disorders/ or (((substance or drug or marijuana or cocaine or amphetamine) adj2 abuse) or "substance abuse" or addiction?).ti,ab. or Inflammatory Bowel Diseases/ or (Inflammatory Bowel Disease? or ulcerative colitis or crohn or IBD).ti,ab. or deafness/ or deaf*.ti,ab. or blindness/ or (blind* or (visual adj1 loss)).ti,ab. or Parkinson Disease/ or parkinson*.ti,ab. or exp Autism Spectrum Disorder/ or autis*.ti,ab.)

6 (exp myocardial infarction/ or (myocardial infarct* or cardiovascular strok*).ti,ab.) and (exp heart failure/ or ((heart or cardiac or myocardial) adj1 failure).ti,ab. or asthma/ or asthma*.ti,ab. or anxiety/ or depression/ or (((mental or anxiety or mood or psychological or sleep) adj1 (disease? or disorder?)) or depression?).ti,ab. or osteoporosis/ or osteoporosis.ti,ab. or exp dyslipidemias/ or (dyslipid?emia* or hyperlipid?emia* or hypercholesterolemia* or hypertriglyceridemia*).ti,ab. or exp thyroid diseases/ or ((thyroid adj1 (disease? or disorder?)) or hyperthyroid* or hypothyroid*).ti,ab. or joint diseases/ or osteoarthritis/ or arthritis rheumatoid/ or (joint disease* or rheumatoid arthritis or osteoarthritis).ti,ab. or exp kidney diseases/ or (kidney adj1 (disease? or disorder?)).ti,ab. or epilepsy/ or (epilep* or seizure?).ti,ab. or hiv infections/ or (hiv or acquired immun* deficiency syndrome? or aids).ti,ab. or exp liver diseases/ or (liver adj1 (disease? or disorder?)).ti,ab. or stroke/ or stroke*.ti,ab. or dementia/ or dementia.ti,ab. or exp arrhythmias, cardiac/ or cardiac arrhythmia*.ti,ab. or anemia/ or an?emia*.ti,ab. or obesity/ or (obesity or obese).ti,ab. or prostatic hyperplasia/ or (prostatic adj1 (hyperplasia or hypertrophy)).ti,ab. or tuberculosis/ or tuberculosis.ti,ab. or exp chronic hepatitis/ or chronic hepatitis.ti,ab. or substance-related disorders/ or (((substance or drug or marijuana or cocaine or amphetamine) adj2 abuse) or "substance abuse" or addiction?).ti,ab. or inflammatory bowel diseases/ or (inflammatory bowel disease? or ulcerative colitis or crohn or ibd).ti,ab. or deafness/ or deaf*.ti,ab. or blindness/ or (blind* or (visual adj1 loss)).ti,ab. or parkinson disease/ or parkinson*.ti,ab. or exp autism spectrum disorder/ or autis*.ti,ab.)

7 (exp heart failure/ or ((heart or cardiac or myocardial) adj1 failure).ti,ab.) and (asthma/ or asthma*.ti,ab. or anxiety/ or depression/ or (((mental or anxiety or mood or psychological or sleep) adj1 (disease? or disorder?)) or depression?).ti,ab. or osteoporosis/ or osteoporosis.ti,ab. or exp dyslipidemias/ or (dyslipid?emia* or hyperlipid?emia* or hypercholesterolemia* or hypertriglyceridemia*).ti,ab. or exp thyroid diseases/ or ((thyroid adj1 (disease? or disorder?)) or hyperthyroid* or hypothyroid*).ti,ab. or joint diseases/ or osteoarthritis/ or arthritis rheumatoid/ or (joint disease* or rheumatoid arthritis or osteoarthritis).ti,ab. or exp kidney diseases/ or (kidney adj1 (disease? or disorder?)).ti,ab. or epilepsy/ or (epilep* or seizure?).ti,ab. or hiv infections/ or (hiv or acquired immun* deficiency syndrome? or aids).ti,ab. or exp liver diseases/ or (liver adj1 (disease? or disorder?)).ti,ab. or stroke/ or stroke*.ti,ab. or dementia/ or dementia.ti,ab. or exp arrhythmias, cardiac/ or cardiac arrhythmia*.ti,ab. or anemia/ or an?emia*.ti,ab. or obesity/ or (obesity or obese).ti,ab. or prostatic hyperplasia/ or (prostatic adj1 (hyperplasia or hypertrophy)).ti,ab. or tuberculosis/ or tuberculosis.ti,ab. or exp chronic hepatitis/ or chronic hepatitis.ti,ab. or substance-related disorders/ or (((substance or drug or marijuana or cocaine or amphetamine) adj2 abuse) or "substance abuse" or addiction?).ti,ab. or inflammatory bowel diseases/ or (inflammatory bowel disease? or ulcerative colitis or crohn or ibd).ti,ab. or deafness/ or deaf*.ti,ab. or blindness/ or (blind* or (visual adj1 loss)).ti,ab. or parkinson disease/ or parkinson*.ti,ab. or exp autism spectrum disorder/ or autis*.ti,ab.)

8 (asthma/ or asthma*.ti,ab.) and (anxiety/ or depression/ or (((mental or anxiety or mood or psychological or sleep) adj1 (disease? or disorder?)) or depression?).ti,ab. or osteoporosis/ or osteoporosis.ti,ab. or exp dyslipidemias/ or (dyslipid?emia* or hyperlipid?emia* or hypercholesterolemia* or hypertriglyceridemia*).ti,ab. or exp thyroid diseases/ or ((thyroid adj1 (disease? or disorder?)) or hyperthyroid* or hypothyroid*).ti,ab. or joint diseases/ or osteoarthritis/ or arthritis rheumatoid/ or (joint disease* or rheumatoid arthritis or osteoarthritis).ti,ab. or exp kidney diseases/ or (kidney adj1 (disease? or disorder?)).ti,ab. or epilepsy/ or (epilep* or seizure?).ti,ab. or hiv infections/ or (hiv or acquired immun* deficiency syndrome? or aids).ti,ab. or exp liver diseases/ or (liver adj1 (disease? or disorder?)).ti,ab. or stroke/ or stroke*.ti,ab. or dementia/ or dementia.ti,ab. or exp arrhythmias, cardiac/ or cardiac arrhythmia*.ti,ab. or anemia/ or an?emia*.ti,ab. or obesity/ or (obesity or obese).ti,ab. or prostatic hyperplasia/ or (prostatic adj1 (hyperplasia or hypertrophy)).ti,ab. or tuberculosis/ or tuberculosis.ti,ab. or exp chronic hepatitis/ or chronic hepatitis.ti,ab. or substance-related disorders/ or (((substance or drug or marijuana or cocaine or amphetamine) adj2 abuse) or "substance abuse" or addiction?).ti,ab. or inflammatory bowel diseases/ or (inflammatory bowel disease? or ulcerative colitis or crohn or ibd).ti,ab. or deafness/ or deaf*.ti,ab. or blindness/ or (blind* or (visual adj1 loss)).ti,ab. or parkinson disease/ or parkinson*.ti,ab. or exp autism spectrum disorder/ or autis*.ti,ab.)

9 (anxiety/ or depression/ or (((mental or anxiety or mood or psychological or sleep) adj1 (disease? or disorder?)) or depression?).ti,ab.) and (osteoporosis/ or osteoporosis.ti,ab. or exp dyslipidemias/ or (dyslipid?emia* or hyperlipid?emia* or hypercholesterolemia* or hypertriglyceridemia*).ti,ab. or exp thyroid diseases/ or ((thyroid adj1 (disease? or disorder?)) or hyperthyroid* or hypothyroid*).ti,ab. or joint diseases/ or osteoarthritis/ or arthritis rheumatoid/ or (joint disease* or rheumatoid arthritis or osteoarthritis).ti,ab. or exp kidney diseases/ or (kidney adj1 (disease? or disorder?)).ti,ab. or epilepsy/ or (epilep* or seizure?).ti,ab. or hiv infections/ or (hiv or acquired immun* deficiency syndrome? or aids).ti,ab. or exp liver diseases/ or (liver adj1 (disease? or disorder?)).ti,ab. or stroke/ or stroke*.ti,ab. or dementia/ or dementia.ti,ab. or exp arrhythmias, cardiac/ or cardiac arrhythmia*.ti,ab. or anemia/ or an?emia*.ti,ab. or obesity/ or (obesity or obese).ti,ab. or prostatic hyperplasia/ or (prostatic adj1 (hyperplasia or hypertrophy)).ti,ab. or tuberculosis/ or tuberculosis.ti,ab. or exp chronic hepatitis/ or chronic hepatitis.ti,ab. or substance-related disorders/ or (((substance or drug or marijuana or cocaine or amphetamine) adj2 abuse) or "substance abuse" or addiction?).ti,ab. or inflammatory bowel diseases/ or (inflammatory bowel disease? or ulcerative colitis or crohn or ibd).ti,ab. or deafness/ or deaf*.ti,ab. or blindness/ or (blind* or (visual adj1 loss)).ti,ab. or parkinson disease/ or parkinson*.ti,ab. or exp autism spectrum disorder/ or autis*.ti,ab.)

10 (osteoporosis/ or osteoporosis.ti,ab.) and (exp dyslipidemias/ or (dyslipid?emia* or hyperlipid?emia* or hypercholesterolemia* or hypertriglyceridemia*).ti,ab. or exp thyroid diseases/ or ((thyroid adj1 (disease? or disorder?)) or hyperthyroid* or hypothyroid*).ti,ab. or joint diseases/ or osteoarthritis/ or arthritis rheumatoid/ or (joint disease* or rheumatoid arthritis or osteoarthritis).ti,ab. or exp kidney diseases/ or (kidney adj1 (disease? or disorder?)).ti,ab. or epilepsy/ or (epilep* or seizure?).ti,ab. or hiv infections/ or (hiv or acquired immun* deficiency syndrome? or aids).ti,ab. or exp liver diseases/ or (liver adj1 (disease? or disorder?)).ti,ab. or stroke/ or stroke*.ti,ab. or dementia/ or dementia.ti,ab. or exp arrhythmias, cardiac/ or cardiac arrhythmia*.ti,ab. or anemia/ or an?emia*.ti,ab. or obesity/ or (obesity or obese).ti,ab. or prostatic hyperplasia/ or (prostatic adj1 (hyperplasia or hypertrophy)).ti,ab. or tuberculosis/ or tuberculosis.ti,ab. or exp chronic hepatitis/ or chronic hepatitis.ti,ab. or substance-related disorders/ or (((substance or drug or marijuana or cocaine or amphetamine) adj2 abuse) or "substance abuse" or addiction?).ti,ab. or inflammatory bowel diseases/ or (inflammatory bowel disease? or ulcerative colitis or crohn or ibd).ti,ab. or deafness/ or deaf*.ti,ab. or blindness/ or (blind* or (visual adj1 loss)).ti,ab. or parkinson disease/ or parkinson*.ti,ab. or exp autism spectrum disorder/ or autis*.ti,ab.)

11 (exp dyslipidemias/ or (dyslipid?emia* or hyperlipid?emia* or hypercholesterolemia* or hypertriglyceridemia*).ti,ab.) and (exp thyroid diseases/ or ((thyroid adj1 (disease? or disorder?)) or hyperthyroid* or hypothyroid*).ti,ab. or joint diseases/ or osteoarthritis/ or arthritis rheumatoid/ or (joint disease* or rheumatoid arthritis or osteoarthritis).ti,ab. or exp kidney diseases/ or (kidney adj1 (disease? or disorder?)).ti,ab. or epilepsy/ or (epilep* or seizure?).ti,ab. or hiv infections/ or (hiv or acquired immun* deficiency syndrome? or aids).ti,ab. or exp liver diseases/ or (liver adj1 (disease? or disorder?)).ti,ab. or stroke/ or stroke*.ti,ab. or dementia/ or dementia.ti,ab. or exp arrhythmias, cardiac/ or cardiac arrhythmia*.ti,ab. or anemia/ or an?emia*.ti,ab. or obesity/ or (obesity or obese).ti,ab. or prostatic hyperplasia/ or (prostatic adj1 (hyperplasia or hypertrophy)).ti,ab. or tuberculosis/ or tuberculosis.ti,ab. or exp chronic hepatitis/ or chronic hepatitis.ti,ab. or substance-related disorders/ or (((substance or drug or marijuana or cocaine or amphetamine) adj2 abuse) or "substance abuse" or addiction?).ti,ab. or inflammatory bowel diseases/ or (inflammatory bowel disease? or ulcerative colitis or crohn or ibd).ti,ab. or deafness/ or deaf*.ti,ab. or blindness/ or (blind* or (visual adj1 loss)).ti,ab. or parkinson disease/ or parkinson*.ti,ab. or exp autism spectrum disorder/ or autis*.ti,ab.)

12 (exp dyslipidemias/ or (dyslipid?emia* or hyperlipid?emia* or hypercholesterolemia* or hypertriglyceridemia*).ti,ab.) and (exp thyroid diseases/ or ((thyroid adj1 (disease? or disorder?)) or hyperthyroid* or hypothyroid*).ti,ab. or joint diseases/ or osteoarthritis/ or arthritis rheumatoid/ or (joint disease* or rheumatoid arthritis or osteoarthritis).ti,ab. or exp kidney diseases/ or (kidney adj1 (disease? or disorder?)).ti,ab. or epilepsy/ or (epilep* or seizure?).ti,ab. or hiv infections/ or (hiv or acquired immun* deficiency syndrome? or aids).ti,ab. or exp liver diseases/ or (liver adj1 (disease? or disorder?)).ti,ab. or stroke/ or stroke*.ti,ab. or dementia/ or dementia.ti,ab. or exp arrhythmias, cardiac/ or cardiac arrhythmia*.ti,ab. or anemia/ or an?emia*.ti,ab. or obesity/ or (obesity or obese).ti,ab. or prostatic hyperplasia/ or (prostatic adj1 (hyperplasia or hypertrophy)).ti,ab. or tuberculosis/ or tuberculosis.ti,ab. or exp chronic hepatitis/ or chronic hepatitis.ti,ab. or substance-related disorders/ or (((substance or drug or marijuana or cocaine or amphetamine) adj2 abuse) or "substance abuse" or addiction?).ti,ab. or inflammatory bowel diseases/ or (inflammatory bowel disease? or ulcerative colitis or crohn or ibd).ti,ab. or deafness/ or deaf*.ti,ab. or blindness/ or (blind* or (visual adj1 loss)).ti,ab. or parkinson disease/ or parkinson*.ti,ab. or exp autism spectrum disorder/ or autis*.ti,ab.)

13 (joint diseases/ or osteoarthritis/ or arthritis rheumatoid/ or (joint disease* or rheumatoid arthritis or osteoarthritis).ti,ab.) and (exp kidney diseases/ or (kidney adj1 (disease? or disorder?)).ti,ab. or epilepsy/ or (epilep* or seizure?).ti,ab. or hiv infections/ or (hiv or acquired immun* deficiency syndrome? or aids).ti,ab. or exp liver diseases/ or (liver adj1 (disease? or disorder?)).ti,ab. or stroke/ or stroke*.ti,ab. or dementia/ or dementia.ti,ab. or exp arrhythmias, cardiac/ or cardiac arrhythmia*.ti,ab. or anemia/ or an?emia*.ti,ab. or obesity/ or (obesity or obese).ti,ab. or prostatic hyperplasia/ or (prostatic adj1 (hyperplasia or hypertrophy)).ti,ab. or tuberculosis/ or tuberculosis.ti,ab. or exp chronic hepatitis/ or chronic hepatitis.ti,ab. or substance-related disorders/ or (((substance or drug or marijuana or cocaine or amphetamine) adj2 abuse) or "substance abuse" or addiction?).ti,ab. or inflammatory bowel diseases/ or (inflammatory bowel disease? or ulcerative colitis or crohn or ibd).ti,ab. or deafness/ or deaf*.ti,ab. or blindness/ or (blind* or (visual adj1 loss)).ti,ab. or parkinson disease/ or parkinson*.ti,ab. or exp autism spectrum disorder/ or autis*.ti,ab.)

14 (exp kidney diseases/ or (kidney adj1 (disease? or disorder?)).ti,ab.) and (epilepsy/ or (epilep* or seizure?).ti,ab. or hiv infections/ or (hiv or acquired immun* deficiency syndrome? or aids).ti,ab. or exp liver diseases/ or (liver adj1 (disease? or disorder?)).ti,ab. or stroke/ or stroke*.ti,ab. or dementia/ or dementia.ti,ab. or exp arrhythmias, cardiac/ or cardiac arrhythmia*.ti,ab. or anemia/ or an?emia*.ti,ab. or obesity/ or (obesity or obese).ti,ab. or prostatic hyperplasia/ or (prostatic adj1 (hyperplasia or hypertrophy)).ti,ab. or tuberculosis/ or tuberculosis.ti,ab. or exp chronic hepatitis/ or chronic hepatitis.ti,ab. or substance-related disorders/ or (((substance or drug or marijuana or cocaine or amphetamine) adj2 abuse) or "substance abuse" or addiction?).ti,ab. or inflammatory bowel diseases/ or (inflammatory bowel disease? or ulcerative colitis or crohn or ibd).ti,ab. or deafness/ or deaf*.ti,ab. or blindness/ or (blind* or (visual adj1 loss)).ti,ab. or parkinson disease/ or parkinson*.ti,ab. or exp autism spectrum disorder/ or autis*.ti,ab.)

15 (epilepsy/ or (epilep* or seizure?).ti,ab.) and (hiv infections/ or (hiv or acquired immun* deficiency syndrome? or aids).ti,ab. or exp liver diseases/ or (liver adj1 (disease? or disorder?)).ti,ab. or stroke/ or stroke*.ti,ab. or dementia/ or dementia.ti,ab. or exp arrhythmias, cardiac/ or cardiac arrhythmia*.ti,ab. or anemia/ or an?emia*.ti,ab. or obesity/ or (obesity or obese).ti,ab. or prostatic hyperplasia/ or (prostatic adj1 (hyperplasia or hypertrophy)).ti,ab. or tuberculosis/ or tuberculosis.ti,ab. or exp chronic hepatitis/ or chronic hepatitis.ti,ab. or substance-related disorders/ or (((substance or drug or marijuana or cocaine or amphetamine) adj2 abuse) or "substance abuse" or addiction?).ti,ab. or inflammatory bowel diseases/ or (inflammatory bowel disease? or ulcerative colitis or crohn or ibd).ti,ab. or deafness/ or deaf*.ti,ab. or blindness/ or (blind* or (visual adj1 loss)).ti,ab. or parkinson disease/ or parkinson*.ti,ab. or exp autism spectrum disorder/ or autis*.ti,ab.)

16 (hiv infections/ or (hiv or acquired immun* deficiency syndrome? or aids).ti,ab.) and (exp liver diseases/ or (liver adj1 (disease? or disorder?)).ti,ab. or stroke/ or stroke*.ti,ab. or dementia/ or dementia.ti,ab. or exp arrhythmias, cardiac/ or cardiac arrhythmia*.ti,ab. or anemia/ or an?emia*.ti,ab. or obesity/ or (obesity or obese).ti,ab. or prostatic hyperplasia/ or (prostatic adj1 (hyperplasia or hypertrophy)).ti,ab. or tuberculosis/ or tuberculosis.ti,ab. or exp chronic hepatitis/ or chronic hepatitis.ti,ab. or substance-related disorders/ or (((substance or drug or marijuana or cocaine or amphetamine) adj2 abuse) or "substance abuse" or addiction?).ti,ab. or inflammatory bowel diseases/ or (inflammatory bowel disease? or ulcerative colitis or crohn or ibd).ti,ab. or deafness/ or deaf*.ti,ab. or blindness/ or (blind* or (visual adj1 loss)).ti,ab. or parkinson disease/ or parkinson*.ti,ab. or exp autism spectrum disorder/ or autis*.ti,ab.)

17 (exp liver diseases/ or (liver adj1 (disease? or disorder?)).ti,ab.) and (stroke/ or stroke*.ti,ab. or dementia/ or dementia.ti,ab. or exp arrhythmias, cardiac/ or cardiac arrhythmia*.ti,ab. or anemia/ or an?emia*.ti,ab. or obesity/ or (obesity or obese).ti,ab. or prostatic hyperplasia/ or (prostatic adj1 (hyperplasia or hypertrophy)).ti,ab. or tuberculosis/ or tuberculosis.ti,ab. or exp chronic hepatitis/ or chronic hepatitis.ti,ab. or substance-related disorders/ or (((substance or drug or marijuana or cocaine or amphetamine) adj2 abuse) or "substance abuse" or addiction?).ti,ab. or inflammatory bowel diseases/ or (inflammatory bowel disease? or ulcerative colitis or crohn or ibd).ti,ab. or deafness/ or deaf*.ti,ab. or blindness/ or (blind* or (visual adj1 loss)).ti,ab. or parkinson disease/ or parkinson*.ti,ab. or exp autism spectrum disorder/ or autis*.ti,ab.)

18 (stroke/ or stroke*.ti,ab.) and (dementia/ or dementia.ti,ab. or exp arrhythmias, cardiac/ or cardiac arrhythmia*.ti,ab. or anemia/ or an?emia*.ti,ab. or obesity/ or (obesity or obese).ti,ab. or prostatic hyperplasia/ or (prostatic adj1 (hyperplasia or hypertrophy)).ti,ab. or tuberculosis/ or tuberculosis.ti,ab. or exp chronic hepatitis/ or chronic hepatitis.ti,ab. or substance-related disorders/ or (((substance or drug or marijuana or cocaine or amphetamine) adj2 abuse) or "substance abuse" or addiction?).ti,ab. or inflammatory bowel diseases/ or (inflammatory bowel disease? or ulcerative colitis or crohn or ibd).ti,ab. or deafness/ or deaf*.ti,ab. or blindness/ or (blind* or (visual adj1 loss)).ti,ab. or parkinson disease/ or parkinson*.ti,ab. or exp autism spectrum disorder/ or autis*.ti,ab.)

19 (dementia/ or dementia.ti,ab.) and (exp arrhythmias, cardiac/ or cardiac arrhythmia*.ti,ab. or anemia/ or an?emia*.ti,ab. or obesity/ or (obesity or obese).ti,ab. or prostatic hyperplasia/ or (prostatic adj1 (hyperplasia or hypertrophy)).ti,ab. or tuberculosis/ or tuberculosis.ti,ab. or exp chronic hepatitis/ or chronic hepatitis.ti,ab. or Substance-Related Disorders/ or (((substance or drug or marijuana or cocaine or amphetamine) adj2 abuse) or "substance abuse" or addiction?).ti,ab. or Inflammatory Bowel Diseases/ or (Inflammatory Bowel Disease? or ulcerative colitis or crohn or IBD).ti,ab. or deafness/ or deaf*.ti,ab. or blindness/ or (blind* or (visual adj1 loss)).ti,ab. or Parkinson Disease/ or parkinson*.ti,ab. or exp Autism Spectrum Disorder/ or autis*.ti,ab.)

20 (exp arrhythmias, cardiac/ or cardiac arrhythmia*.ti,ab.) and (anemia/ or an?emia*.ti,ab. or obesity/ or (obesity or obese).ti,ab. or prostatic hyperplasia/ or (prostatic adj1 (hyperplasia or hypertrophy)).ti,ab. or tuberculosis/ or tuberculosis.ti,ab. or exp chronic hepatitis/ or chronic hepatitis.ti,ab. or substance-related disorders/ or (((substance or drug or marijuana or cocaine or amphetamine) adj2 abuse) or "substance abuse" or addiction?).ti,ab. or inflammatory bowel diseases/ or (inflammatory bowel disease? or ulcerative colitis or crohn or ibd).ti,ab. or deafness/ or deaf*.ti,ab. or blindness/ or (blind* or (visual adj1 loss)).ti,ab. or parkinson disease/ or parkinson*.ti,ab. or exp autism spectrum disorder/ or autis*.ti,ab.)

21 (anemia/ or an?emia*.ti,ab.) and (obesity/ or (obesity or obese).ti,ab. or prostatic hyperplasia/ or (prostatic adj1 (hyperplasia or hypertrophy)).ti,ab. or tuberculosis/ or tuberculosis.ti,ab. or exp chronic hepatitis/ or chronic hepatitis.ti,ab. or substance-related disorders/ or (((substance or drug or marijuana or cocaine or amphetamine) adj2 abuse) or "substance abuse" or addiction?).ti,ab. or inflammatory bowel diseases/ or (inflammatory bowel disease? or ulcerative colitis or crohn or ibd).ti,ab. or deafness/ or deaf*.ti,ab. or blindness/ or (blind* or (visual adj1 loss)).ti,ab. or parkinson disease/ or parkinson*.ti,ab. or exp autism spectrum disorder/ or autis*.ti,ab.)

22 (obesity/ or (obesity or obese).ti,ab.) and (prostatic hyperplasia/ or (prostatic adj1 (hyperplasia or hypertrophy)).ti,ab. or tuberculosis/ or tuberculosis.ti,ab. or exp chronic hepatitis/ or chronic hepatitis.ti,ab. or substance-related disorders/ or (((substance or drug or marijuana or cocaine or amphetamine) adj2 abuse) or "substance abuse" or addiction?).ti,ab. or inflammatory bowel diseases/ or (inflammatory bowel disease? or ulcerative colitis or crohn or ibd).ti,ab. or deafness/ or deaf*.ti,ab. or blindness/ or (blind* or (visual adj1 loss)).ti,ab. or parkinson disease/ or parkinson*.ti,ab. or exp autism spectrum disorder/ or autis*.ti,ab.)

23 (prostatic hyperplasia/ or (prostatic adj1 (hyperplasia or hypertrophy)).ti,ab.) and (tuberculosis/ or tuberculosis.ti,ab. or exp chronic hepatitis/ or chronic hepatitis.ti,ab. or substance-related disorders/ or (((substance or drug or marijuana or cocaine or amphetamine) adj2 abuse) or "substance abuse" or addiction?).ti,ab. or inflammatory bowel diseases/ or (inflammatory bowel disease? or ulcerative colitis or crohn or ibd).ti,ab. or deafness/ or deaf*.ti,ab. or blindness/ or (blind* or (visual adj1 loss)).ti,ab. or parkinson disease/ or parkinson*.ti,ab. or exp autism spectrum disorder/ or autis*.ti,ab.)

24 (tuberculosis/ or tuberculosis.ti,ab.) and (exp chronic hepatitis/ or chronic hepatitis.ti,ab. or substance-related disorders/ or (((substance or drug or marijuana or cocaine or amphetamine) adj2 abuse) or "substance abuse" or addiction?).ti,ab. or inflammatory bowel diseases/ or (inflammatory bowel disease? or ulcerative colitis or crohn or ibd).ti,ab. or deafness/ or deaf*.ti,ab. or blindness/ or (blind* or (visual adj1 loss)).ti,ab. or parkinson disease/ or parkinson*.ti,ab. or exp autism spectrum disorder/ or autis*.ti,ab.)

25 (exp chronic hepatitis/ or chronic hepatitis.ti,ab.) and (substance-related disorders/ or (((substance or drug or marijuana or cocaine or amphetamine) adj2 abuse) or "substance abuse" or addiction?).ti,ab. or inflammatory bowel diseases/ or (inflammatory bowel disease? or ulcerative colitis or crohn or ibd).ti,ab. or deafness/ or deaf*.ti,ab. or blindness/ or (blind* or (visual adj1 loss)).ti,ab. or parkinson disease/ or parkinson*.ti,ab. or exp autism spectrum disorder/ or autis*.ti,ab.)

26 (substance-related disorders/ or (((substance or drug or marijuana or cocaine or amphetamine) adj2 abuse) or "substance abuse" or addiction?).ti,ab.) and (inflammatory bowel diseases/ or (inflammatory bowel disease? or ulcerative colitis or crohn or ibd).ti,ab. or deafness/ or deaf*.ti,ab. or blindness/ or (blind* or (visual adj1 loss)).ti,ab. or parkinson disease/ or parkinson*.ti,ab. or exp autism spectrum disorder/ or autis*.ti,ab.)

27 (inflammatory bowel diseases/ or (inflammatory bowel disease? or ulcerative colitis or crohn or ibd).ti,ab.) and (deafness/ or deaf*.ti,ab. or blindness/ or (blind* or (visual adj1 loss)).ti,ab. or parkinson disease/ or parkinson*.ti,ab. or exp autism spectrum disorder/ or autis*.ti,ab.)

28 (deafness/ or deaf*.ti,ab.) and (blindness/ or (blind* or (visual adj1 loss)).ti,ab. or parkinson disease/ or parkinson*.ti,ab. or exp autism spectrum disorder/ or autis*.ti,ab.)

29 (blindness/ or (blind* or (visual adj1 loss)).ti,ab.) and (parkinson disease/ or parkinson*.ti,ab. or exp autism spectrum disorder/ or autis*.ti,ab.)

30 (parkinson disease/ or parkinson*.ti,ab.) and (exp autism spectrum disorder/ or autis*.ti,ab.)

31 exp comorbidity/

32 (((condition* or diagnos* or disease* or illness* or health problem* or patholog* or disorder* or syndrome*) adj1 (associated or coexisting or co-existing or comorbid or co-morbid or concurrent or co-occuring or cooccuring or multiple)) or associated morbidit*).ti,ab.

33 (comorbidit* or co-morbidit* or multidisease* or multi-disease* or multimorbidit* or multi-morbidit* or multipatholog* or multi-patholog* or pluripatholog* or polypatholog* or poly-pathology*).ab,ti.

34 1 or 2 or 3 or 4 or 5 or 6 or 7 or 8 or 9 or 10 or 11 or 12 or 13 or 14 or 15 or 16 or 17 or 18 or 19 or 20 or 21 or 22 or 23 or 24 or 25 or 26 or 27 or 28 or 29 or 30

35 31 or 32 or 33

36 exp health care costs/ or exp health expenditures/

37 cost*.mp. or ((health care or health-care or healthcare or medical or hospital) adj3 (expenditure or expense or spend or pay*)).ti,ab.

38 (((resource or "health service") adj3 (utilisation or utilization)) or ((cost or financial or economic) and (burden or impact or consequence)) or out of pocket or out-of-pocket or oop or absenteeism or (productivity adj3 (loss or lost)) or economic model*).ab,ti.

39 35 or 36 or 37

40 34 and 35 and 39

41 limit 40 to yr="2010 -Current"

Business Source Complete

To perform this search in the database, use the advanced search and enter each syntax in two search boxes -> one with the field Title and one with the field Abstract + combine both with OR


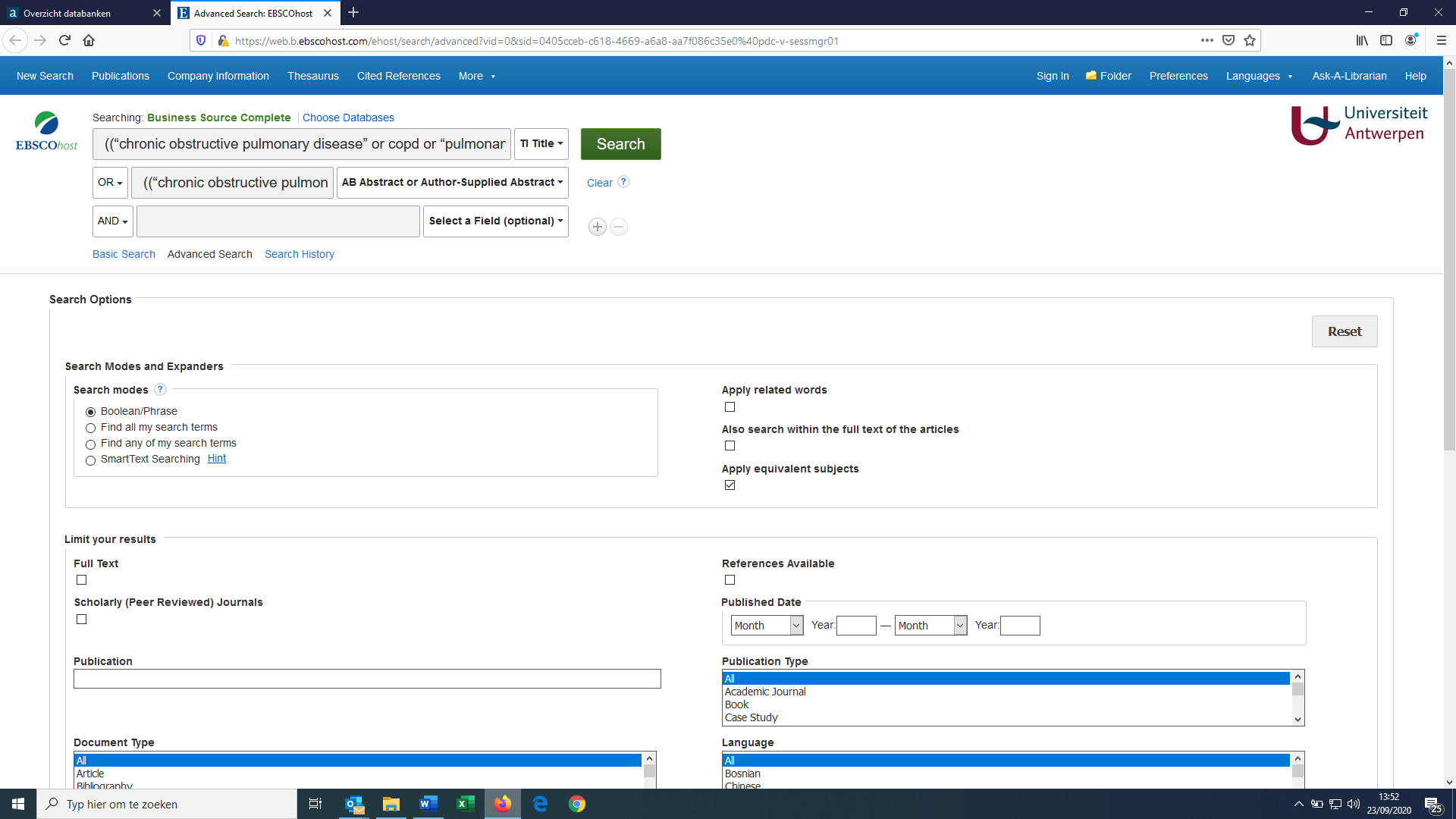


1. ((“chronic obstructive pulmonary disease” or copd or “pulmonary disease?” or “pulmonary disorder?”) and (diabet* or hypertens* or "high blood pressure*" or neoplasm? or cancer? or ((cardiac or cardiovascular or coronary) N1 (disease? or disorder? or failure)) or “myocardial infarct*” or “cardiovascular strok*” or “heart failure” or “cardiac failure” or “myocardial failure” or asthma* or ((mental or anxiety or mood or psychological) N1 (disease? or disorder?)) or “sleep disorder” or depression? or osteoporosis or dyslipid?emia* or hyperlipid?emia* or hypercholesterolemia* or hypertriglyceridemia* or “thyroid disease?” or “thyroid disorder?” or hyperthyroid* or hypothyroid* or “joint disease*” or “rheumatoid arthritis” or osteoarthritis or “kidney disease?” or “kidney disorder?” or epilep* or seizure? or hiv or “acquired immun* deficiency syndrome?” or aids or “liver disease?” or “liver disorder?” or stroke* or dementia or “cardiac arrhythmia*” or an?emia* or obesity or obese or “prostatic hyperplasia” or “prostatic hypertrophy” or tuberculosis or “chronic hepatitis” or “substance-related disorder?” or “substance abuse” or “drug abuse” or “marijuana abuse” or “cocaine abuse” or “amphetamine abuse” or addiction? or “inflammatory bowel disease?” or “ulcerative colitis” or crohn or ibd or deaf* or blind* or “visual loss” or parkinson* or autis*))
2. ((diabet*) AND (hypertens* or "high blood pressure*" or neoplasm? or cancer? or ((cardiac or cardiovascular or coronary) N1 (disease? or disorder? or failure)) or “myocardial infarct*” or “cardiovascular strok*” or “heart failure” or “cardiac failure” or “myocardial failure” or asthma* or ((mental or anxiety or mood or psychological) N1 (disease? or disorder?)) or “sleep disorder” or depression? or osteoporosis or dyslipid?emia* or hyperlipid?emia* or hypercholesterolemia* or hypertriglyceridemia* or “thyroid disease?” or “thyroid disorder?” or hyperthyroid* or hypothyroid* or “joint disease*” or “rheumatoid arthritis” or osteoarthritis or “kidney disease?” or “kidney disorder?” or epilep* or seizure? or hiv or “acquired immun* deficiency syndrome?” or aids or “liver disease?” or “liver disorder?” or stroke* or dementia or “cardiac arrhythmia*” or an?emia* or obesity or obese or “prostatic hyperplasia” or “prostatic hypertrophy” or tuberculosis or “chronic hepatitis” or “substance-related disorder?” or “substance abuse” or “drug abuse” or “marijuana abuse” or “cocaine abuse” or “amphetamine abuse” or addiction? or “inflammatory bowel disease?” or “ulcerative colitis” or crohn or ibd or deaf* or blind* or “visual loss” or parkinson* or autis*))
3. ((hypertens* or "high blood pressure*") AND (neoplasm? or cancer? or ((cardiac or cardiovascular or coronary) N1 (disease? or disorder? or failure)) or “myocardial infarct*” or “cardiovascular strok*” or “heart failure” or “cardiac failure” or “myocardial failure” or asthma* or ((mental or anxiety or mood or psychological) N1 (disease? or disorder?)) or “sleep disorder” or depression? or osteoporosis or dyslipid?emia* or hyperlipid?emia* or hypercholesterolemia* or hypertriglyceridemia* or “thyroid disease?” or “thyroid disorder?” or hyperthyroid* or hypothyroid* or “joint disease*” or “rheumatoid arthritis” or osteoarthritis or “kidney disease?” or “kidney disorder?” or epilep* or seizure? or hiv or “acquired immun* deficiency syndrome?” or aids or “liver disease?” or “liver disorder?” or stroke* or dementia or “cardiac arrhythmia*” or an?emia* or obesity or obese or “prostatic hyperplasia” or “prostatic hypertrophy” or tuberculosis or “chronic hepatitis” or “substance-related disorder?” or “substance abuse” or “drug abuse” or “marijuana abuse” or “cocaine abuse” or “amphetamine abuse” or addiction? or “inflammatory bowel disease?” or “ulcerative colitis” or crohn or ibd or deaf* or blind* or “visual loss” or parkinson* or autis*))
4. ((neoplasm? or cancer?) AND (((cardiac or cardiovascular or coronary) N1 (disease? or disorder? or failure)) or “myocardial infarct*” or “cardiovascular strok*” or “heart failure” or “cardiac failure” or “myocardial failure” or asthma* or ((mental or anxiety or mood or psychological) N1 (disease? or disorder?)) or “sleep disorder” or depression? or osteoporosis or dyslipid?emia* or hyperlipid?emia* or hypercholesterolemia* or hypertriglyceridemia* or “thyroid disease?” or “thyroid disorder?” or hyperthyroid* or hypothyroid* or “joint disease*” or “rheumatoid arthritis” or osteoarthritis or “kidney disease?” or “kidney disorder?” or epilep* or seizure? or hiv or “acquired immun* deficiency syndrome?” or aids or “liver disease?” or “liver disorder?” or stroke* or dementia or “cardiac arrhythmia*” or an?emia* or obesity or obese or “prostatic hyperplasia” or “prostatic hypertrophy” or tuberculosis or “chronic hepatitis” or “substance-related disorder?” or “substance abuse” or “drug abuse” or “marijuana abuse” or “cocaine abuse” or “amphetamine abuse” or addiction? or “inflammatory bowel disease?” or “ulcerative colitis” or crohn or ibd or deaf* or blind* or “visual loss” or parkinson* or autis*))
5. (((cardiac or cardiovascular or coronary) N1 (disease? or disorder? or failure)) AND (“myocardial infarct*” or “cardiovascular strok*” or “heart failure” or “cardiac failure” or “myocardial failure” or asthma* or ((mental or anxiety or mood or psychological) N1 (disease? or disorder?)) or “sleep disorder” or depression? or osteoporosis or dyslipid?emia* or hyperlipid?emia* or hypercholesterolemia* or hypertriglyceridemia* or “thyroid disease?” or “thyroid disorder?” or hyperthyroid* or hypothyroid* or “joint disease*” or “rheumatoid arthritis” or osteoarthritis or “kidney disease?” or “kidney disorder?” or epilep* or seizure? or hiv or “acquired immun* deficiency syndrome?” or aids or “liver disease?” or “liver disorder?” or stroke* or dementia or “cardiac arrhythmia*” or an?emia* or obesity or obese or “prostatic hyperplasia” or “prostatic hypertrophy” or tuberculosis or “chronic hepatitis” or “substance-related disorder?” or “substance abuse” or “drug abuse” or “marijuana abuse” or “cocaine abuse” or “amphetamine abuse” or addiction? or “inflammatory bowel disease?” or “ulcerative colitis” or crohn or ibd or deaf* or blind* or “visual loss” or parkinson* or autis*))
6. ((“myocardial infarct*” or “cardiovascular strok*”) and (“heart failure” or “cardiac failure” or “myocardial failure” or asthma* or ((mental or anxiety or mood or psychological) N1 (disease? or disorder?)) or “sleep disorder” or depression? or osteoporosis or dyslipid?emia* or hyperlipid?emia* or hypercholesterolemia* or hypertriglyceridemia* or “thyroid disease?” or “thyroid disorder?” or hyperthyroid* or hypothyroid* or “joint disease*” or “rheumatoid arthritis” or osteoarthritis or “kidney disease?” or “kidney disorder?” or epilep* or seizure? or hiv or “acquired immun* deficiency syndrome?” or aids or “liver disease?” or “liver disorder?” or stroke* or dementia or “cardiac arrhythmia*” or an?emia* or obesity or obese or “prostatic hyperplasia” or “prostatic hypertrophy” or tuberculosis or “chronic hepatitis” or “substance-related disorder?” or “substance abuse” or “drug abuse” or “marijuana abuse” or “cocaine abuse” or “amphetamine abuse” or addiction? or “inflammatory bowel disease?” or “ulcerative colitis” or crohn or ibd or deaf* or blind* or “visual loss” or parkinson* or autis*))
7. ((“heart failure” or “cardiac failure” or “myocardial failure”) and (asthma* or ((mental or anxiety or mood or psychological) N1 (disease? or disorder?)) or “sleep disorder” or depression? or osteoporosis or dyslipid?emia* or hyperlipid?emia* or hypercholesterolemia* or hypertriglyceridemia* or “thyroid disease?” or “thyroid disorder?” or hyperthyroid* or hypothyroid* or “joint disease*” or “rheumatoid arthritis” or osteoarthritis or “kidney disease?” or “kidney disorder?” or epilep* or seizure? or hiv or “acquired immun* deficiency syndrome?” or aids or “liver disease?” or “liver disorder?” or stroke* or dementia or “cardiac arrhythmia*” or an?emia* or obesity or obese or “prostatic hyperplasia” or “prostatic hypertrophy” or tuberculosis or “chronic hepatitis” or “substance-related disorder?” or “substance abuse” or “drug abuse” or “marijuana abuse” or “cocaine abuse” or “amphetamine abuse” or addiction? or “inflammatory bowel disease?” or “ulcerative colitis” or crohn or ibd or deaf* or blind* or “visual loss” or parkinson* or autis*))
8. ((asthma*) AND (((mental or anxiety or mood or psychological) N1 (disease? or disorder?)) or “sleep disorder” or depression? or osteoporosis or dyslipid?emia* or hyperlipid?emia* or hypercholesterolemia* or hypertriglyceridemia* or “thyroid disease?” or “thyroid disorder?” or hyperthyroid* or hypothyroid* or “joint disease*” or “rheumatoid arthritis” or osteoarthritis or “kidney disease?” or “kidney disorder?” or epilep* or seizure? or hiv or “acquired immun* deficiency syndrome?” or aids or “liver disease?” or “liver disorder?” or stroke* or dementia or “cardiac arrhythmia*” or an?emia* or obesity or obese or “prostatic hyperplasia” or “prostatic hypertrophy” or tuberculosis or “chronic hepatitis” or “substance-related disorder?” or “substance abuse” or “drug abuse” or “marijuana abuse” or “cocaine abuse” or “amphetamine abuse” or addiction? or “inflammatory bowel disease?” or “ulcerative colitis” or crohn or ibd or deaf* or blind* or “visual loss” or parkinson* or autis*))
9. ((((mental or anxiety or mood or psychological) N1 (disease? or disorder?)) or “sleep disorder” or depression?) and (osteoporosis or dyslipid?emia* or hyperlipid?emia* or hypercholesterolemia* or hypertriglyceridemia* or “thyroid disease?” or “thyroid disorder?” or hyperthyroid* or hypothyroid* or “joint disease*” or “rheumatoid arthritis” or osteoarthritis or “kidney disease?” or “kidney disorder?” or epilep* or seizure? or hiv or “acquired immun* deficiency syndrome?” or aids or “liver disease?” or “liver disorder?” or stroke* or dementia or “cardiac arrhythmia*” or an?emia* or obesity or obese or “prostatic hyperplasia” or “prostatic hypertrophy” or tuberculosis or “chronic hepatitis” or “substance-related disorder?” or “substance abuse” or “drug abuse” or “marijuana abuse” or “cocaine abuse” or “amphetamine abuse” or addiction? or “inflammatory bowel disease?” or “ulcerative colitis” or crohn or ibd or deaf* or blind* or “visual loss” or parkinson* or autis*))
10. ((osteoporosis) and (dyslipid?emia* or hyperlipid?emia* or hypercholesterolemia* or hypertriglyceridemia* or “thyroid disease?” or “thyroid disorder?” or hyperthyroid* or hypothyroid* or “joint disease*” or “rheumatoid arthritis” or osteoarthritis or “kidney disease?” or “kidney disorder?” or epilep* or seizure? or hiv or “acquired immun* deficiency syndrome?” or aids or “liver disease?” or “liver disorder?” or stroke* or dementia or “cardiac arrhythmia*” or an?emia* or obesity or obese or “prostatic hyperplasia” or “prostatic hypertrophy” or tuberculosis or “chronic hepatitis” or “substance-related disorder?” or “substance abuse” or “drug abuse” or “marijuana abuse” or “cocaine abuse” or “amphetamine abuse” or addiction? or “inflammatory bowel disease?” or “ulcerative colitis” or crohn or ibd or deaf* or blind* or “visual loss” or parkinson* or autis*))
11. ((dyslipid?emia* or hyperlipid?emia* or hypercholesterolemia* or hypertriglyceridemia*) and (“thyroid disease?” or “thyroid disorder?” or hyperthyroid* or hypothyroid* or “joint disease*” or “rheumatoid arthritis” or osteoarthritis or “kidney disease?” or “kidney disorder?” or epilep* or seizure? or hiv or “acquired immun* deficiency syndrome?” or aids or “liver disease?” or “liver disorder?” or stroke* or dementia or “cardiac arrhythmia*” or an?emia* or obesity or obese or “prostatic hyperplasia” or “prostatic hypertrophy” or tuberculosis or “chronic hepatitis” or “substance-related disorder?” or “substance abuse” or “drug abuse” or “marijuana abuse” or “cocaine abuse” or “amphetamine abuse” or addiction? or “inflammatory bowel disease?” or “ulcerative colitis” or crohn or ibd or deaf* or blind* or “visual loss” or parkinson* or autis*))
12. ((“thyroid disease?” or “thyroid disorder?” or hyperthyroid* or hypothyroid*) and (“joint disease*” or “rheumatoid arthritis” or osteoarthritis or “kidney disease?” or “kidney disorder?” or epilep* or seizure? or hiv or “acquired immun* deficiency syndrome?” or aids or “liver disease?” or “liver disorder?” or stroke* or dementia or “cardiac arrhythmia*” or an?emia* or obesity or obese or “prostatic hyperplasia” or “prostatic hypertrophy” or tuberculosis or “chronic hepatitis” or “substance-related disorder?” or “substance abuse” or “drug abuse” or “marijuana abuse” or “cocaine abuse” or “amphetamine abuse” or addiction? or “inflammatory bowel disease?” or “ulcerative colitis” or crohn or ibd or deaf* or blind* or “visual loss” or parkinson* or autis*))
13. ((“joint disease*” or “rheumatoid arthritis” or osteoarthritis) and (“kidney disease?” or “kidney disorder?” or epilep* or seizure? or hiv or “acquired immun* deficiency syndrome?” or aids or “liver disease?” or “liver disorder?” or stroke* or dementia or “cardiac arrhythmia*” or an?emia* or obesity or obese or “prostatic hyperplasia” or “prostatic hypertrophy” or tuberculosis or “chronic hepatitis” or “substance-related disorder?” or “substance abuse” or “drug abuse” or “marijuana abuse” or “cocaine abuse” or “amphetamine abuse” or addiction? or “inflammatory bowel disease?” or “ulcerative colitis” or crohn or ibd or deaf* or blind* or “visual loss” or parkinson* or autis*))
14. ((“kidney disease?” or “kidney disorder?”) and (epilep* or seizure? or hiv or “acquired immun* deficiency syndrome?” or aids or “liver disease?” or “liver disorder?” or stroke* or dementia or “cardiac arrhythmia*” or an?emia* or obesity or obese or “prostatic hyperplasia” or “prostatic hypertrophy” or tuberculosis or “chronic hepatitis” or “substance-related disorder?” or “substance abuse” or “drug abuse” or “marijuana abuse” or “cocaine abuse” or “amphetamine abuse” or addiction? or “inflammatory bowel disease?” or “ulcerative colitis” or crohn or ibd or deaf* or blind* or “visual loss” or parkinson* or autis*))
15. ((epilep* or seizure?) and (hiv or “acquired immun* deficiency syndrome?” or aids or “liver disease?” or “liver disorder?” or stroke* or dementia or “cardiac arrhythmia*” or an?emia* or obesity or obese or “prostatic hyperplasia” or “prostatic hypertrophy” or tuberculosis or “chronic hepatitis” or “substance-related disorder?” or “substance abuse” or “drug abuse” or “marijuana abuse” or “cocaine abuse” or “amphetamine abuse” or addiction? or “inflammatory bowel disease?” or “ulcerative colitis” or crohn or ibd or deaf* or blind* or “visual loss” or parkinson* or autis*))
16. ((hiv or “acquired immun* deficiency syndrome?” or aids) and (“liver disease?” or “liver disorder?” or stroke* or dementia or “cardiac arrhythmia*” or an?emia* or obesity or obese or “prostatic hyperplasia” or “prostatic hypertrophy” or tuberculosis or “chronic hepatitis” or “substance-related disorder?” or “substance abuse” or “drug abuse” or “marijuana abuse” or “cocaine abuse” or “amphetamine abuse” or addiction? or “inflammatory bowel disease?” or “ulcerative colitis” or crohn or ibd or deaf* or blind* or “visual loss” or parkinson* or autis*))
17. ((“liver disease?” or “liver disorder?”) and (stroke* or dementia or “cardiac arrhythmia*” or an?emia* or obesity or obese or “prostatic hyperplasia” or “prostatic hypertrophy” or tuberculosis or “chronic hepatitis” or “substance-related disorder?” or “substance abuse” or “drug abuse” or “marijuana abuse” or “cocaine abuse” or “amphetamine abuse” or addiction? or “inflammatory bowel disease?” or “ulcerative colitis” or crohn or ibd or deaf* or blind* or “visual loss” or parkinson* or autis*))
18. ((stroke*) and (dementia or “cardiac arrhythmia*” or an?emia* or obesity or obese or “prostatic hyperplasia” or “prostatic hypertrophy” or tuberculosis or “chronic hepatitis” or “substance-related disorder?” or “substance abuse” or “drug abuse” or “marijuana abuse” or “cocaine abuse” or “amphetamine abuse” or addiction? or “inflammatory bowel disease?” or “ulcerative colitis” or crohn or ibd or deaf* or blind* or “visual loss” or parkinson* or autis*))
19. ((dementia) and (“cardiac arrhythmia*” or an?emia* or obesity or obese or “prostatic hyperplasia” or “prostatic hypertrophy” or tuberculosis or “chronic hepatitis” or “substance-related disorder?” or “substance abuse” or “drug abuse” or “marijuana abuse” or “cocaine abuse” or “amphetamine abuse” or addiction? or “inflammatory bowel disease?” or “ulcerative colitis” or crohn or ibd or deaf* or blind* or “visual loss” or parkinson* or autis*))
20. ((“cardiac arrhythmia*”) and (an?emia* or obesity or obese or “prostatic hyperplasia” or “prostatic hypertrophy” or tuberculosis or “chronic hepatitis” or “substance-related disorder?” or “substance abuse” or “drug abuse” or “marijuana abuse” or “cocaine abuse” or “amphetamine abuse” or addiction? or “inflammatory bowel disease?” or “ulcerative colitis” or crohn or ibd or deaf* or blind* or “visual loss” or parkinson* or autis*))
21. ((an?emia*) and (obesity or obese or “prostatic hyperplasia” or “prostatic hypertrophy” or tuberculosis or “chronic hepatitis” or “substance-related disorder?” or “substance abuse” or “drug abuse” or “marijuana abuse” or “cocaine abuse” or “amphetamine abuse” or addiction? or “inflammatory bowel disease?” or “ulcerative colitis” or crohn or ibd or deaf* or blind* or “visual loss” or parkinson* or autis*))
22. ((obesity or obese) and (“prostatic hyperplasia” or “prostatic hypertrophy” or tuberculosis or “chronic hepatitis” or “substance-related disorder?” or “substance abuse” or “drug abuse” or “marijuana abuse” or “cocaine abuse” or “amphetamine abuse” or addiction? or “inflammatory bowel disease?” or “ulcerative colitis” or crohn or ibd or deaf* or blind* or “visual loss” or parkinson* or autis*))
23. ((“prostatic hyperplasia” or “prostatic hypertrophy”) and (tuberculosis or “chronic hepatitis” or “substance-related disorder?” or “substance abuse” or “drug abuse” or “marijuana abuse” or “cocaine abuse” or “amphetamine abuse” or addiction? or “inflammatory bowel disease?” or “ulcerative colitis” or crohn or ibd or deaf* or blind* or “visual loss” or parkinson* or autis*))
24. ((tuberculosis) and (“chronic hepatitis” or “substance-related disorder?” or “substance abuse” or “drug abuse” or “marijuana abuse” or “cocaine abuse” or “amphetamine abuse” or addiction? or “inflammatory bowel disease?” or “ulcerative colitis” or crohn or ibd or deaf* or blind* or “visual loss” or parkinson* or autis*))
25. ((“chronic hepatitis”) and (“substance-related disorder?” or “substance abuse” or “drug abuse” or “marijuana abuse” or “cocaine abuse” or “amphetamine abuse” or addiction? or “inflammatory bowel disease?” or “ulcerative colitis” or crohn or ibd or deaf* or blind* or “visual loss” or parkinson* or autis*))
26. ((“substance-related disorder?” or “substance abuse” or “drug abuse” or “marijuana abuse” or “cocaine abuse” or “amphetamine abuse” or addiction?) and (“inflammatory bowel disease?” or “ulcerative colitis” or crohn or ibd or deaf* or blind* or “visual loss” or parkinson* or autis*))
27. ((“inflammatory bowel disease?” or “ulcerative colitis” or crohn or ibd) and (deaf* or blind* or “visual loss” or parkinson* or autis*))
28. ((deaf*) and (blind* or “visual loss” or parkinson* or autis*))
29. ((blind* or “visual loss”) and (parkinson* or autis*))
30. (parkinson* and autis*)
31. ((condition* or diagnos* or disease* or illness* or “health problem*” or patholog* or disorder* or syndrome*) N1 (associated or coexisting or co-existing or comorbid or co-morbid or concurrent or co-occuring or cooccuring or multiple) or “associated morbidit*”)
32. (comorbidit* or co-morbidit* or multidisease* or multi-disease* or multimorbidit* or multi-morbidit* or multipatholog* or multi-patholog* or pluripatholog* or polypatholog* or poly-pathology*)
33. 1 or 2 or 3 or 4 or 5 or 6 or 7 or 8 or 9 or 10 or 11 or 12 or 13 or 14 or 15 or 16 or 17 or 18 or 19 or 20 or 21 or 22 or 23 or 24 or 25 or 26 or 27 or 28 or 29 or 30
34. 31 or 32
35. (cost* or ((health or health-care or healthcare or medical) N3 expenditure*) or expense* or “hospital resource*”)
36. (((resource* or "health service") N3 ("use" or utilisation or utilization)) or ((financial or economic) N3 (burden or impact or consequence)) or spending or “out of pocket” or out-of-pocket or oop or insurance or absenteeism or (productivity N3 (loss or lost)) or "economic modelling")
37. 35 or 36
38. 33 and 34 and 37
39. limit 38 to yr="2010 -Current"

Scopus

1. TITLE-ABS-KEY((“chronic obstructive pulmonary disease” or copd or “pulmonary disease?” or “pulmonary disorder?”) and (diabet* or hypertens* or "high blood pressure*" or neoplasm? or cancer? or ((cardiac or cardiovascular or coronary) W/1 (disease? or disorder? or failure)) or “myocardial infarct*” or “cardiovascular strok*” or “heart failure” or “cardiac failure” or “myocardial failure” or asthma* or ((mental or anxiety or mood or psychological) W/1 (disease? or disorder?)) or “sleep disorder” or depression? or osteoporosis or dyslipid?emia* or hyperlipid?emia* or hypercholesterolemia* or hypertriglyceridemia* or “thyroid disease?” or “thyroid disorder?” or hyperthyroid* or hypothyroid* or “joint disease*” or “rheumatoid arthritis” or osteoarthritis or “kidney disease?” or “kidney disorder?” or epilep* or seizure? or hiv or “acquired immun* deficiency syndrome?” or aids or “liver disease?” or “liver disorder?” or stroke* or dementia or “cardiac arrhythmia*” or an?emia* or obesity or obese or “prostatic hyperplasia” or “prostatic hypertrophy” or tuberculosis or “chronic hepatitis” or “substance-related disorder?” or “substance abuse” or “drug abuse” or “marijuana abuse” or “cocaine abuse” or “amphetamine abuse” or addiction? or “inflammatory bowel disease?” or “ulcerative colitis” or crohn or ibd or deaf* or blind* or “visual loss” or parkinson* or autis*))
2. TITLE-ABS-KEY((diabet*) AND (hypertens* or "high blood pressure*" or neoplasm? or cancer? or ((cardiac or cardiovascular or coronary) W/1 (disease? or disorder? or failure)) or “myocardial infarct*” or “cardiovascular strok*” or “heart failure” or “cardiac failure” or “myocardial failure” or asthma* or ((mental or anxiety or mood or psychological) W/1 (disease? or disorder?)) or “sleep disorder” or depression? or osteoporosis or dyslipid?emia* or hyperlipid?emia* or hypercholesterolemia* or hypertriglyceridemia* or “thyroid disease?” or “thyroid disorder?” or hyperthyroid* or hypothyroid* or “joint disease*” or “rheumatoid arthritis” or osteoarthritis or “kidney disease?” or “kidney disorder?” or epilep* or seizure? or hiv or “acquired immun* deficiency syndrome?” or aids or “liver disease?” or “liver disorder?” or stroke* or dementia or “cardiac arrhythmia*” or an?emia* or obesity or obese or “prostatic hyperplasia” or “prostatic hypertrophy” or tuberculosis or “chronic hepatitis” or “substance-related disorder?” or “substance abuse” or “drug abuse” or “marijuana abuse” or “cocaine abuse” or “amphetamine abuse” or addiction? or “inflammatory bowel disease?” or “ulcerative colitis” or crohn or ibd or deaf* or blind* or “visual loss” or parkinson* or autis*))
3. TITLE-ABS-KEY((hypertens* or "high blood pressure*") AND (neoplasm? or cancer? or ((cardiac or cardiovascular or coronary) W/1 (disease? or disorder? or failure)) or “myocardial infarct*” or “cardiovascular strok*” or “heart failure” or “cardiac failure” or “myocardial failure” or asthma* or ((mental or anxiety or mood or psychological) W/1 (disease? or disorder?)) or “sleep disorder” or depression? or osteoporosis or dyslipid?emia* or hyperlipid?emia* or hypercholesterolemia* or hypertriglyceridemia* or “thyroid disease?” or “thyroid disorder?” or hyperthyroid* or hypothyroid* or “joint disease*” or “rheumatoid arthritis” or osteoarthritis or “kidney disease?” or “kidney disorder?” or epilep* or seizure? or hiv or “acquired immun* deficiency syndrome?” or aids or “liver disease?” or “liver disorder?” or stroke* or dementia or “cardiac arrhythmia*” or an?emia* or obesity or obese or “prostatic hyperplasia” or “prostatic hypertrophy” or tuberculosis or “chronic hepatitis” or “substance-related disorder?” or “substance abuse” or “drug abuse” or “marijuana abuse” or “cocaine abuse” or “amphetamine abuse” or addiction? or “inflammatory bowel disease?” or “ulcerative colitis” or crohn or ibd or deaf* or blind* or “visual loss” or parkinson* or autis*))
4. TITLE-ABS-KEY((neoplasm? or cancer?) AND (((cardiac or cardiovascular or coronary) W/1 (disease? or disorder? or failure)) or “myocardial infarct*” or “cardiovascular strok*” or “heart failure” or “cardiac failure” or “myocardial failure” or asthma* or ((mental or anxiety or mood or psychological) W/1 (disease? or disorder?)) or “sleep disorder” or depression? or osteoporosis or dyslipid?emia* or hyperlipid?emia* or hypercholesterolemia* or hypertriglyceridemia* or “thyroid disease?” or “thyroid disorder?” or hyperthyroid* or hypothyroid* or “joint disease*” or “rheumatoid arthritis” or osteoarthritis or “kidney disease?” or “kidney disorder?” or epilep* or seizure? or hiv or “acquired immun* deficiency syndrome?” or aids or “liver disease?” or “liver disorder?” or stroke* or dementia or “cardiac arrhythmia*” or an?emia* or obesity or obese or “prostatic hyperplasia” or “prostatic hypertrophy” or tuberculosis or “chronic hepatitis” or “substance-related disorder?” or “substance abuse” or “drug abuse” or “marijuana abuse” or “cocaine abuse” or “amphetamine abuse” or addiction? or “inflammatory bowel disease?” or “ulcerative colitis” or crohn or ibd or deaf* or blind* or “visual loss” or parkinson* or autis*))
5. TITLE-ABS-KEY(((cardiac or cardiovascular or coronary) W/1 (disease? or disorder? or failure)) AND (“myocardial infarct*” or “cardiovascular strok*” or “heart failure” or “cardiac failure” or “myocardial failure” or asthma* or ((mental or anxiety or mood or psychological) W/1 (disease? or disorder?)) or “sleep disorder” or depression? or osteoporosis or dyslipid?emia* or hyperlipid?emia* or hypercholesterolemia* or hypertriglyceridemia* or “thyroid disease?” or “thyroid disorder?” or hyperthyroid* or hypothyroid* or “joint disease*” or “rheumatoid arthritis” or osteoarthritis or “kidney disease?” or “kidney disorder?” or epilep* or seizure? or hiv or “acquired immun* deficiency syndrome?” or aids or “liver disease?” or “liver disorder?” or stroke* or dementia or “cardiac arrhythmia*” or an?emia* or obesity or obese or “prostatic hyperplasia” or “prostatic hypertrophy” or tuberculosis or “chronic hepatitis” or “substance-related disorder?” or “substance abuse” or “drug abuse” or “marijuana abuse” or “cocaine abuse” or “amphetamine abuse” or addiction? or “inflammatory bowel disease?” or “ulcerative colitis” or crohn or ibd or deaf* or blind* or “visual loss” or parkinson* or autis*))
6. TITLE-ABS-KEY((“myocardial infarct*” or “cardiovascular strok*”) and (“heart failure” or “cardiac failure” or “myocardial failure” or asthma* or ((mental or anxiety or mood or psychological) W/1 (disease? or disorder?)) or “sleep disorder” or depression? or osteoporosis or dyslipid?emia* or hyperlipid?emia* or hypercholesterolemia* or hypertriglyceridemia* or “thyroid disease?” or “thyroid disorder?” or hyperthyroid* or hypothyroid* or “joint disease*” or “rheumatoid arthritis” or osteoarthritis or “kidney disease?” or “kidney disorder?” or epilep* or seizure? or hiv or “acquired immun* deficiency syndrome?” or aids or “liver disease?” or “liver disorder?” or stroke* or dementia or “cardiac arrhythmia*” or an?emia* or obesity or obese or “prostatic hyperplasia” or “prostatic hypertrophy” or tuberculosis or “chronic hepatitis” or “substance-related disorder?” or “substance abuse” or “drug abuse” or “marijuana abuse” or “cocaine abuse” or “amphetamine abuse” or addiction? or “inflammatory bowel disease?” or “ulcerative colitis” or crohn or ibd or deaf* or blind* or “visual loss” or parkinson* or autis*))
7. TITLE-ABS-KEY((“heart failure” or “cardiac failure” or “myocardial failure”) and (asthma* or ((mental or anxiety or mood or psychological) W/1 (disease? or disorder?)) or “sleep disorder” or depression? or osteoporosis or dyslipid?emia* or hyperlipid?emia* or hypercholesterolemia* or hypertriglyceridemia* or “thyroid disease?” or “thyroid disorder?” or hyperthyroid* or hypothyroid* or “joint disease*” or “rheumatoid arthritis” or osteoarthritis or “kidney disease?” or “kidney disorder?” or epilep* or seizure? or hiv or “acquired immun* deficiency syndrome?” or aids or “liver disease?” or “liver disorder?” or stroke* or dementia or “cardiac arrhythmia*” or an?emia* or obesity or obese or “prostatic hyperplasia” or “prostatic hypertrophy” or tuberculosis or “chronic hepatitis” or “substance-related disorder?” or “substance abuse” or “drug abuse” or “marijuana abuse” or “cocaine abuse” or “amphetamine abuse” or addiction? or “inflammatory bowel disease?” or “ulcerative colitis” or crohn or ibd or deaf* or blind* or “visual loss” or parkinson* or autis*))
8. TITLE-ABS-KEY((asthma*) AND (((mental or anxiety or mood or psychological) W/1 (disease? or disorder?)) or “sleep disorder” or depression? or osteoporosis or dyslipid?emia* or hyperlipid?emia* or hypercholesterolemia* or hypertriglyceridemia* or “thyroid disease?” or “thyroid disorder?” or hyperthyroid* or hypothyroid* or “joint disease*” or “rheumatoid arthritis” or osteoarthritis or “kidney disease?” or “kidney disorder?” or epilep* or seizure? or hiv or “acquired immun* deficiency syndrome?” or aids or “liver disease?” or “liver disorder?” or stroke* or dementia or “cardiac arrhythmia*” or an?emia* or obesity or obese or “prostatic hyperplasia” or “prostatic hypertrophy” or tuberculosis or “chronic hepatitis” or “substance-related disorder?” or “substance abuse” or “drug abuse” or “marijuana abuse” or “cocaine abuse” or “amphetamine abuse” or addiction? or “inflammatory bowel disease?” or “ulcerative colitis” or crohn or ibd or deaf* or blind* or “visual loss” or parkinson* or autis*))
9. TITLE-ABS-KEY((((mental or anxiety or mood or psychological) W/1 (disease? or disorder?)) or “sleep disorder” or depression?) and (osteoporosis or dyslipid?emia* or hyperlipid?emia* or hypercholesterolemia* or hypertriglyceridemia* or “thyroid disease?” or “thyroid disorder?” or hyperthyroid* or hypothyroid* or “joint disease*” or “rheumatoid arthritis” or osteoarthritis or “kidney disease?” or “kidney disorder?” or epilep* or seizure? or hiv or “acquired immun* deficiency syndrome?” or aids or “liver disease?” or “liver disorder?” or stroke* or dementia or “cardiac arrhythmia*” or an?emia* or obesity or obese or “prostatic hyperplasia” or “prostatic hypertrophy” or tuberculosis or “chronic hepatitis” or “substance-related disorder?” or “substance abuse” or “drug abuse” or “marijuana abuse” or “cocaine abuse” or “amphetamine abuse” or addiction? or “inflammatory bowel disease?” or “ulcerative colitis” or crohn or ibd or deaf* or blind* or “visual loss” or parkinson* or autis*))
10. TITLE-ABS-KEY((osteoporosis) and (dyslipid?emia* or hyperlipid?emia* or hypercholesterolemia* or hypertriglyceridemia* or “thyroid disease?” or “thyroid disorder?” or hyperthyroid* or hypothyroid* or “joint disease*” or “rheumatoid arthritis” or osteoarthritis or “kidney disease?” or “kidney disorder?” or epilep* or seizure? or hiv or “acquired immun* deficiency syndrome?” or aids or “liver disease?” or “liver disorder?” or stroke* or dementia or “cardiac arrhythmia*” or an?emia* or obesity or obese or “prostatic hyperplasia” or “prostatic hypertrophy” or tuberculosis or “chronic hepatitis” or “substance-related disorder?” or “substance abuse” or “drug abuse” or “marijuana abuse” or “cocaine abuse” or “amphetamine abuse” or addiction? or “inflammatory bowel disease?” or “ulcerative colitis” or crohn or ibd or deaf* or blind* or “visual loss” or parkinson* or autis*))
11. TITLE-ABS-KEY((dyslipid?emia* or hyperlipid?emia* or hypercholesterolemia* or hypertriglyceridemia*) and (“thyroid disease?” or “thyroid disorder?” or hyperthyroid* or hypothyroid* or “joint disease*” or “rheumatoid arthritis” or osteoarthritis or “kidney disease?” or “kidney disorder?” or epilep* or seizure? or hiv or “acquired immun* deficiency syndrome?” or aids or “liver disease?” or “liver disorder?” or stroke* or dementia or “cardiac arrhythmia*” or an?emia* or obesity or obese or “prostatic hyperplasia” or “prostatic hypertrophy” or tuberculosis or “chronic hepatitis” or “substance-related disorder?” or “substance abuse” or “drug abuse” or “marijuana abuse” or “cocaine abuse” or “amphetamine abuse” or addiction? or “inflammatory bowel disease?” or “ulcerative colitis” or crohn or ibd or deaf* or blind* or “visual loss” or parkinson* or autis*))
12. TITLE-ABS-KEY((“thyroid disease?” or “thyroid disorder?” or hyperthyroid* or hypothyroid*) and (“joint disease*” or “rheumatoid arthritis” or osteoarthritis or “kidney disease?” or “kidney disorder?” or epilep* or seizure? or hiv or “acquired immun* deficiency syndrome?” or aids or “liver disease?” or “liver disorder?” or stroke* or dementia or “cardiac arrhythmia*” or an?emia* or obesity or obese or “prostatic hyperplasia” or “prostatic hypertrophy” or tuberculosis or “chronic hepatitis” or “substance-related disorder?” or “substance abuse” or “drug abuse” or “marijuana abuse” or “cocaine abuse” or “amphetamine abuse” or addiction? or “inflammatory bowel disease?” or “ulcerative colitis” or crohn or ibd or deaf* or blind* or “visual loss” or parkinson* or autis*))
13. TITLE-ABS-KEY((“joint disease*” or “rheumatoid arthritis” or osteoarthritis) and (“kidney disease?” or “kidney disorder?” or epilep* or seizure? or hiv or “acquired immun* deficiency syndrome?” or aids or “liver disease?” or “liver disorder?” or stroke* or dementia or “cardiac arrhythmia*” or an?emia* or obesity or obese or “prostatic hyperplasia” or “prostatic hypertrophy” or tuberculosis or “chronic hepatitis” or “substance-related disorder?” or “substance abuse” or “drug abuse” or “marijuana abuse” or “cocaine abuse” or “amphetamine abuse” or addiction? or “inflammatory bowel disease?” or “ulcerative colitis” or crohn or ibd or deaf* or blind* or “visual loss” or parkinson* or autis*))
14. TITLE-ABS-KEY((“kidney disease?” or “kidney disorder?”) and (epilep* or seizure? or hiv or “acquired immun* deficiency syndrome?” or aids or “liver disease?” or “liver disorder?” or stroke* or dementia or “cardiac arrhythmia*” or an?emia* or obesity or obese or “prostatic hyperplasia” or “prostatic hypertrophy” or tuberculosis or “chronic hepatitis” or “substance-related disorder?” or “substance abuse” or “drug abuse” or “marijuana abuse” or “cocaine abuse” or “amphetamine abuse” or addiction? or “inflammatory bowel disease?” or “ulcerative colitis” or crohn or ibd or deaf* or blind* or “visual loss” or parkinson* or autis*))
15. TITLE-ABS-KEY((epilep* or seizure?) and (hiv or “acquired immun* deficiency syndrome?” or aids or “liver disease?” or “liver disorder?” or stroke* or dementia or “cardiac arrhythmia*” or an?emia* or obesity or obese or “prostatic hyperplasia” or “prostatic hypertrophy” or tuberculosis or “chronic hepatitis” or “substance-related disorder?” or “substance abuse” or “drug abuse” or “marijuana abuse” or “cocaine abuse” or “amphetamine abuse” or addiction? or “inflammatory bowel disease?” or “ulcerative colitis” or crohn or ibd or deaf* or blind* or “visual loss” or parkinson* or autis*))
16. TITLE-ABS-KEY((hiv or “acquired immun* deficiency syndrome?” or aids) and (“liver disease?” or “liver disorder?” or stroke* or dementia or “cardiac arrhythmia*” or an?emia* or obesity or obese or “prostatic hyperplasia” or “prostatic hypertrophy” or tuberculosis or “chronic hepatitis” or “substance-related disorder?” or “substance abuse” or “drug abuse” or “marijuana abuse” or “cocaine abuse” or “amphetamine abuse” or addiction? or “inflammatory bowel disease?” or “ulcerative colitis” or crohn or ibd or deaf* or blind* or “visual loss” or parkinson* or autis*))
17. TITLE-ABS-KEY((“liver disease?” or “liver disorder?”) and (stroke* or dementia or “cardiac arrhythmia*” or an?emia* or obesity or obese or “prostatic hyperplasia” or “prostatic hypertrophy” or tuberculosis or “chronic hepatitis” or “substance-related disorder?” or “substance abuse” or “drug abuse” or “marijuana abuse” or “cocaine abuse” or “amphetamine abuse” or addiction? or “inflammatory bowel disease?” or “ulcerative colitis” or crohn or ibd or deaf* or blind* or “visual loss” or parkinson* or autis*))
18. TITLE-ABS-KEY((stroke*) and (dementia or “cardiac arrhythmia*” or an?emia* or obesity or obese or “prostatic hyperplasia” or “prostatic hypertrophy” or tuberculosis or “chronic hepatitis” or “substance-related disorder?” or “substance abuse” or “drug abuse” or “marijuana abuse” or “cocaine abuse” or “amphetamine abuse” or addiction? or “inflammatory bowel disease?” or “ulcerative colitis” or crohn or ibd or deaf* or blind* or “visual loss” or parkinson* or autis*))
19. TITLE-ABS-KEY((dementia) and (“cardiac arrhythmia*” or an?emia* or obesity or obese or “prostatic hyperplasia” or “prostatic hypertrophy” or tuberculosis or “chronic hepatitis” or “substance-related disorder?” or “substance abuse” or “drug abuse” or “marijuana abuse” or “cocaine abuse” or “amphetamine abuse” or addiction? or “inflammatory bowel disease?” or “ulcerative colitis” or crohn or ibd or deaf* or blind* or “visual loss” or parkinson* or autis*))
20. TITLE-ABS-KEY((“cardiac arrhythmia*”) and (an?emia* or obesity or obese or “prostatic hyperplasia” or “prostatic hypertrophy” or tuberculosis or “chronic hepatitis” or “substance-related disorder?” or “substance abuse” or “drug abuse” or “marijuana abuse” or “cocaine abuse” or “amphetamine abuse” or addiction? or “inflammatory bowel disease?” or “ulcerative colitis” or crohn or ibd or deaf* or blind* or “visual loss” or parkinson* or autis*))
21. TITLE-ABS-KEY((an?emia*) and (obesity or obese or “prostatic hyperplasia” or “prostatic hypertrophy” or tuberculosis or “chronic hepatitis” or “substance-related disorder?” or “substance abuse” or “drug abuse” or “marijuana abuse” or “cocaine abuse” or “amphetamine abuse” or addiction? or “inflammatory bowel disease?” or “ulcerative colitis” or crohn or ibd or deaf* or blind* or “visual loss” or parkinson* or autis*))
22. TITLE-ABS-KEY((obesity or obese) and (“prostatic hyperplasia” or “prostatic hypertrophy” or tuberculosis or “chronic hepatitis” or “substance-related disorder?” or “substance abuse” or “drug abuse” or “marijuana abuse” or “cocaine abuse” or “amphetamine abuse” or addiction? or “inflammatory bowel disease?” or “ulcerative colitis” or crohn or ibd or deaf* or blind* or “visual loss” or parkinson* or autis*))
23. TITLE-ABS-KEY((“prostatic hyperplasia” or “prostatic hypertrophy”) and (tuberculosis or “chronic hepatitis” or “substance-related disorder?” or “substance abuse” or “drug abuse” or “marijuana abuse” or “cocaine abuse” or “amphetamine abuse” or addiction? or “inflammatory bowel disease?” or “ulcerative colitis” or crohn or ibd or deaf* or blind* or “visual loss” or parkinson* or autis*))
24. TITLE-ABS-KEY((tuberculosis) and (“chronic hepatitis” or “substance-related disorder?” or “substance abuse” or “drug abuse” or “marijuana abuse” or “cocaine abuse” or “amphetamine abuse” or addiction? or “inflammatory bowel disease?” or “ulcerative colitis” or crohn or ibd or deaf* or blind* or “visual loss” or parkinson* or autis*))
25. TITLE-ABS-KEY((“chronic hepatitis”) and (“substance-related disorder?” or “substance abuse” or “drug abuse” or “marijuana abuse” or “cocaine abuse” or “amphetamine abuse” or addiction? or “inflammatory bowel disease?” or “ulcerative colitis” or crohn or ibd or deaf* or blind* or “visual loss” or parkinson* or autis*))
26. TITLE-ABS-KEY((“substance-related disorder?” or “substance abuse” or “drug abuse” or “marijuana abuse” or “cocaine abuse” or “amphetamine abuse” or addiction?) and (“inflammatory bowel disease?” or “ulcerative colitis” or crohn or ibd or deaf* or blind* or “visual loss” or parkinson* or autis*))
27. TITLE-ABS-KEY((“inflammatory bowel disease?” or “ulcerative colitis” or crohn or ibd) and (deaf* or blind* or “visual loss” or parkinson* or autis*))
28. TITLE-ABS-KEY((deaf*) and (blind* or “visual loss” or parkinson* or autis*))
29. TITLE-ABS-KEY((blind* or “visual loss”) and (parkinson* or autis*))
30. TITLE-ABS-KEY(parkinson* and autis*)
31. TITLE-ABS-KEY((condition* or diagnos* or disease* or illness* or “health problem*” or patholog* or disorder* or syndrome*) W/1 (associated or coexisting or co-existing or comorbid or co-morbid or concurrent or co-occuring or cooccuring or multiple) or “associated morbidit*”)
32. TITLE-ABS-KEY(comorbidit* or co-morbidit* or multidisease* or multi-disease* or multimorbidit* or multi-morbidit* or multipatholog* or multi-patholog* or pluripatholog* or polypatholog* or poly-pathology*)
33. #1 OR #2 OR #3 OR #4 OR #5 OR #6 OR #7 OR #8 OR #9 OR #10 OR #11 OR #12 OR #13 OR #14 OR #15 OR #16 OR #17 OR #18 OR #19 OR #20 OR #21 OR #22 OR #23 OR #24 OR #25 OR #26 OR #27 OR #28 OR #29 OR #30
34. 31 or 32
35. TITLE-ABS-KEY(cost* or ((health or health-care or healthcare or medical) W/3 expenditure*) or expense* or “hospital resource*”)
36. TITLE-ABS-KEY(((resource* or "health service") W/3 ("use" or utilisation or utilization)) or ((financial or economic) W/3 (burden or impact or consequence)) or spending or “out of pocket” or out-of-pocket or oop or insurance or absenteeism or (productivity W/3 (loss or lost)) or "economic modelling")
37. 35 or 36
38. 33 and 34 and 37 AND NOT INDEX(medline)
39. limit 38 to yr="2010 -Current"Web of science
40. TS=((“chronic obstructive pulmonary disease” or copd or “pulmonary disease?” or “pulmonary disorder?”) and (diabet* or hypertens* or "high blood pressure*" or neoplasm? or cancer? or ((cardiac or cardiovascular or coronary) NEAR/1 (disease? or disorder? or failure)) or “myocardial infarct*” or “cardiovascular strok*” or “heart failure” or “cardiac failure” or “myocardial failure” or asthma* or ((mental or anxiety or mood or psychological) NEAR/1 (disease? or disorder?)) or “sleep disorder” or depression? or osteoporosis or dyslipid?emia* or hyperlipid?emia* or hypercholesterolemia* or hypertriglyceridemia* or “thyroid disease?” or “thyroid disorder?” or hyperthyroid* or hypothyroid* or “joint disease*” or “rheumatoid arthritis” or osteoarthritis or “kidney disease?” or “kidney disorder?” or epilep* or seizure? or hiv or “acquired immun* deficiency syndrome?” or aids or “liver disease?” or “liver disorder?” or stroke* or dementia or “cardiac arrhythmia*” or an?emia* or obesity or obese or “prostatic hyperplasia” or “prostatic hypertrophy” or tuberculosis or “chronic hepatitis” or “substance-related disorder?” or “substance abuse” or “drug abuse” or “marijuana abuse” or “cocaine abuse” or “amphetamine abuse” or addiction? or “inflammatory bowel disease?” or “ulcerative colitis” or crohn or ibd or deaf* or blind* or “visual loss” or parkinson* or autis*))
41. TS=((diabet*) AND (hypertens* or "high blood pressure*" or neoplasm? or cancer? or ((cardiac or cardiovascular or coronary) NEAR/1 (disease? or disorder? or failure)) or “myocardial infarct*” or “cardiovascular strok*” or “heart failure” or “cardiac failure” or “myocardial failure” or asthma* or ((mental or anxiety or mood or psychological) NEAR/1 (disease? or disorder?)) or “sleep disorder” or depression? or osteoporosis or dyslipid?emia* or hyperlipid?emia* or hypercholesterolemia* or hypertriglyceridemia* or “thyroid disease?” or “thyroid disorder?” or hyperthyroid* or hypothyroid* or “joint disease*” or “rheumatoid arthritis” or osteoarthritis or “kidney disease?” or “kidney disorder?” or epilep* or seizure? or hiv or “acquired immun* deficiency syndrome?” or aids or “liver disease?” or “liver disorder?” or stroke* or dementia or “cardiac arrhythmia*” or an?emia* or obesity or obese or “prostatic hyperplasia” or “prostatic hypertrophy” or tuberculosis or “chronic hepatitis” or “substance-related disorder?” or “substance abuse” or “drug abuse” or “marijuana abuse” or “cocaine abuse” or “amphetamine abuse” or addiction? or “inflammatory bowel disease?” or “ulcerative colitis” or crohn or ibd or deaf* or blind* or “visual loss” or parkinson* or autis*))
42. TS=((hypertens* or "high blood pressure*") AND (neoplasm? or cancer? or ((cardiac or cardiovascular or coronary) NEAR/1 (disease? or disorder? or failure)) or “myocardial infarct*” or “cardiovascular strok*” or “heart failure” or “cardiac failure” or “myocardial failure” or asthma* or ((mental or anxiety or mood or psychological) NEAR/1 (disease? or disorder?)) or “sleep disorder” or depression? or osteoporosis or dyslipid?emia* or hyperlipid?emia* or hypercholesterolemia* or hypertriglyceridemia* or “thyroid disease?” or “thyroid disorder?” or hyperthyroid* or hypothyroid* or “joint disease*” or “rheumatoid arthritis” or osteoarthritis or “kidney disease?” or “kidney disorder?” or epilep* or seizure? or hiv or “acquired immun* deficiency syndrome?” or aids or “liver disease?” or “liver disorder?” or stroke* or dementia or “cardiac arrhythmia*” or an?emia* or obesity or obese or “prostatic hyperplasia” or “prostatic hypertrophy” or tuberculosis or “chronic hepatitis” or “substance-related disorder?” or “substance abuse” or “drug abuse” or “marijuana abuse” or “cocaine abuse” or “amphetamine abuse” or addiction? or “inflammatory bowel disease?” or “ulcerative colitis” or crohn or ibd or deaf* or blind* or “visual loss” or parkinson* or autis*))
43. TS=((neoplasm? or cancer?) AND (((cardiac or cardiovascular or coronary) NEAR/1 (disease? or disorder? or failure)) or “myocardial infarct*” or “cardiovascular strok*” or “heart failure” or “cardiac failure” or “myocardial failure” or asthma* or ((mental or anxiety or mood or psychological) NEAR/1 (disease? or disorder?)) or “sleep disorder” or depression? or osteoporosis or dyslipid?emia* or hyperlipid?emia* or hypercholesterolemia* or hypertriglyceridemia* or “thyroid disease?” or “thyroid disorder?” or hyperthyroid* or hypothyroid* or “joint disease*” or “rheumatoid arthritis” or osteoarthritis or “kidney disease?” or “kidney disorder?” or epilep* or seizure? or hiv or “acquired immun* deficiency syndrome?” or aids or “liver disease?” or “liver disorder?” or stroke* or dementia or “cardiac arrhythmia*” or an?emia* or obesity or obese or “prostatic hyperplasia” or “prostatic hypertrophy” or tuberculosis or “chronic hepatitis” or “substance-related disorder?” or “substance abuse” or “drug abuse” or “marijuana abuse” or “cocaine abuse” or “amphetamine abuse” or addiction? or “inflammatory bowel disease?” or “ulcerative colitis” or crohn or ibd or deaf* or blind* or “visual loss” or parkinson* or autis*))
44. TS=(((cardiac or cardiovascular or coronary) NEAR/1 (disease? or disorder? or failure)) AND (“myocardial infarct*” or “cardiovascular strok*” or “heart failure” or “cardiac failure” or “myocardial failure” or asthma* or ((mental or anxiety or mood or psychological) NEAR/1 (disease? or disorder?)) or “sleep disorder” or depression? or osteoporosis or dyslipid?emia* or hyperlipid?emia* or hypercholesterolemia* or hypertriglyceridemia* or “thyroid disease?” or “thyroid disorder?” or hyperthyroid* or hypothyroid* or “joint disease*” or “rheumatoid arthritis” or osteoarthritis or “kidney disease?” or “kidney disorder?” or epilep* or seizure? or hiv or “acquired immun* deficiency syndrome?” or aids or “liver disease?” or “liver disorder?” or stroke* or dementia or “cardiac arrhythmia*” or an?emia* or obesity or obese or “prostatic hyperplasia” or “prostatic hypertrophy” or tuberculosis or “chronic hepatitis” or “substance-related disorder?” or “substance abuse” or “drug abuse” or “marijuana abuse” or “cocaine abuse” or “amphetamine abuse” or addiction? or “inflammatory bowel disease?” or “ulcerative colitis” or crohn or ibd or deaf* or blind* or “visual loss” or parkinson* or autis*))
45. TS=((“myocardial infarct*” or “cardiovascular strok*”) and (“heart failure” or “cardiac failure” or “myocardial failure” or asthma* or ((mental or anxiety or mood or psychological) NEAR/1 (disease? or disorder?)) or “sleep disorder” or depression? or osteoporosis or dyslipid?emia* or hyperlipid?emia* or hypercholesterolemia* or hypertriglyceridemia* or “thyroid disease?” or “thyroid disorder?” or hyperthyroid* or hypothyroid* or “joint disease*” or “rheumatoid arthritis” or osteoarthritis or “kidney disease?” or “kidney disorder?” or epilep* or seizure? or hiv or “acquired immun* deficiency syndrome?” or aids or “liver disease?” or “liver disorder?” or stroke* or dementia or “cardiac arrhythmia*” or an?emia* or obesity or obese or “prostatic hyperplasia” or “prostatic hypertrophy” or tuberculosis or “chronic hepatitis” or “substance-related disorder?” or “substance abuse” or “drug abuse” or “marijuana abuse” or “cocaine abuse” or “amphetamine abuse” or addiction? or “inflammatory bowel disease?” or “ulcerative colitis” or crohn or ibd or deaf* or blind* or “visual loss” or parkinson* or autis*))
46. TS=((“heart failure” or “cardiac failure” or “myocardial failure”) and (asthma* or ((mental or anxiety or mood or psychological) NEAR/1 (disease? or disorder?)) or “sleep disorder” or depression? or osteoporosis or dyslipid?emia* or hyperlipid?emia* or hypercholesterolemia* or hypertriglyceridemia* or “thyroid disease?” or “thyroid disorder?” or hyperthyroid* or hypothyroid* or “joint disease*” or “rheumatoid arthritis” or osteoarthritis or “kidney disease?” or “kidney disorder?” or epilep* or seizure? or hiv or “acquired immun* deficiency syndrome?” or aids or “liver disease?” or “liver disorder?” or stroke* or dementia or “cardiac arrhythmia*” or an?emia* or obesity or obese or “prostatic hyperplasia” or “prostatic hypertrophy” or tuberculosis or “chronic hepatitis” or “substance-related disorder?” or “substance abuse” or “drug abuse” or “marijuana abuse” or “cocaine abuse” or “amphetamine abuse” or addiction? or “inflammatory bowel disease?” or “ulcerative colitis” or crohn or ibd or deaf* or blind* or “visual loss” or parkinson* or autis*))
47. TS=((asthma*) AND (((mental or anxiety or mood or psychological) NEAR/1 (disease? or disorder?)) or “sleep disorder” or depression? or osteoporosis or dyslipid?emia* or hyperlipid?emia* or hypercholesterolemia* or hypertriglyceridemia* or “thyroid disease?” or “thyroid disorder?” or hyperthyroid* or hypothyroid* or “joint disease*” or “rheumatoid arthritis” or osteoarthritis or “kidney disease?” or “kidney disorder?” or epilep* or seizure? or hiv or “acquired immun* deficiency syndrome?” or aids or “liver disease?” or “liver disorder?” or stroke* or dementia or “cardiac arrhythmia*” or an?emia* or obesity or obese or “prostatic hyperplasia” or “prostatic hypertrophy” or tuberculosis or “chronic hepatitis” or “substance-related disorder?” or “substance abuse” or “drug abuse” or “marijuana abuse” or “cocaine abuse” or “amphetamine abuse” or addiction? or “inflammatory bowel disease?” or “ulcerative colitis” or crohn or ibd or deaf* or blind* or “visual loss” or parkinson* or autis*))
48. TS=((((mental or anxiety or mood or psychological) NEAR/1 (disease? or disorder?)) or “sleep disorder” or depression?) and (osteoporosis or dyslipid?emia* or hyperlipid?emia* or hypercholesterolemia* or hypertriglyceridemia* or “thyroid disease?” or “thyroid disorder?” or hyperthyroid* or hypothyroid* or “joint disease*” or “rheumatoid arthritis” or osteoarthritis or “kidney disease?” or “kidney disorder?” or epilep* or seizure? or hiv or “acquired immun* deficiency syndrome?” or aids or “liver disease?” or “liver disorder?” or stroke* or dementia or “cardiac arrhythmia*” or an?emia* or obesity or obese or “prostatic hyperplasia” or “prostatic hypertrophy” or tuberculosis or “chronic hepatitis” or “substance-related disorder?” or “substance abuse” or “drug abuse” or “marijuana abuse” or “cocaine abuse” or “amphetamine abuse” or addiction? or “inflammatory bowel disease?” or “ulcerative colitis” or crohn or ibd or deaf* or blind* or “visual loss” or parkinson* or autis*))
49. TS=((osteoporosis) and (dyslipid?emia* or hyperlipid?emia* or hypercholesterolemia* or hypertriglyceridemia* or “thyroid disease?” or “thyroid disorder?” or hyperthyroid* or hypothyroid* or “joint disease*” or “rheumatoid arthritis” or osteoarthritis or “kidney disease?” or “kidney disorder?” or epilep* or seizure? or hiv or “acquired immun* deficiency syndrome?” or aids or “liver disease?” or “liver disorder?” or stroke* or dementia or “cardiac arrhythmia*” or an?emia* or obesity or obese or “prostatic hyperplasia” or “prostatic hypertrophy” or tuberculosis or “chronic hepatitis” or “substance-related disorder?” or “substance abuse” or “drug abuse” or “marijuana abuse” or “cocaine abuse” or “amphetamine abuse” or addiction? or “inflammatory bowel disease?” or “ulcerative colitis” or crohn or ibd or deaf* or blind* or “visual loss” or parkinson* or autis*))
50. TS=((dyslipid?emia* or hyperlipid?emia* or hypercholesterolemia* or hypertriglyceridemia*) and (“thyroid disease?” or “thyroid disorder?” or hyperthyroid* or hypothyroid* or “joint disease*” or “rheumatoid arthritis” or osteoarthritis or “kidney disease?” or “kidney disorder?” or epilep* or seizure? or hiv or “acquired immun* deficiency syndrome?” or aids or “liver disease?” or “liver disorder?” or stroke* or dementia or “cardiac arrhythmia*” or an?emia* or obesity or obese or “prostatic hyperplasia” or “prostatic hypertrophy” or tuberculosis or “chronic hepatitis” or “substance-related disorder?” or “substance abuse” or “drug abuse” or “marijuana abuse” or “cocaine abuse” or “amphetamine abuse” or addiction? or “inflammatory bowel disease?” or “ulcerative colitis” or crohn or ibd or deaf* or blind* or “visual loss” or parkinson* or autis*))
51. TS=((“thyroid disease?” or “thyroid disorder?” or hyperthyroid* or hypothyroid*) and (“joint disease*” or “rheumatoid arthritis” or osteoarthritis or “kidney disease?” or “kidney disorder?” or epilep* or seizure? or hiv or “acquired immun* deficiency syndrome?” or aids or “liver disease?” or “liver disorder?” or stroke* or dementia or “cardiac arrhythmia*” or an?emia* or obesity or obese or “prostatic hyperplasia” or “prostatic hypertrophy” or tuberculosis or “chronic hepatitis” or “substance-related disorder?” or “substance abuse” or “drug abuse” or “marijuana abuse” or “cocaine abuse” or “amphetamine abuse” or addiction? or “inflammatory bowel disease?” or “ulcerative colitis” or crohn or ibd or deaf* or blind* or “visual loss” or parkinson* or autis*))
52. TS=((“joint disease*” or “rheumatoid arthritis” or osteoarthritis) and (“kidney disease?” or “kidney disorder?” or epilep* or seizure? or hiv or “acquired immun* deficiency syndrome?” or aids or “liver disease?” or “liver disorder?” or stroke* or dementia or “cardiac arrhythmia*” or an?emia* or obesity or obese or “prostatic hyperplasia” or “prostatic hypertrophy” or tuberculosis or “chronic hepatitis” or “substance-related disorder?” or “substance abuse” or “drug abuse” or “marijuana abuse” or “cocaine abuse” or “amphetamine abuse” or addiction? or “inflammatory bowel disease?” or “ulcerative colitis” or crohn or ibd or deaf* or blind* or “visual loss” or parkinson* or autis*))
53. TS=((“kidney disease?” or “kidney disorder?”) and (epilep* or seizure? or hiv or “acquired immun* deficiency syndrome?” or aids or “liver disease?” or “liver disorder?” or stroke* or dementia or “cardiac arrhythmia*” or an?emia* or obesity or obese or “prostatic hyperplasia” or “prostatic hypertrophy” or tuberculosis or “chronic hepatitis” or “substance-related disorder?” or “substance abuse” or “drug abuse” or “marijuana abuse” or “cocaine abuse” or “amphetamine abuse” or addiction? or “inflammatory bowel disease?” or “ulcerative colitis” or crohn or ibd or deaf* or blind* or “visual loss” or parkinson* or autis*))
54. TS=((epilep* or seizure?) and (hiv or “acquired immun* deficiency syndrome?” or aids or “liver disease?” or “liver disorder?” or stroke* or dementia or “cardiac arrhythmia*” or an?emia* or obesity or obese or “prostatic hyperplasia” or “prostatic hypertrophy” or tuberculosis or “chronic hepatitis” or “substance-related disorder?” or “substance abuse” or “drug abuse” or “marijuana abuse” or “cocaine abuse” or “amphetamine abuse” or addiction? or “inflammatory bowel disease?” or “ulcerative colitis” or crohn or ibd or deaf* or blind* or “visual loss” or parkinson* or autis*))
55. TS=((hiv or “acquired immun* deficiency syndrome?” or aids) and (“liver disease?” or “liver disorder?” or stroke* or dementia or “cardiac arrhythmia*” or an?emia* or obesity or obese or “prostatic hyperplasia” or “prostatic hypertrophy” or tuberculosis or “chronic hepatitis” or “substance-related disorder?” or “substance abuse” or “drug abuse” or “marijuana abuse” or “cocaine abuse” or “amphetamine abuse” or addiction? or “inflammatory bowel disease?” or “ulcerative colitis” or crohn or ibd or deaf* or blind* or “visual loss” or parkinson* or autis*))
56. TS=((“liver disease?” or “liver disorder?”) and (stroke* or dementia or “cardiac arrhythmia*” or an?emia* or obesity or obese or “prostatic hyperplasia” or “prostatic hypertrophy” or tuberculosis or “chronic hepatitis” or “substance-related disorder?” or “substance abuse” or “drug abuse” or “marijuana abuse” or “cocaine abuse” or “amphetamine abuse” or addiction? or “inflammatory bowel disease?” or “ulcerative colitis” or crohn or ibd or deaf* or blind* or “visual loss” or parkinson* or autis*))
57. TS=((stroke*) and (dementia or “cardiac arrhythmia*” or an?emia* or obesity or obese or “prostatic hyperplasia” or “prostatic hypertrophy” or tuberculosis or “chronic hepatitis” or “substance-related disorder?” or “substance abuse” or “drug abuse” or “marijuana abuse” or “cocaine abuse” or “amphetamine abuse” or addiction? or “inflammatory bowel disease?” or “ulcerative colitis” or crohn or ibd or deaf* or blind* or “visual loss” or parkinson* or autis*))
58. TS=((dementia) and (“cardiac arrhythmia*” or an?emia* or obesity or obese or “prostatic hyperplasia” or “prostatic hypertrophy” or tuberculosis or “chronic hepatitis” or “substance-related disorder?” or “substance abuse” or “drug abuse” or “marijuana abuse” or “cocaine abuse” or “amphetamine abuse” or addiction? or “inflammatory bowel disease?” or “ulcerative colitis” or crohn or ibd or deaf* or blind* or “visual loss” or parkinson* or autis*))
59. TS=((“cardiac arrhythmia*”) and (an?emia* or obesity or obese or “prostatic hyperplasia” or “prostatic hypertrophy” or tuberculosis or “chronic hepatitis” or “substance-related disorder?” or “substance abuse” or “drug abuse” or “marijuana abuse” or “cocaine abuse” or “amphetamine abuse” or addiction? or “inflammatory bowel disease?” or “ulcerative colitis” or crohn or ibd or deaf* or blind* or “visual loss” or parkinson* or autis*))
60. TS=((an?emia*) and (obesity or obese or “prostatic hyperplasia” or “prostatic hypertrophy” or tuberculosis or “chronic hepatitis” or “substance-related disorder?” or “substance abuse” or “drug abuse” or “marijuana abuse” or “cocaine abuse” or “amphetamine abuse” or addiction? or “inflammatory bowel disease?” or “ulcerative colitis” or crohn or ibd or deaf* or blind* or “visual loss” or parkinson* or autis*))
61. TS=((obesity or obese) and (“prostatic hyperplasia” or “prostatic hypertrophy” or tuberculosis or “chronic hepatitis” or “substance-related disorder?” or “substance abuse” or “drug abuse” or “marijuana abuse” or “cocaine abuse” or “amphetamine abuse” or addiction? or “inflammatory bowel disease?” or “ulcerative colitis” or crohn or ibd or deaf* or blind* or “visual loss” or parkinson* or autis*))
62. TS=((“prostatic hyperplasia” or “prostatic hypertrophy”) and (tuberculosis or “chronic hepatitis” or “substance-related disorder?” or “substance abuse” or “drug abuse” or “marijuana abuse” or “cocaine abuse” or “amphetamine abuse” or addiction? or “inflammatory bowel disease?” or “ulcerative colitis” or crohn or ibd or deaf* or blind* or “visual loss” or parkinson* or autis*))
63. TS=((tuberculosis) and (“chronic hepatitis” or “substance-related disorder?” or “substance abuse” or “drug abuse” or “marijuana abuse” or “cocaine abuse” or “amphetamine abuse” or addiction? or “inflammatory bowel disease?” or “ulcerative colitis” or crohn or ibd or deaf* or blind* or “visual loss” or parkinson* or autis*))
64. TS=((“chronic hepatitis”) and (“substance-related disorder?” or “substance abuse” or “drug abuse” or “marijuana abuse” or “cocaine abuse” or “amphetamine abuse” or addiction? or “inflammatory bowel disease?” or “ulcerative colitis” or crohn or ibd or deaf* or blind* or “visual loss” or parkinson* or autis*))
65. TS=((“substance-related disorder?” or “substance abuse” or “drug abuse” or “marijuana abuse” or “cocaine abuse” or “amphetamine abuse” or addiction?) and (“inflammatory bowel disease?” or “ulcerative colitis” or crohn or ibd or deaf* or blind* or “visual loss” or parkinson* or autis*))
66. TS=((“inflammatory bowel disease?” or “ulcerative colitis” or crohn or ibd) and (deaf* or blind* or “visual loss” or parkinson* or autis*))
67. TS=((deaf*) and (blind* or “visual loss” or parkinson* or autis*))
68. TS=((blind* or “visual loss”) and (parkinson* or autis*))
69. TS=(parkinson* and autis*)
70. TS=((condition* or diagnos* or disease* or illness* or “health problem*” or patholog* or disorder* or syndrome*) NEAR/1 (associated or coexisting or co-existing or comorbid or co-morbid or concurrent or co-occuring or cooccuring or multiple) or “associated morbidit*”)
71. TS=(comorbidit* or co-morbidit* or multidisease* or multi-disease* or multimorbidit* or multi-morbidit* or multipatholog* or multi-patholog* or pluripatholog* or polypatholog* or poly-pathology*)
72. 1 or 2 or 3 or 4 or 5 or 6 or 7 or 8 or 9 or 10 or 11 or 12 or 13 or 14 or 15 or 16 or 17 or 18 or 19 or 20 or 21 or 22 or 23 or 24 or 25 or 26 or 27 or 28 or 29 or 30
73. 31 or 32
74. TS=(cost* or ((health or health-care or healthcare or medical) NEAR/3 expenditure*) or expense* or “hospital resource*”)
75. TS=(((resource* or "health service") NEAR/3 ("use" or utilisation or utilization)) or ((financial or economic) NEAR/3 (burden or impact or consequence)) or spending or “out of pocket” or out-of-pocket or oop or insurance or absenteeism or (productivity NEAR/3 (loss or lost)) or "economic modelling")
76. 35 or 36
77. 33 and 34 and 37
78. limit 38 to yr="2010 -Current"

Database: Ovid MEDLINE(R) ALL <1946 to August 31, 2020>

Search Strategy:

--------------------------------------------------------------------------------

1 (pulmonary disease, chronic obstructive/ or (copd or (pulmonary adj2 (disease? or disorder?))).ti,ab.) and (diabetes mellitus/ or diabet*.ti,ab. or hypertension/ or (hypertens* or "high blood pressure?").ti,ab. or neoplasms/ or (neoplasm? or cancer?).ti,ab. or coronary disease/ or ((cardiac or cardiovascular or coronary) adj1 (disease? or disorder? or failure)).ti,ab. or exp myocardial infarction/ or (myocardial infarct* or cardiovascular strok*).ti,ab. or exp heart failure/ or ((heart or cardiac or myocardial) adj1 failure).ti,ab. or asthma/ or asthma*.ti,ab. or anxiety/ or depression/ or (((mental or anxiety or mood or psychological or sleep) adj1 (disease? or disorder?)) or depression?).ti,ab. or osteoporosis/ or osteoporosis.ti,ab. or exp dyslipidemias/ or (dyslipid?emia* or hyperlipid?emia* or hypercholesterolemia* or hypertriglyceridemia*).ti,ab. or exp thyroid diseases/ or ((thyroid adj1 (disease? or disorder?)) or hyperthyroid* or hypothyroid*).ti,ab. or joint diseases/ or osteoarthritis/ or arthritis rheumatoid/ or (joint disease* or rheumatoid arthritis or osteoarthritis).ti,ab. or exp kidney diseases/ or (kidney adj1 (disease? or disorder?)).ti,ab. or epilepsy/ or (epilep* or seizure?).ti,ab. or hiv infections/ or (hiv or acquired immun* deficiency syndrome? or aids).ti,ab. or exp liver diseases/ or (liver adj1 (disease? or disorder?)).ti,ab. or stroke/ or stroke*.ti,ab. or dementia/ or dementia.ti,ab. or exp arrhythmias, cardiac/ or cardiac arrhythmia*.ti,ab. or anemia/ or an?emia*.ti,ab. or obesity/ or (obesity or obese).ti,ab. or prostatic hyperplasia/ or (prostatic adj1 (hyperplasia or hypertrophy)).ti,ab. or tuberculosis/ or tuberculosis.ti,ab. or exp chronic hepatitis/ or chronic hepatitis.ti,ab. or substance-related disorders/ or (((substance or drug or marijuana or cocaine or amphetamine) adj2 abuse) or "substance abuse" or addiction?).ti,ab. or inflammatory bowel diseases/ or (inflammatory bowel disease? or ulcerative colitis or crohn or ibd).ti,ab. or deafness/ or deaf*.ti,ab. or blindness/ or (blind* or (visual adj1 loss)).ti,ab. or parkinson disease/ or parkinson*.ti,ab. or exp autism spectrum disorder/ or autis*.ti,ab.) (43488)

2 (diabetes mellitus/ or diabet*.ti,ab.) and (hypertension/ or (hypertens* or "high blood pressure?").ti,ab. or neoplasms/ or (neoplasm? or cancer?).ti,ab. or coronary disease/ or ((cardiac or cardiovascular or coronary) adj1 (disease? or disorder? or failure)).ti,ab. or exp myocardial infarction/ or (myocardial infarct* or cardiovascular strok*).ti,ab. or exp heart failure/ or ((heart or cardiac or myocardial) adj1 failure).ti,ab. or asthma/ or asthma*.ti,ab. or anxiety/ or depression/ or (((mental or anxiety or mood or psychological or sleep) adj1 (disease? or disorder?)) or depression?).ti,ab. or osteoporosis/ or osteoporosis.ti,ab. or exp dyslipidemias/ or (dyslipid?emia* or hyperlipid?emia* or hypercholesterolemia* or hypertriglyceridemia*).ti,ab. or exp thyroid diseases/ or ((thyroid adj1 (disease? or disorder?)) or hyperthyroid* or hypothyroid*).ti,ab. or joint diseases/ or osteoarthritis/ or arthritis rheumatoid/ or (joint disease* or rheumatoid arthritis or osteoarthritis).ti,ab. or exp kidney diseases/ or (kidney adj1 (disease? or disorder?)).ti,ab. or epilepsy/ or (epilep* or seizure?).ti,ab. or hiv infections/ or (hiv or acquired immun* deficiency syndrome? or aids).ti,ab. or exp liver diseases/ or (liver adj1 (disease? or disorder?)).ti,ab. or stroke/ or stroke*.ti,ab. or dementia/ or dementia.ti,ab. or exp arrhythmias, cardiac/ or cardiac arrhythmia*.ti,ab. or anemia/ or an?emia*.ti,ab. or obesity/ or (obesity or obese).ti,ab. or prostatic hyperplasia/ or (prostatic adj1 (hyperplasia or hypertrophy)).ti,ab. or tuberculosis/ or tuberculosis.ti,ab. or exp chronic hepatitis/ or chronic hepatitis.ti,ab. or substance-related disorders/ or (((substance or drug or marijuana or cocaine or amphetamine) adj2 abuse) or "substance abuse" or addiction?).ti,ab. or inflammatory bowel diseases/ or (inflammatory bowel disease? or ulcerative colitis or crohn or ibd).ti,ab. or deafness/ or deaf*.ti,ab. or blindness/ or (blind* or (visual adj1 loss)).ti,ab. or parkinson disease/ or parkinson*.ti,ab. or exp autism spectrum disorder/ or autis*.ti,ab.) (462964)

3 (neoplasms/ or (neoplasm? or cancer?).ti,ab.) and (coronary disease/ or ((cardiac or cardiovascular or coronary) adj1 (disease? or disorder? or failure)).ti,ab. or exp myocardial infarction/ or (myocardial infarct* or cardiovascular strok*).ti,ab. or exp heart failure/ or ((heart or cardiac or myocardial) adj1 failure).ti,ab. or asthma/ or asthma*.ti,ab. or anxiety/ or depression/ or (((mental or anxiety or mood or psychological or sleep) adj1 (disease? or disorder?)) or depression?).ti,ab. or osteoporosis/ or osteoporosis.ti,ab. or exp dyslipidemias/ or (dyslipid?emia* or hyperlipid?emia* or hypercholesterolemia* or hypertriglyceridemia*).ti,ab. or exp thyroid diseases/ or ((thyroid adj1 (disease? or disorder?)) or hyperthyroid* or hypothyroid*).ti,ab. or joint diseases/ or osteoarthritis/ or arthritis rheumatoid/ or (joint disease* or rheumatoid arthritis or osteoarthritis).ti,ab. or exp kidney diseases/ or (kidney adj1 (disease? or disorder?)).ti,ab. or epilepsy/ or (epilep* or seizure?).ti,ab. or hiv infections/ or (hiv or acquired immun* deficiency syndrome? or aids).ti,ab. or exp liver diseases/ or (liver adj1 (disease? or disorder?)).ti,ab. or stroke/ or stroke*.ti,ab. or dementia/ or dementia.ti,ab. or exp arrhythmias, cardiac/ or cardiac arrhythmia*.ti,ab. or anemia/ or an?emia*.ti,ab. or obesity/ or (obesity or obese).ti,ab. or prostatic hyperplasia/ or (prostatic adj1 (hyperplasia or hypertrophy)).ti,ab. or tuberculosis/ or tuberculosis.ti,ab. or exp chronic hepatitis/ or chronic hepatitis.ti,ab. or substance-related disorders/ or (((substance or drug or marijuana or cocaine or amphetamine) adj2 abuse) or "substance abuse" or addiction?).ti,ab. or inflammatory bowel diseases/ or (inflammatory bowel disease? or ulcerative colitis or crohn or ibd).ti,ab. or deafness/ or deaf*.ti,ab. or blindness/ or (blind* or (visual adj1 loss)).ti,ab. or parkinson disease/ or parkinson*.ti,ab. or exp autism spectrum disorder/ or autis*.ti,ab.) (271668)

4 (hypertension/ or (hypertens* or "high blood pressure?").ti,ab.) and (neoplasms/ or (neoplasm? or cancer?).ti,ab. or coronary disease/ or ((cardiac or cardiovascular or coronary) adj1 (disease? or disorder? or failure)).ti,ab. or exp myocardial infarction/ or (myocardial infarct* or cardiovascular strok*).ti,ab. or exp heart failure/ or ((heart or cardiac or myocardial) adj1 failure).ti,ab. or asthma/ or asthma*.ti,ab. or anxiety/ or depression/ or (((mental or anxiety or mood or psychological or sleep) adj1 (disease? or disorder?)) or depression?).ti,ab. or osteoporosis/ or osteoporosis.ti,ab. or exp dyslipidemias/ or (dyslipid?emia* or hyperlipid?emia* or hypercholesterolemia* or hypertriglyceridemia*).ti,ab. or exp thyroid diseases/ or ((thyroid adj1 (disease? or disorder?)) or hyperthyroid* or hypothyroid*).ti,ab. or joint diseases/ or osteoarthritis/ or arthritis rheumatoid/ or (joint disease* or rheumatoid arthritis or osteoarthritis).ti,ab. or exp kidney diseases/ or (kidney adj1 (disease? or disorder?)).ti,ab. or epilepsy/ or (epilep* or seizure?).ti,ab. or hiv infections/ or (hiv or acquired immun* deficiency syndrome? or aids).ti,ab. or exp liver diseases/ or (liver adj1 (disease? or disorder?)).ti,ab. or stroke/ or stroke*.ti,ab. or dementia/ or dementia.ti,ab. or exp arrhythmias, cardiac/ or cardiac arrhythmia*.ti,ab. or anemia/ or an?emia*.ti,ab. or obesity/ or (obesity or obese).ti,ab. or prostatic hyperplasia/ or (prostatic adj1 (hyperplasia or hypertrophy)).ti,ab. or tuberculosis/ or tuberculosis.ti,ab. or exp chronic hepatitis/ or chronic hepatitis.ti,ab. or substance-related disorders/ or (((substance or drug or marijuana or cocaine or amphetamine) adj2 abuse) or "substance abuse" or addiction?).ti,ab. or inflammatory bowel diseases/ or (inflammatory bowel disease? or ulcerative colitis or crohn or ibd).ti,ab. or deafness/ or deaf*.ti,ab. or blindness/ or (blind* or (visual adj1 loss)).ti,ab. or parkinson disease/ or parkinson*.ti,ab. or exp autism spectrum disorder/ or autis*.ti,ab.) (6748906)

5 (coronary disease/ or ((cardiac or cardiovascular or coronary) adj1 (disease? or disorder? or failure)).ti,ab.) and (exp Myocardial Infarction/ or (Myocardial Infarct* or cardiovascular strok*).ti,ab. or exp heart failure/ or ((heart or cardiac or myocardial) adj1 failure).ti,ab. or asthma/ or asthma*.ti,ab. or anxiety/ or depression/ or (((mental or anxiety or mood or psychological or sleep) adj1 (disease? or disorder?)) or depression?).ti,ab. or osteoporosis/ or osteoporosis.ti,ab. or exp dyslipidemias/ or (dyslipid?emia* or hyperlipid?emia* or hypercholesterolemia* or hypertriglyceridemia*).ti,ab. or exp thyroid diseases/ or ((thyroid adj1 (disease? or disorder?)) or hyperthyroid* or hypothyroid*).ti,ab. or joint diseases/ or osteoarthritis/ or arthritis rheumatoid/ or (joint disease* or rheumatoid arthritis or osteoarthritis).ti,ab. or exp kidney diseases/ or (kidney adj1 (disease? or disorder?)).ti,ab. or epilepsy/ or (epilep* or seizure?).ti,ab. or hiv infections/ or (HIV or acquired immun* deficiency syndrome? or aids).ti,ab. or exp liver diseases/ or (liver adj1 (disease? or disorder?)).ti,ab. or stroke/ or stroke*.ti,ab. or dementia/ or dementia.ti,ab. or exp arrhythmias, cardiac/ or cardiac arrhythmia*.ti,ab. or anemia/ or an?emia*.ti,ab. or obesity/ or (obesity or obese).ti,ab. or prostatic hyperplasia/ or (prostatic adj1 (hyperplasia or hypertrophy)).ti,ab. or tuberculosis/ or tuberculosis.ti,ab. or exp chronic hepatitis/ or chronic hepatitis.ti,ab. or Substance-Related Disorders/ or (((substance or drug or marijuana or cocaine or amphetamine) adj2 abuse) or "substance abuse" or addiction?).ti,ab. or Inflammatory Bowel Diseases/ or (Inflammatory Bowel Disease? or ulcerative colitis or crohn or IBD).ti,ab. or deafness/ or deaf*.ti,ab. or blindness/ or (blind* or (visual adj1 loss)).ti,ab. or Parkinson Disease/ or parkinson*.ti,ab. or exp Autism Spectrum Disorder/ or autis*.ti,ab.) (160888)

6 (exp myocardial infarction/ or (myocardial infarct* or cardiovascular strok*).ti,ab.) and (exp heart failure/ or ((heart or cardiac or myocardial) adj1 failure).ti,ab. or asthma/ or asthma*.ti,ab. or anxiety/ or depression/ or (((mental or anxiety or mood or psychological or sleep) adj1 (disease? or disorder?)) or depression?).ti,ab. or osteoporosis/ or osteoporosis.ti,ab. or exp dyslipidemias/ or (dyslipid?emia* or hyperlipid?emia* or hypercholesterolemia* or hypertriglyceridemia*).ti,ab. or exp thyroid diseases/ or ((thyroid adj1 (disease? or disorder?)) or hyperthyroid* or hypothyroid*).ti,ab. or joint diseases/ or osteoarthritis/ or arthritis rheumatoid/ or (joint disease* or rheumatoid arthritis or osteoarthritis).ti,ab. or exp kidney diseases/ or (kidney adj1 (disease? or disorder?)).ti,ab. or epilepsy/ or (epilep* or seizure?).ti,ab. or hiv infections/ or (hiv or acquired immun* deficiency syndrome? or aids).ti,ab. or exp liver diseases/ or (liver adj1 (disease? or disorder?)).ti,ab. or stroke/ or stroke*.ti,ab. or dementia/ or dementia.ti,ab. or exp arrhythmias, cardiac/ or cardiac arrhythmia*.ti,ab. or anemia/ or an?emia*.ti,ab. or obesity/ or (obesity or obese).ti,ab. or prostatic hyperplasia/ or (prostatic adj1 (hyperplasia or hypertrophy)).ti,ab. or tuberculosis/ or tuberculosis.ti,ab. or exp chronic hepatitis/ or chronic hepatitis.ti,ab. or substance-related disorders/ or (((substance or drug or marijuana or cocaine or amphetamine) adj2 abuse) or "substance abuse" or addiction?).ti,ab. or inflammatory bowel diseases/ or (inflammatory bowel disease? or ulcerative colitis or crohn or ibd).ti,ab. or deafness/ or deaf*.ti,ab. or blindness/ or (blind* or (visual adj1 loss)).ti,ab. or parkinson disease/ or parkinson*.ti,ab. or exp autism spectrum disorder/ or autis*.ti,ab.) (90193)

7 (exp heart failure/ or ((heart or cardiac or myocardial) adj1 failure).ti,ab.) and (asthma/ or asthma*.ti,ab. or anxiety/ or depression/ or (((mental or anxiety or mood or psychological or sleep) adj1 (disease? or disorder?)) or depression?).ti,ab. or osteoporosis/ or osteoporosis.ti,ab. or exp dyslipidemias/ or (dyslipid?emia* or hyperlipid?emia* or hypercholesterolemia* or hypertriglyceridemia*).ti,ab. or exp thyroid diseases/ or ((thyroid adj1 (disease? or disorder?)) or hyperthyroid* or hypothyroid*).ti,ab. or joint diseases/ or osteoarthritis/ or arthritis rheumatoid/ or (joint disease* or rheumatoid arthritis or osteoarthritis).ti,ab. or exp kidney diseases/ or (kidney adj1 (disease? or disorder?)).ti,ab. or epilepsy/ or (epilep* or seizure?).ti,ab. or hiv infections/ or (hiv or acquired immun* deficiency syndrome? or aids).ti,ab. or exp liver diseases/ or (liver adj1 (disease? or disorder?)).ti,ab. or stroke/ or stroke*.ti,ab. or dementia/ or dementia.ti,ab. or exp arrhythmias, cardiac/ or cardiac arrhythmia*.ti,ab. or anemia/ or an?emia*.ti,ab. or obesity/ or (obesity or obese).ti,ab. or prostatic hyperplasia/ or (prostatic adj1 (hyperplasia or hypertrophy)).ti,ab. or tuberculosis/ or tuberculosis.ti,ab. or exp chronic hepatitis/ or chronic hepatitis.ti,ab. or substance-related disorders/ or (((substance or drug or marijuana or cocaine or amphetamine) adj2 abuse) or "substance abuse" or addiction?).ti,ab. or inflammatory bowel diseases/ or (inflammatory bowel disease? or ulcerative colitis or crohn or ibd).ti,ab. or deafness/ or deaf*.ti,ab. or blindness/ or (blind* or (visual adj1 loss)).ti,ab. or parkinson disease/ or parkinson*.ti,ab. or exp autism spectrum disorder/ or autis*.ti,ab.) (61670)

8 (asthma/ or asthma*.ti,ab.) and (anxiety/ or depression/ or (((mental or anxiety or mood or psychological or sleep) adj1 (disease? or disorder?)) or depression?).ti,ab. or osteoporosis/ or osteoporosis.ti,ab. or exp dyslipidemias/ or (dyslipid?emia* or hyperlipid?emia* or hypercholesterolemia* or hypertriglyceridemia*).ti,ab. or exp thyroid diseases/ or ((thyroid adj1 (disease? or disorder?)) or hyperthyroid* or hypothyroid*).ti,ab. or joint diseases/ or osteoarthritis/ or arthritis rheumatoid/ or (joint disease* or rheumatoid arthritis or osteoarthritis).ti,ab. or exp kidney diseases/ or (kidney adj1 (disease? or disorder?)).ti,ab. or epilepsy/ or (epilep* or seizure?).ti,ab. or hiv infections/ or (hiv or acquired immun* deficiency syndrome? or aids).ti,ab. or exp liver diseases/ or (liver adj1 (disease? or disorder?)).ti,ab. or stroke/ or stroke*.ti,ab. or dementia/ or dementia.ti,ab. or exp arrhythmias, cardiac/ or cardiac arrhythmia*.ti,ab. or anemia/ or an?emia*.ti,ab. or obesity/ or (obesity or obese).ti,ab. or prostatic hyperplasia/ or (prostatic adj1 (hyperplasia or hypertrophy)).ti,ab. or tuberculosis/ or tuberculosis.ti,ab. or exp chronic hepatitis/ or chronic hepatitis.ti,ab. or substance-related disorders/ or (((substance or drug or marijuana or cocaine or amphetamine) adj2 abuse) or "substance abuse" or addiction?).ti,ab. or inflammatory bowel diseases/ or (inflammatory bowel disease? or ulcerative colitis or crohn or ibd).ti,ab. or deafness/ or deaf*.ti,ab. or blindness/ or (blind* or (visual adj1 loss)).ti,ab. or parkinson disease/ or parkinson*.ti,ab. or exp autism spectrum disorder/ or autis*.ti,ab.) (21229)

9 (anxiety/ or depression/ or (((mental or anxiety or mood or psychological or sleep) adj1 (disease? or disorder?)) or depression?).ti,ab.) and (osteoporosis/ or osteoporosis.ti,ab. or exp dyslipidemias/ or (dyslipid?emia* or hyperlipid?emia* or hypercholesterolemia* or hypertriglyceridemia*).ti,ab. or exp thyroid diseases/ or ((thyroid adj1 (disease? or disorder?)) or hyperthyroid* or hypothyroid*).ti,ab. or joint diseases/ or osteoarthritis/ or arthritis rheumatoid/ or (joint disease* or rheumatoid arthritis or osteoarthritis).ti,ab. or exp kidney diseases/ or (kidney adj1 (disease? or disorder?)).ti,ab. or epilepsy/ or (epilep* or seizure?).ti,ab. or hiv infections/ or (hiv or acquired immun* deficiency syndrome? or aids).ti,ab. or exp liver diseases/ or (liver adj1 (disease? or disorder?)).ti,ab. or stroke/ or stroke*.ti,ab. or dementia/ or dementia.ti,ab. or exp arrhythmias, cardiac/ or cardiac arrhythmia*.ti,ab. or anemia/ or an?emia*.ti,ab. or obesity/ or (obesity or obese).ti,ab. or prostatic hyperplasia/ or (prostatic adj1 (hyperplasia or hypertrophy)).ti,ab. or tuberculosis/ or tuberculosis.ti,ab. or exp chronic hepatitis/ or chronic hepatitis.ti,ab. or substance-related disorders/ or (((substance or drug or marijuana or cocaine or amphetamine) adj2 abuse) or "substance abuse" or addiction?).ti,ab. or inflammatory bowel diseases/ or (inflammatory bowel disease? or ulcerative colitis or crohn or ibd).ti,ab. or deafness/ or deaf*.ti,ab. or blindness/ or (blind* or (visual adj1 loss)).ti,ab. or parkinson disease/ or parkinson*.ti,ab. or exp autism spectrum disorder/ or autis*.ti,ab.) (100550)

10 (osteoporosis/ or osteoporosis.ti,ab.) and (exp dyslipidemias/ or (dyslipid?emia* or hyperlipid?emia* or hypercholesterolemia* or hypertriglyceridemia*).ti,ab. or exp thyroid diseases/ or ((thyroid adj1 (disease? or disorder?)) or hyperthyroid* or hypothyroid*).ti,ab. or joint diseases/ or osteoarthritis/ or arthritis rheumatoid/ or (joint disease* or rheumatoid arthritis or osteoarthritis).ti,ab. or exp kidney diseases/ or (kidney adj1 (disease? or disorder?)).ti,ab. or epilepsy/ or (epilep* or seizure?).ti,ab. or hiv infections/ or (hiv or acquired immun* deficiency syndrome? or aids).ti,ab. or exp liver diseases/ or (liver adj1 (disease? or disorder?)).ti,ab. or stroke/ or stroke*.ti,ab. or dementia/ or dementia.ti,ab. or exp arrhythmias, cardiac/ or cardiac arrhythmia*.ti,ab. or anemia/ or an?emia*.ti,ab. or obesity/ or (obesity or obese).ti,ab. or prostatic hyperplasia/ or (prostatic adj1 (hyperplasia or hypertrophy)).ti,ab. or tuberculosis/ or tuberculosis.ti,ab. or exp chronic hepatitis/ or chronic hepatitis.ti,ab. or substance-related disorders/ or (((substance or drug or marijuana or cocaine or amphetamine) adj2 abuse) or "substance abuse" or addiction?).ti,ab. or inflammatory bowel diseases/ or (inflammatory bowel disease? or ulcerative colitis or crohn or ibd).ti,ab. or deafness/ or deaf*.ti,ab. or blindness/ or (blind* or (visual adj1 loss)).ti,ab. or parkinson disease/ or parkinson*.ti,ab. or exp autism spectrum disorder/ or autis*.ti,ab.) (14012)

11 (exp dyslipidemias/ or (dyslipid?emia* or hyperlipid?emia* or hypercholesterolemia* or hypertriglyceridemia*).ti,ab.) and (exp thyroid diseases/ or ((thyroid adj1 (disease? or disorder?)) or hyperthyroid* or hypothyroid*).ti,ab. or joint diseases/ or osteoarthritis/ or arthritis rheumatoid/ or (joint disease* or rheumatoid arthritis or osteoarthritis).ti,ab. or exp kidney diseases/ or (kidney adj1 (disease? or disorder?)).ti,ab. or epilepsy/ or (epilep* or seizure?).ti,ab. or hiv infections/ or (hiv or acquired immun* deficiency syndrome? or aids).ti,ab. or exp liver diseases/ or (liver adj1 (disease? or disorder?)).ti,ab. or stroke/ or stroke*.ti,ab. or dementia/ or dementia.ti,ab. or exp arrhythmias, cardiac/ or cardiac arrhythmia*.ti,ab. or anemia/ or an?emia*.ti,ab. or obesity/ or (obesity or obese).ti,ab. or prostatic hyperplasia/ or (prostatic adj1 (hyperplasia or hypertrophy)).ti,ab. or tuberculosis/ or tuberculosis.ti,ab. or exp chronic hepatitis/ or chronic hepatitis.ti,ab. or substance-related disorders/ or (((substance or drug or marijuana or cocaine or amphetamine) adj2 abuse) or "substance abuse" or addiction?).ti,ab. or inflammatory bowel diseases/ or (inflammatory bowel disease? or ulcerative colitis or crohn or ibd).ti,ab. or deafness/ or deaf*.ti,ab. or blindness/ or (blind* or (visual adj1 loss)).ti,ab. or parkinson disease/ or parkinson*.ti,ab. or exp autism spectrum disorder/ or autis*.ti,ab.) (47467)

12 (exp dyslipidemias/ or (dyslipid?emia* or hyperlipid?emia* or hypercholesterolemia* or hypertriglyceridemia*).ti,ab.) and (exp thyroid diseases/ or ((thyroid adj1 (disease? or disorder?)) or hyperthyroid* or hypothyroid*).ti,ab. or joint diseases/ or osteoarthritis/ or arthritis rheumatoid/ or (joint disease* or rheumatoid arthritis or osteoarthritis).ti,ab. or exp kidney diseases/ or (kidney adj1 (disease? or disorder?)).ti,ab. or epilepsy/ or (epilep* or seizure?).ti,ab. or hiv infections/ or (hiv or acquired immun* deficiency syndrome? or aids).ti,ab. or exp liver diseases/ or (liver adj1 (disease? or disorder?)).ti,ab. or stroke/ or stroke*.ti,ab. or dementia/ or dementia.ti,ab. or exp arrhythmias, cardiac/ or cardiac arrhythmia*.ti,ab. or anemia/ or an?emia*.ti,ab. or obesity/ or (obesity or obese).ti,ab. or prostatic hyperplasia/ or (prostatic adj1 (hyperplasia or hypertrophy)).ti,ab. or tuberculosis/ or tuberculosis.ti,ab. or exp chronic hepatitis/ or chronic hepatitis.ti,ab. or substance-related disorders/ or (((substance or drug or marijuana or cocaine or amphetamine) adj2 abuse) or "substance abuse" or addiction?).ti,ab. or inflammatory bowel diseases/ or (inflammatory bowel disease? or ulcerative colitis or crohn or ibd).ti,ab. or deafness/ or deaf*.ti,ab. or blindness/ or (blind* or (visual adj1 loss)).ti,ab. or parkinson disease/ or parkinson*.ti,ab. or exp autism spectrum disorder/ or autis*.ti,ab.) (47467)

13 (joint diseases/ or osteoarthritis/ or arthritis rheumatoid/ or (joint disease* or rheumatoid arthritis or osteoarthritis).ti,ab.) and (exp kidney diseases/ or (kidney adj1 (disease? or disorder?)).ti,ab. or epilepsy/ or (epilep* or seizure?).ti,ab. or hiv infections/ or (hiv or acquired immun* deficiency syndrome? or aids).ti,ab. or exp liver diseases/ or (liver adj1 (disease? or disorder?)).ti,ab. or stroke/ or stroke*.ti,ab. or dementia/ or dementia.ti,ab. or exp arrhythmias, cardiac/ or cardiac arrhythmia*.ti,ab. or anemia/ or an?emia*.ti,ab. or obesity/ or (obesity or obese).ti,ab. or prostatic hyperplasia/ or (prostatic adj1 (hyperplasia or hypertrophy)).ti,ab. or tuberculosis/ or tuberculosis.ti,ab. or exp chronic hepatitis/ or chronic hepatitis.ti,ab. or substance-related disorders/ or (((substance or drug or marijuana or cocaine or amphetamine) adj2 abuse) or "substance abuse" or addiction?).ti,ab. or inflammatory bowel diseases/ or (inflammatory bowel disease? or ulcerative colitis or crohn or ibd).ti,ab. or deafness/ or deaf*.ti,ab. or blindness/ or (blind* or (visual adj1 loss)).ti,ab. or parkinson disease/ or parkinson*.ti,ab. or exp autism spectrum disorder/ or autis*.ti,ab.) (14003)

14 (exp kidney diseases/ or (kidney adj1 (disease? or disorder?)).ti,ab.) and (epilepsy/ or (epilep* or seizure?).ti,ab. or hiv infections/ or (hiv or acquired immun* deficiency syndrome? or aids).ti,ab. or exp liver diseases/ or (liver adj1 (disease? or disorder?)).ti,ab. or stroke/ or stroke*.ti,ab. or dementia/ or dementia.ti,ab. or exp arrhythmias, cardiac/ or cardiac arrhythmia*.ti,ab. or anemia/ or an?emia*.ti,ab. or obesity/ or (obesity or obese).ti,ab. or prostatic hyperplasia/ or (prostatic adj1 (hyperplasia or hypertrophy)).ti,ab. or tuberculosis/ or tuberculosis.ti,ab. or exp chronic hepatitis/ or chronic hepatitis.ti,ab. or substance-related disorders/ or (((substance or drug or marijuana or cocaine or amphetamine) adj2 abuse) or "substance abuse" or addiction?).ti,ab. or inflammatory bowel diseases/ or (inflammatory bowel disease? or ulcerative colitis or crohn or ibd).ti,ab. or deafness/ or deaf*.ti,ab. or blindness/ or (blind* or (visual adj1 loss)).ti,ab. or parkinson disease/ or parkinson*.ti,ab. or exp autism spectrum disorder/ or autis*.ti,ab.) (68076)

15 (epilepsy/ or (epilep* or seizure?).ti,ab.) and (hiv infections/ or (hiv or acquired immun* deficiency syndrome? or aids).ti,ab. or exp liver diseases/ or (liver adj1 (disease? or disorder?)).ti,ab. or stroke/ or stroke*.ti,ab. or dementia/ or dementia.ti,ab. or exp arrhythmias, cardiac/ or cardiac arrhythmia*.ti,ab. or anemia/ or an?emia*.ti,ab. or obesity/ or (obesity or obese).ti,ab. or prostatic hyperplasia/ or (prostatic adj1 (hyperplasia or hypertrophy)).ti,ab. or tuberculosis/ or tuberculosis.ti,ab. or exp chronic hepatitis/ or chronic hepatitis.ti,ab. or substance-related disorders/ or (((substance or drug or marijuana or cocaine or amphetamine) adj2 abuse) or "substance abuse" or addiction?).ti,ab. or inflammatory bowel diseases/ or (inflammatory bowel disease? or ulcerative colitis or crohn or ibd).ti,ab. or deafness/ or deaf*.ti,ab. or blindness/ or (blind* or (visual adj1 loss)).ti,ab. or parkinson disease/ or parkinson*.ti,ab. or exp autism spectrum disorder/ or autis*.ti,ab.) (25981)

16 (hiv infections/ or (hiv or acquired immun* deficiency syndrome? or aids).ti,ab.) and (exp liver diseases/ or (liver adj1 (disease? or disorder?)).ti,ab. or stroke/ or stroke*.ti,ab. or dementia/ or dementia.ti,ab. or exp arrhythmias, cardiac/ or cardiac arrhythmia*.ti,ab. or anemia/ or an?emia*.ti,ab. or obesity/ or (obesity or obese).ti,ab. or prostatic hyperplasia/ or (prostatic adj1 (hyperplasia or hypertrophy)).ti,ab. or tuberculosis/ or tuberculosis.ti,ab. or exp chronic hepatitis/ or chronic hepatitis.ti,ab. or substance-related disorders/ or (((substance or drug or marijuana or cocaine or amphetamine) adj2 abuse) or "substance abuse" or addiction?).ti,ab. or inflammatory bowel diseases/ or (inflammatory bowel disease? or ulcerative colitis or crohn or ibd).ti,ab. or deafness/ or deaf*.ti,ab. or blindness/ or (blind* or (visual adj1 loss)).ti,ab. or parkinson disease/ or parkinson*.ti,ab. or exp autism spectrum disorder/ or autis*.ti,ab.) (63446)

17 (exp liver diseases/ or (liver adj1 (disease? or disorder?)).ti,ab.) and (stroke/ or stroke*.ti,ab. or dementia/ or dementia.ti,ab. or exp arrhythmias, cardiac/ or cardiac arrhythmia*.ti,ab. or anemia/ or an?emia*.ti,ab. or obesity/ or (obesity or obese).ti,ab. or prostatic hyperplasia/ or (prostatic adj1 (hyperplasia or hypertrophy)).ti,ab. or tuberculosis/ or tuberculosis.ti,ab. or exp chronic hepatitis/ or chronic hepatitis.ti,ab. or substance-related disorders/ or (((substance or drug or marijuana or cocaine or amphetamine) adj2 abuse) or "substance abuse" or addiction?).ti,ab. or inflammatory bowel diseases/ or (inflammatory bowel disease? or ulcerative colitis or crohn or ibd).ti,ab. or deafness/ or deaf*.ti,ab. or blindness/ or (blind* or (visual adj1 loss)).ti,ab. or parkinson disease/ or parkinson*.ti,ab. or exp autism spectrum disorder/ or autis*.ti,ab.) (93443)

18 (stroke/ or stroke*.ti,ab.) and (dementia/ or dementia.ti,ab. or exp arrhythmias, cardiac/ or cardiac arrhythmia*.ti,ab. or anemia/ or an?emia*.ti,ab. or obesity/ or (obesity or obese).ti,ab. or prostatic hyperplasia/ or (prostatic adj1 (hyperplasia or hypertrophy)).ti,ab. or tuberculosis/ or tuberculosis.ti,ab. or exp chronic hepatitis/ or chronic hepatitis.ti,ab. or substance-related disorders/ or (((substance or drug or marijuana or cocaine or amphetamine) adj2 abuse) or "substance abuse" or addiction?).ti,ab. or inflammatory bowel diseases/ or (inflammatory bowel disease? or ulcerative colitis or crohn or ibd).ti,ab. or deafness/ or deaf*.ti,ab. or blindness/ or (blind* or (visual adj1 loss)).ti,ab. or parkinson disease/ or parkinson*.ti,ab. or exp autism spectrum disorder/ or autis*.ti,ab.) (38951)

19 (dementia/ or dementia.ti,ab.) and (exp arrhythmias, cardiac/ or cardiac arrhythmia*.ti,ab. or anemia/ or an?emia*.ti,ab. or obesity/ or (obesity or obese).ti,ab. or prostatic hyperplasia/ or (prostatic adj1 (hyperplasia or hypertrophy)).ti,ab. or tuberculosis/ or tuberculosis.ti,ab. or exp chronic hepatitis/ or chronic hepatitis.ti,ab. or Substance-Related Disorders/ or (((substance or drug or marijuana or cocaine or amphetamine) adj2 abuse) or "substance abuse" or addiction?).ti,ab. or Inflammatory Bowel Diseases/ or (Inflammatory Bowel Disease? or ulcerative colitis or crohn or IBD).ti,ab. or deafness/ or deaf*.ti,ab. or blindness/ or (blind* or (visual adj1 loss)).ti,ab. or Parkinson Disease/ or parkinson*.ti,ab. or exp Autism Spectrum Disorder/ or autis*.ti,ab.) (17065)

20 (exp arrhythmias, cardiac/ or cardiac arrhythmia*.ti,ab.) and (anemia/ or an?emia*.ti,ab. or obesity/ or (obesity or obese).ti,ab. or prostatic hyperplasia/ or (prostatic adj1 (hyperplasia or hypertrophy)).ti,ab. or tuberculosis/ or tuberculosis.ti,ab. or exp chronic hepatitis/ or chronic hepatitis.ti,ab. or substance-related disorders/ or (((substance or drug or marijuana or cocaine or amphetamine) adj2 abuse) or "substance abuse" or addiction?).ti,ab. or inflammatory bowel diseases/ or (inflammatory bowel disease? or ulcerative colitis or crohn or ibd).ti,ab. or deafness/ or deaf*.ti,ab. or blindness/ or (blind* or (visual adj1 loss)).ti,ab. or parkinson disease/ or parkinson*.ti,ab. or exp autism spectrum disorder/ or autis*.ti,ab.) (9962)

21 (anemia/ or an?emia*.ti,ab.) and (obesity/ or (obesity or obese).ti,ab. or prostatic hyperplasia/ or (prostatic adj1 (hyperplasia or hypertrophy)).ti,ab. or tuberculosis/ or tuberculosis.ti,ab. or exp chronic hepatitis/ or chronic hepatitis.ti,ab. or substance-related disorders/ or (((substance or drug or marijuana or cocaine or amphetamine) adj2 abuse) or "substance abuse" or addiction?).ti,ab. or inflammatory bowel diseases/ or (inflammatory bowel disease? or ulcerative colitis or crohn or ibd).ti,ab. or deafness/ or deaf*.ti,ab. or blindness/ or (blind* or (visual adj1 loss)).ti,ab. or parkinson disease/ or parkinson*.ti,ab. or exp autism spectrum disorder/ or autis*.ti,ab.) (7722)

22 (obesity/ or (obesity or obese).ti,ab.) and (prostatic hyperplasia/ or (prostatic adj1 (hyperplasia or hypertrophy)).ti,ab. or tuberculosis/ or tuberculosis.ti,ab. or exp chronic hepatitis/ or chronic hepatitis.ti,ab. or substance-related disorders/ or (((substance or drug or marijuana or cocaine or amphetamine) adj2 abuse) or "substance abuse" or addiction?).ti,ab. or inflammatory bowel diseases/ or (inflammatory bowel disease? or ulcerative colitis or crohn or ibd).ti,ab. or deafness/ or deaf*.ti,ab. or blindness/ or (blind* or (visual adj1 loss)).ti,ab. or parkinson disease/ or parkinson*.ti,ab. or exp autism spectrum disorder/ or autis*.ti,ab.) (9924)

23 (prostatic hyperplasia/ or (prostatic adj1 (hyperplasia or hypertrophy)).ti,ab.) and (tuberculosis/ or tuberculosis.ti,ab. or exp chronic hepatitis/ or chronic hepatitis.ti,ab. or substance-related disorders/ or (((substance or drug or marijuana or cocaine or amphetamine) adj2 abuse) or "substance abuse" or addiction?).ti,ab. or inflammatory bowel diseases/ or (inflammatory bowel disease? or ulcerative colitis or crohn or ibd).ti,ab. or deafness/ or deaf*.ti,ab. or blindness/ or (blind* or (visual adj1 loss)).ti,ab. or parkinson disease/ or parkinson*.ti,ab. or exp autism spectrum disorder/ or autis*.ti,ab.) (868)

24 (tuberculosis/ or tuberculosis.ti,ab.) and (exp chronic hepatitis/ or chronic hepatitis.ti,ab. or substance-related disorders/ or (((substance or drug or marijuana or cocaine or amphetamine) adj2 abuse) or "substance abuse" or addiction?).ti,ab. or inflammatory bowel diseases/ or (inflammatory bowel disease? or ulcerative colitis or crohn or ibd).ti,ab. or deafness/ or deaf*.ti,ab. or blindness/ or (blind* or (visual adj1 loss)).ti,ab. or parkinson disease/ or parkinson*.ti,ab. or exp autism spectrum disorder/ or autis*.ti,ab.) (2864)

25 (exp chronic hepatitis/ or chronic hepatitis.ti,ab.) and (substance-related disorders/ or (((substance or drug or marijuana or cocaine or amphetamine) adj2 abuse) or "substance abuse" or addiction?).ti,ab. or inflammatory bowel diseases/ or (inflammatory bowel disease? or ulcerative colitis or crohn or ibd).ti,ab. or deafness/ or deaf*.ti,ab. or blindness/ or (blind* or (visual adj1 loss)).ti,ab. or parkinson disease/ or parkinson*.ti,ab. or exp autism spectrum disorder/ or autis*.ti,ab.) (1760)

26 (substance-related disorders/ or (((substance or drug or marijuana or cocaine or amphetamine) adj2 abuse) or "substance abuse" or addiction?).ti,ab.) and (inflammatory bowel diseases/ or (inflammatory bowel disease? or ulcerative colitis or crohn or ibd).ti,ab. or deafness/ or deaf*.ti,ab. or blindness/ or (blind* or (visual adj1 loss)).ti,ab. or parkinson disease/ or parkinson*.ti,ab. or exp autism spectrum disorder/ or autis*.ti,ab.) (3179)

27 (inflammatory bowel diseases/ or (inflammatory bowel disease? or ulcerative colitis or crohn or ibd).ti,ab.) and (deafness/ or deaf*.ti,ab. or blindness/ or (blind* or (visual adj1 loss)).ti,ab. or parkinson disease/ or parkinson*.ti,ab. or exp autism spectrum disorder/ or autis*.ti,ab.) (1693)

28 (deafness/ or deaf*.ti,ab.) and (blindness/ or (blind* or (visual adj1 loss)).ti,ab. or parkinson disease/ or parkinson*.ti,ab. or exp autism spectrum disorder/ or autis*.ti,ab.) (2410)

29 (blindness/ or (blind* or (visual adj1 loss)).ti,ab.) and (parkinson disease/ or parkinson*.ti,ab. or exp autism spectrum disorder/ or autis*.ti,ab.) (3731)

30 (parkinson disease/ or parkinson*.ti,ab.) and (exp autism spectrum disorder/ or autis*.ti,ab.) (502)

31 exp comorbidity/ (109504)

32 (((condition* or diagnos* or disease* or illness* or health problem* or patholog* or disorder* or syndrome*) adj1 (associated or coexisting or co-existing or comorbid or co-morbid or concurrent or co-occuring or cooccuring or multiple)) or associated morbidit*).ti,ab. (93855)

33 (comorbidit* or co-morbidit* or multidisease* or multi-disease* or multimorbidit* or multi-morbidit* or multipatholog* or multi-patholog* or pluripatholog* or polypatholog* or poly-pathology*).ab,ti. (149457)

~~34 1 or 2 or 3 or 4 or 5 or 6 or 7 or 8 or 9 or 10 or 11 or 12 or 13 or 14 or 15 or 16 or 17 or 18 or 19 or 20 or 21 or 22 or 23 or 24 or 25 or 26 or 27 or 28 or 29 or 30 or 31 or 32 or 33 (6888816)~~

35 exp health care costs/ or exp health expenditures/ (84333)

36 cost*.mp. or ((health care or health-care or healthcare or medical or hospital) adj3 (expenditure or expense or spend or pay*)).ti,ab. [mp=title, abstract, original title, name of substance word, subject heading word, floating sub-heading word, keyword heading word, organism supplementary concept word, protocol supplementary concept word, rare disease supplementary concept word, unique identifier, synonyms] (721015)

37 (((resource or "health service") adj3 ("use" or utilisation or utilization)) or ((cost or financial or economic) and (burden or impact or consequence)) or out of pocket or out-of-pocket or oop or absenteeism or (productivity adj3 (loss or lost)) or economic model*).ab,ti. (145673)

~~38 35 or 36 or 37 (788846)~~

~~39 34 and 38 (197834)~~

~~40 limit 39 to yr="2010 -Current" (117873)~~

~~41 2 or 3 (710255)~~

42 1 or 2 or 3 or 4 or 5 or 6 or 7 or 8 or 9 or 10 or 11 or 12 or 13 or 14 or 15 or 16 or 17 or 18 or 19 or 20 or 21 or 22 or 23 or 24 or 25 or 26 or 27 or 28 or 29 or 30 (6754239)

43 31 or 32 or 33 (313659)

44 42 and 43 (179082)

45 44 and 38 (11185)

46 limit 45 to yr="2010 -Current" (8093)

***************************

Executed search Cochrane:

| 1. (pulmonary disease, chronic obstructive/ or (copd or (pulmonary adj2 (disease? or disorder?))).ti,ab.) and (diabetes mellitus/ or diabet*.ti,ab. or hypertension/ or (hypertens* or "high blood pressure?").ti,ab. or neoplasms/ or (neoplasm? or cancer?).ti,ab. or coronary disease/ or ((cardiac or cardiovascular or coronary) adj1 (disease? or disorder? or failure)).ti,ab. or exp myocardial infarction/ or (myocardial infarct* or cardiovascular strok*).ti,ab. or exp heart failure/ or ((heart or cardiac or myocardial) adj1 failure).ti,ab. or asthma/ or asthma*.ti,ab. or anxiety/ or depression/ or (((mental or anxiety or mood or psychological or sleep) adj1 (disease? or disorder?)) or depression?).ti,ab. or osteoporosis/ or osteoporosis.ti,ab. or exp dyslipidemias/ or (dyslipid?emia* or hyperlipid?emia* or hypercholesterolemia* or hypertriglyceridemia*).ti,ab. or exp thyroid diseases/ or ((thyroid adj1 (disease? or disorder?)) or hyperthyroid* or hypothyroid*).ti,ab. or joint diseases/ or osteoarthritis/ or arthritis rheumatoid/ or (joint disease* or rheumatoid arthritis or osteoarthritis).ti,ab. or exp kidney diseases/ or (kidney adj1 (disease? or disorder?)).ti,ab. or epilepsy/ or (epilep* or seizure?).ti,ab. or hiv infections/ or (hiv or acquired immun* deficiency syndrome? or aids).ti,ab. or exp liver diseases/ or (liver adj1 (disease? or disorder?)).ti,ab. or stroke/ or stroke*.ti,ab. or dementia/ or dementia.ti,ab. or exp arrhythmias, cardiac/ or cardiac arrhythmia*.ti,ab. or anemia/ or an?emia*.ti,ab. or obesity/ or (obesity or obese).ti,ab. or prostatic hyperplasia/ or (prostatic adj1 (hyperplasia or hypertrophy)).ti,ab. or tuberculosis/ or tuberculosis.ti,ab. or exp chronic hepatitis/ or chronic hepatitis.ti,ab. or substance-related disorders/ or (((substance or drug or marijuana or cocaine or amphetamine) adj2 abuse) or "substance abuse" or addiction?).ti,ab. or inflammatory bowel diseases/ or (inflammatory bowel disease? or ulcerative colitis or crohn or ibd).ti,ab. or deafness/ or deaf*.ti,ab. or blindness/ or (blind* or (visual adj1 loss)).ti,ab. or parkinson disease/ or parkinson*.ti,ab. or exp autism spectrum disorder/ or autis*.ti,ab.) |  |
| --- | --- |
| 2. (diabetes mellitus/ or diabet*.ti,ab.) and (hypertension/ or (hypertens* or "high blood pressure?").ti,ab. or neoplasms/ or (neoplasm? or cancer?).ti,ab. or coronary disease/ or ((cardiac or cardiovascular or coronary) adj1 (disease? or disorder? or failure)).ti,ab. or exp myocardial infarction/ or (myocardial infarct* or cardiovascular strok*).ti,ab. or exp heart failure/ or ((heart or cardiac or myocardial) adj1 failure).ti,ab. or asthma/ or asthma*.ti,ab. or anxiety/ or depression/ or (((mental or anxiety or mood or psychological or sleep) adj1 (disease? or disorder?)) or depression?).ti,ab. or osteoporosis/ or osteoporosis.ti,ab. or exp dyslipidemias/ or (dyslipid?emia* or hyperlipid?emia* or hypercholesterolemia* or hypertriglyceridemia*).ti,ab. or exp thyroid diseases/ or ((thyroid adj1 (disease? or disorder?)) or hyperthyroid* or hypothyroid*).ti,ab. or joint diseases/ or osteoarthritis/ or arthritis rheumatoid/ or (joint disease* or rheumatoid arthritis or osteoarthritis).ti,ab. or exp kidney diseases/ or (kidney adj1 (disease? or disorder?)).ti,ab. or epilepsy/ or (epilep* or seizure?).ti,ab. or hiv infections/ or (hiv or acquired immun* deficiency syndrome? or aids).ti,ab. or exp liver diseases/ or (liver adj1 (disease? or disorder?)).ti,ab. or stroke/ or stroke*.ti,ab. or dementia/ or dementia.ti,ab. or exp arrhythmias, cardiac/ or cardiac arrhythmia*.ti,ab. or anemia/ or an?emia*.ti,ab. or obesity/ or (obesity or obese).ti,ab. or prostatic hyperplasia/ or (prostatic adj1 (hyperplasia or hypertrophy)).ti,ab. or tuberculosis/ or tuberculosis.ti,ab. or exp chronic hepatitis/ or chronic hepatitis.ti,ab. or substance-related disorders/ or (((substance or drug or marijuana or cocaine or amphetamine) adj2 abuse) or "substance abuse" or addiction?).ti,ab. or inflammatory bowel diseases/ or (inflammatory bowel disease? or ulcerative colitis or crohn or ibd).ti,ab. or deafness/ or deaf*.ti,ab. or blindness/ or (blind* or (visual adj1 loss)).ti,ab. or parkinson disease/ or parkinson*.ti,ab. or exp autism spectrum disorder/ or autis*.ti,ab.) |  |
| 3. (neoplasms/ or (neoplasm? or cancer?).ti,ab.) and (coronary disease/ or ((cardiac or cardiovascular or coronary) adj1 (disease? or disorder? or failure)).ti,ab. or exp myocardial infarction/ or (myocardial infarct* or cardiovascular strok*).ti,ab. or exp heart failure/ or ((heart or cardiac or myocardial) adj1 failure).ti,ab. or asthma/ or asthma*.ti,ab. or anxiety/ or depression/ or (((mental or anxiety or mood or psychological or sleep) adj1 (disease? or disorder?)) or depression?).ti,ab. or osteoporosis/ or osteoporosis.ti,ab. or exp dyslipidemias/ or (dyslipid?emia* or hyperlipid?emia* or hypercholesterolemia* or hypertriglyceridemia*).ti,ab. or exp thyroid diseases/ or ((thyroid adj1 (disease? or disorder?)) or hyperthyroid* or hypothyroid*).ti,ab. or joint diseases/ or osteoarthritis/ or arthritis rheumatoid/ or (joint disease* or rheumatoid arthritis or osteoarthritis).ti,ab. or exp kidney diseases/ or (kidney adj1 (disease? or disorder?)).ti,ab. or epilepsy/ or (epilep* or seizure?).ti,ab. or hiv infections/ or (hiv or acquired immun* deficiency syndrome? or aids).ti,ab. or exp liver diseases/ or (liver adj1 (disease? or disorder?)).ti,ab. or stroke/ or stroke*.ti,ab. or dementia/ or dementia.ti,ab. or exp arrhythmias, cardiac/ or cardiac arrhythmia*.ti,ab. or anemia/ or an?emia*.ti,ab. or obesity/ or (obesity or obese).ti,ab. or prostatic hyperplasia/ or (prostatic adj1 (hyperplasia or hypertrophy)).ti,ab. or tuberculosis/ or tuberculosis.ti,ab. or exp chronic hepatitis/ or chronic hepatitis.ti,ab. or substance-related disorders/ or (((substance or drug or marijuana or cocaine or amphetamine) adj2 abuse) or "substance abuse" or addiction?).ti,ab. or inflammatory bowel diseases/ or (inflammatory bowel disease? or ulcerative colitis or crohn or ibd).ti,ab. or deafness/ or deaf*.ti,ab. or blindness/ or (blind* or (visual adj1 loss)).ti,ab. or parkinson disease/ or parkinson*.ti,ab. or exp autism spectrum disorder/ or autis*.ti,ab.) |  |
| 4. (hypertension/ or (hypertens* or "high blood pressure?").ti,ab.) and (neoplasms/ or (neoplasm? or cancer?).ti,ab. or coronary disease/ or ((cardiac or cardiovascular or coronary) adj1 (disease? or disorder? or failure)).ti,ab. or exp myocardial infarction/ or (myocardial infarct* or cardiovascular strok*).ti,ab. or exp heart failure/ or ((heart or cardiac or myocardial) adj1 failure).ti,ab. or asthma/ or asthma*.ti,ab. or anxiety/ or depression/ or (((mental or anxiety or mood or psychological or sleep) adj1 (disease? or disorder?)) or depression?).ti,ab. or osteoporosis/ or osteoporosis.ti,ab. or exp dyslipidemias/ or (dyslipid?emia* or hyperlipid?emia* or hypercholesterolemia* or hypertriglyceridemia*).ti,ab. or exp thyroid diseases/ or ((thyroid adj1 (disease? or disorder?)) or hyperthyroid* or hypothyroid*).ti,ab. or joint diseases/ or osteoarthritis/ or arthritis rheumatoid/ or (joint disease* or rheumatoid arthritis or osteoarthritis).ti,ab. or exp kidney diseases/ or (kidney adj1 (disease? or disorder?)).ti,ab. or epilepsy/ or (epilep* or seizure?).ti,ab. or hiv infections/ or (hiv or acquired immun* deficiency syndrome? or aids).ti,ab. or exp liver diseases/ or (liver adj1 (disease? or disorder?)).ti,ab. or stroke/ or stroke*.ti,ab. or dementia/ or dementia.ti,ab. or exp arrhythmias, cardiac/ or cardiac arrhythmia*.ti,ab. or anemia/ or an?emia*.ti,ab. or obesity/ or (obesity or obese).ti,ab. or prostatic hyperplasia/ or (prostatic adj1 (hyperplasia or hypertrophy)).ti,ab. or tuberculosis/ or tuberculosis.ti,ab. or exp chronic hepatitis/ or chronic hepatitis.ti,ab. or substance-related disorders/ or (((substance or drug or marijuana or cocaine or amphetamine) adj2 abuse) or "substance abuse" or addiction?).ti,ab. or inflammatory bowel diseases/ or (inflammatory bowel disease? or ulcerative colitis or crohn or ibd).ti,ab. or deafness/ or deaf*.ti,ab. or blindness/ or (blind* or (visual adj1 loss)).ti,ab. or parkinson disease/ or parkinson*.ti,ab. or exp autism spectrum disorder/ or autis*.ti,ab.) |  |
| 5. (coronary disease/ or ((cardiac or cardiovascular or coronary) adj1 (disease? or disorder? or failure)).ti,ab.) and (exp Myocardial Infarction/ or (Myocardial Infarct* or cardiovascular strok*).ti,ab. or exp heart failure/ or ((heart or cardiac or myocardial) adj1 failure).ti,ab. or asthma/ or asthma*.ti,ab. or anxiety/ or depression/ or (((mental or anxiety or mood or psychological or sleep) adj1 (disease? or disorder?)) or depression?).ti,ab. or osteoporosis/ or osteoporosis.ti,ab. or exp dyslipidemias/ or (dyslipid?emia* or hyperlipid?emia* or hypercholesterolemia* or hypertriglyceridemia*).ti,ab. or exp thyroid diseases/ or ((thyroid adj1 (disease? or disorder?)) or hyperthyroid* or hypothyroid*).ti,ab. or joint diseases/ or osteoarthritis/ or arthritis rheumatoid/ or (joint disease* or rheumatoid arthritis or osteoarthritis).ti,ab. or exp kidney diseases/ or (kidney adj1 (disease? or disorder?)).ti,ab. or epilepsy/ or (epilep* or seizure?).ti,ab. or hiv infections/ or (HIV or acquired immun* deficiency syndrome? or aids).ti,ab. or exp liver diseases/ or (liver adj1 (disease? or disorder?)).ti,ab. or stroke/ or stroke*.ti,ab. or dementia/ or dementia.ti,ab. or exp arrhythmias, cardiac/ or cardiac arrhythmia*.ti,ab. or anemia/ or an?emia*.ti,ab. or obesity/ or (obesity or obese).ti,ab. or prostatic hyperplasia/ or (prostatic adj1 (hyperplasia or hypertrophy)).ti,ab. or tuberculosis/ or tuberculosis.ti,ab. or exp chronic hepatitis/ or chronic hepatitis.ti,ab. or Substance-Related Disorders/ or (((substance or drug or marijuana or cocaine or amphetamine) adj2 abuse) or "substance abuse" or addiction?).ti,ab. or Inflammatory Bowel Diseases/ or (Inflammatory Bowel Disease? or ulcerative colitis or crohn or IBD).ti,ab. or deafness/ or deaf*.ti,ab. or blindness/ or (blind* or (visual adj1 loss)).ti,ab. or Parkinson Disease/ or parkinson*.ti,ab. or exp Autism Spectrum Disorder/ or autis*.ti,ab.) |  |
| 6. (exp myocardial infarction/ or (myocardial infarct* or cardiovascular strok*).ti,ab.) and (exp heart failure/ or ((heart or cardiac or myocardial) adj1 failure).ti,ab. or asthma/ or asthma*.ti,ab. or anxiety/ or depression/ or (((mental or anxiety or mood or psychological or sleep) adj1 (disease? or disorder?)) or depression?).ti,ab. or osteoporosis/ or osteoporosis.ti,ab. or exp dyslipidemias/ or (dyslipid?emia* or hyperlipid?emia* or hypercholesterolemia* or hypertriglyceridemia*).ti,ab. or exp thyroid diseases/ or ((thyroid adj1 (disease? or disorder?)) or hyperthyroid* or hypothyroid*).ti,ab. or joint diseases/ or osteoarthritis/ or arthritis rheumatoid/ or (joint disease* or rheumatoid arthritis or osteoarthritis).ti,ab. or exp kidney diseases/ or (kidney adj1 (disease? or disorder?)).ti,ab. or epilepsy/ or (epilep* or seizure?).ti,ab. or hiv infections/ or (hiv or acquired immun* deficiency syndrome? or aids).ti,ab. or exp liver diseases/ or (liver adj1 (disease? or disorder?)).ti,ab. or stroke/ or stroke*.ti,ab. or dementia/ or dementia.ti,ab. or exp arrhythmias, cardiac/ or cardiac arrhythmia*.ti,ab. or anemia/ or an?emia*.ti,ab. or obesity/ or (obesity or obese).ti,ab. or prostatic hyperplasia/ or (prostatic adj1 (hyperplasia or hypertrophy)).ti,ab. or tuberculosis/ or tuberculosis.ti,ab. or exp chronic hepatitis/ or chronic hepatitis.ti,ab. or substance-related disorders/ or (((substance or drug or marijuana or cocaine or amphetamine) adj2 abuse) or "substance abuse" or addiction?).ti,ab. or inflammatory bowel diseases/ or (inflammatory bowel disease? or ulcerative colitis or crohn or ibd).ti,ab. or deafness/ or deaf*.ti,ab. or blindness/ or (blind* or (visual adj1 loss)).ti,ab. or parkinson disease/ or parkinson*.ti,ab. or exp autism spectrum disorder/ or autis*.ti,ab.) |  |
| 7. (exp heart failure/ or ((heart or cardiac or myocardial) adj1 failure).ti,ab.) and (asthma/ or asthma*.ti,ab. or anxiety/ or depression/ or (((mental or anxiety or mood or psychological or sleep) adj1 (disease? or disorder?)) or depression?).ti,ab. or osteoporosis/ or osteoporosis.ti,ab. or exp dyslipidemias/ or (dyslipid?emia* or hyperlipid?emia* or hypercholesterolemia* or hypertriglyceridemia*).ti,ab. or exp thyroid diseases/ or ((thyroid adj1 (disease? or disorder?)) or hyperthyroid* or hypothyroid*).ti,ab. or joint diseases/ or osteoarthritis/ or arthritis rheumatoid/ or (joint disease* or rheumatoid arthritis or osteoarthritis).ti,ab. or exp kidney diseases/ or (kidney adj1 (disease? or disorder?)).ti,ab. or epilepsy/ or (epilep* or seizure?).ti,ab. or hiv infections/ or (hiv or acquired immun* deficiency syndrome? or aids).ti,ab. or exp liver diseases/ or (liver adj1 (disease? or disorder?)).ti,ab. or stroke/ or stroke*.ti,ab. or dementia/ or dementia.ti,ab. or exp arrhythmias, cardiac/ or cardiac arrhythmia*.ti,ab. or anemia/ or an?emia*.ti,ab. or obesity/ or (obesity or obese).ti,ab. or prostatic hyperplasia/ or (prostatic adj1 (hyperplasia or hypertrophy)).ti,ab. or tuberculosis/ or tuberculosis.ti,ab. or exp chronic hepatitis/ or chronic hepatitis.ti,ab. or substance-related disorders/ or (((substance or drug or marijuana or cocaine or amphetamine) adj2 abuse) or "substance abuse" or addiction?).ti,ab. or inflammatory bowel diseases/ or (inflammatory bowel disease? or ulcerative colitis or crohn or ibd).ti,ab. or deafness/ or deaf*.ti,ab. or blindness/ or (blind* or (visual adj1 loss)).ti,ab. or parkinson disease/ or parkinson*.ti,ab. or exp autism spectrum disorder/ or autis*.ti,ab.) |  |
| 8. (asthma/ or asthma*.ti,ab.) and (anxiety/ or depression/ or (((mental or anxiety or mood or psychological or sleep) adj1 (disease? or disorder?)) or depression?).ti,ab. or osteoporosis/ or osteoporosis.ti,ab. or exp dyslipidemias/ or (dyslipid?emia* or hyperlipid?emia* or hypercholesterolemia* or hypertriglyceridemia*).ti,ab. or exp thyroid diseases/ or ((thyroid adj1 (disease? or disorder?)) or hyperthyroid* or hypothyroid*).ti,ab. or joint diseases/ or osteoarthritis/ or arthritis rheumatoid/ or (joint disease* or rheumatoid arthritis or osteoarthritis).ti,ab. or exp kidney diseases/ or (kidney adj1 (disease? or disorder?)).ti,ab. or epilepsy/ or (epilep* or seizure?).ti,ab. or hiv infections/ or (hiv or acquired immun* deficiency syndrome? or aids).ti,ab. or exp liver diseases/ or (liver adj1 (disease? or disorder?)).ti,ab. or stroke/ or stroke*.ti,ab. or dementia/ or dementia.ti,ab. or exp arrhythmias, cardiac/ or cardiac arrhythmia*.ti,ab. or anemia/ or an?emia*.ti,ab. or obesity/ or (obesity or obese).ti,ab. or prostatic hyperplasia/ or (prostatic adj1 (hyperplasia or hypertrophy)).ti,ab. or tuberculosis/ or tuberculosis.ti,ab. or exp chronic hepatitis/ or chronic hepatitis.ti,ab. or substance-related disorders/ or (((substance or drug or marijuana or cocaine or amphetamine) adj2 abuse) or "substance abuse" or addiction?).ti,ab. or inflammatory bowel diseases/ or (inflammatory bowel disease? or ulcerative colitis or crohn or ibd).ti,ab. or deafness/ or deaf*.ti,ab. or blindness/ or (blind* or (visual adj1 loss)).ti,ab. or parkinson disease/ or parkinson*.ti,ab. or exp autism spectrum disorder/ or autis*.ti,ab.) |  |
| 9. (anxiety/ or depression/ or (((mental or anxiety or mood or psychological or sleep) adj1 (disease? or disorder?)) or depression?).ti,ab.) and (osteoporosis/ or osteoporosis.ti,ab. or exp dyslipidemias/ or (dyslipid?emia* or hyperlipid?emia* or hypercholesterolemia* or hypertriglyceridemia*).ti,ab. or exp thyroid diseases/ or ((thyroid adj1 (disease? or disorder?)) or hyperthyroid* or hypothyroid*).ti,ab. or joint diseases/ or osteoarthritis/ or arthritis rheumatoid/ or (joint disease* or rheumatoid arthritis or osteoarthritis).ti,ab. or exp kidney diseases/ or (kidney adj1 (disease? or disorder?)).ti,ab. or epilepsy/ or (epilep* or seizure?).ti,ab. or hiv infections/ or (hiv or acquired immun* deficiency syndrome? or aids).ti,ab. or exp liver diseases/ or (liver adj1 (disease? or disorder?)).ti,ab. or stroke/ or stroke*.ti,ab. or dementia/ or dementia.ti,ab. or exp arrhythmias, cardiac/ or cardiac arrhythmia*.ti,ab. or anemia/ or an?emia*.ti,ab. or obesity/ or (obesity or obese).ti,ab. or prostatic hyperplasia/ or (prostatic adj1 (hyperplasia or hypertrophy)).ti,ab. or tuberculosis/ or tuberculosis.ti,ab. or exp chronic hepatitis/ or chronic hepatitis.ti,ab. or substance-related disorders/ or (((substance or drug or marijuana or cocaine or amphetamine) adj2 abuse) or "substance abuse" or addiction?).ti,ab. or inflammatory bowel diseases/ or (inflammatory bowel disease? or ulcerative colitis or crohn or ibd).ti,ab. or deafness/ or deaf*.ti,ab. or blindness/ or (blind* or (visual adj1 loss)).ti,ab. or parkinson disease/ or parkinson*.ti,ab. or exp autism spectrum disorder/ or autis*.ti,ab.) |  |
| 10. (osteoporosis/ or osteoporosis.ti,ab.) and (exp dyslipidemias/ or (dyslipid?emia* or hyperlipid?emia* or hypercholesterolemia* or hypertriglyceridemia*).ti,ab. or exp thyroid diseases/ or ((thyroid adj1 (disease? or disorder?)) or hyperthyroid* or hypothyroid*).ti,ab. or joint diseases/ or osteoarthritis/ or arthritis rheumatoid/ or (joint disease* or rheumatoid arthritis or osteoarthritis).ti,ab. or exp kidney diseases/ or (kidney adj1 (disease? or disorder?)).ti,ab. or epilepsy/ or (epilep* or seizure?).ti,ab. or hiv infections/ or (hiv or acquired immun* deficiency syndrome? or aids).ti,ab. or exp liver diseases/ or (liver adj1 (disease? or disorder?)).ti,ab. or stroke/ or stroke*.ti,ab. or dementia/ or dementia.ti,ab. or exp arrhythmias, cardiac/ or cardiac arrhythmia*.ti,ab. or anemia/ or an?emia*.ti,ab. or obesity/ or (obesity or obese).ti,ab. or prostatic hyperplasia/ or (prostatic adj1 (hyperplasia or hypertrophy)).ti,ab. or tuberculosis/ or tuberculosis.ti,ab. or exp chronic hepatitis/ or chronic hepatitis.ti,ab. or substance-related disorders/ or (((substance or drug or marijuana or cocaine or amphetamine) adj2 abuse) or "substance abuse" or addiction?).ti,ab. or inflammatory bowel diseases/ or (inflammatory bowel disease? or ulcerative colitis or crohn or ibd).ti,ab. or deafness/ or deaf*.ti,ab. or blindness/ or (blind* or (visual adj1 loss)).ti,ab. or parkinson disease/ or parkinson*.ti,ab. or exp autism spectrum disorder/ or autis*.ti,ab.) |  |
| 11. (exp dyslipidemias/ or (dyslipid?emia* or hyperlipid?emia* or hypercholesterolemia* or hypertriglyceridemia*).ti,ab.) and (exp thyroid diseases/ or ((thyroid adj1 (disease? or disorder?)) or hyperthyroid* or hypothyroid*).ti,ab. or joint diseases/ or osteoarthritis/ or arthritis rheumatoid/ or (joint disease* or rheumatoid arthritis or osteoarthritis).ti,ab. or exp kidney diseases/ or (kidney adj1 (disease? or disorder?)).ti,ab. or epilepsy/ or (epilep* or seizure?).ti,ab. or hiv infections/ or (hiv or acquired immun* deficiency syndrome? or aids).ti,ab. or exp liver diseases/ or (liver adj1 (disease? or disorder?)).ti,ab. or stroke/ or stroke*.ti,ab. or dementia/ or dementia.ti,ab. or exp arrhythmias, cardiac/ or cardiac arrhythmia*.ti,ab. or anemia/ or an?emia*.ti,ab. or obesity/ or (obesity or obese).ti,ab. or prostatic hyperplasia/ or (prostatic adj1 (hyperplasia or hypertrophy)).ti,ab. or tuberculosis/ or tuberculosis.ti,ab. or exp chronic hepatitis/ or chronic hepatitis.ti,ab. or substance-related disorders/ or (((substance or drug or marijuana or cocaine or amphetamine) adj2 abuse) or "substance abuse" or addiction?).ti,ab. or inflammatory bowel diseases/ or (inflammatory bowel disease? or ulcerative colitis or crohn or ibd).ti,ab. or deafness/ or deaf*.ti,ab. or blindness/ or (blind* or (visual adj1 loss)).ti,ab. or parkinson disease/ or parkinson*.ti,ab. or exp autism spectrum disorder/ or autis*.ti,ab.) |  |
| 12. (exp dyslipidemias/ or (dyslipid?emia* or hyperlipid?emia* or hypercholesterolemia* or hypertriglyceridemia*).ti,ab.) and (exp thyroid diseases/ or ((thyroid adj1 (disease? or disorder?)) or hyperthyroid* or hypothyroid*).ti,ab. or joint diseases/ or osteoarthritis/ or arthritis rheumatoid/ or (joint disease* or rheumatoid arthritis or osteoarthritis).ti,ab. or exp kidney diseases/ or (kidney adj1 (disease? or disorder?)).ti,ab. or epilepsy/ or (epilep* or seizure?).ti,ab. or hiv infections/ or (hiv or acquired immun* deficiency syndrome? or aids).ti,ab. or exp liver diseases/ or (liver adj1 (disease? or disorder?)).ti,ab. or stroke/ or stroke*.ti,ab. or dementia/ or dementia.ti,ab. or exp arrhythmias, cardiac/ or cardiac arrhythmia*.ti,ab. or anemia/ or an?emia*.ti,ab. or obesity/ or (obesity or obese).ti,ab. or prostatic hyperplasia/ or (prostatic adj1 (hyperplasia or hypertrophy)).ti,ab. or tuberculosis/ or tuberculosis.ti,ab. or exp chronic hepatitis/ or chronic hepatitis.ti,ab. or substance-related disorders/ or (((substance or drug or marijuana or cocaine or amphetamine) adj2 abuse) or "substance abuse" or addiction?).ti,ab. or inflammatory bowel diseases/ or (inflammatory bowel disease? or ulcerative colitis or crohn or ibd).ti,ab. or deafness/ or deaf*.ti,ab. or blindness/ or (blind* or (visual adj1 loss)).ti,ab. or parkinson disease/ or parkinson*.ti,ab. or exp autism spectrum disorder/ or autis*.ti,ab.) |  |
| 13. (joint diseases/ or osteoarthritis/ or arthritis rheumatoid/ or (joint disease* or rheumatoid arthritis or osteoarthritis).ti,ab.) and (exp kidney diseases/ or (kidney adj1 (disease? or disorder?)).ti,ab. or epilepsy/ or (epilep* or seizure?).ti,ab. or hiv infections/ or (hiv or acquired immun* deficiency syndrome? or aids).ti,ab. or exp liver diseases/ or (liver adj1 (disease? or disorder?)).ti,ab. or stroke/ or stroke*.ti,ab. or dementia/ or dementia.ti,ab. or exp arrhythmias, cardiac/ or cardiac arrhythmia*.ti,ab. or anemia/ or an?emia*.ti,ab. or obesity/ or (obesity or obese).ti,ab. or prostatic hyperplasia/ or (prostatic adj1 (hyperplasia or hypertrophy)).ti,ab. or tuberculosis/ or tuberculosis.ti,ab. or exp chronic hepatitis/ or chronic hepatitis.ti,ab. or substance-related disorders/ or (((substance or drug or marijuana or cocaine or amphetamine) adj2 abuse) or "substance abuse" or addiction?).ti,ab. or inflammatory bowel diseases/ or (inflammatory bowel disease? or ulcerative colitis or crohn or ibd).ti,ab. or deafness/ or deaf*.ti,ab. or blindness/ or (blind* or (visual adj1 loss)).ti,ab. or parkinson disease/ or parkinson*.ti,ab. or exp autism spectrum disorder/ or autis*.ti,ab.) |  |
| 14. (exp kidney diseases/ or (kidney adj1 (disease? or disorder?)).ti,ab.) and (epilepsy/ or (epilep* or seizure?).ti,ab. or hiv infections/ or (hiv or acquired immun* deficiency syndrome? or aids).ti,ab. or exp liver diseases/ or (liver adj1 (disease? or disorder?)).ti,ab. or stroke/ or stroke*.ti,ab. or dementia/ or dementia.ti,ab. or exp arrhythmias, cardiac/ or cardiac arrhythmia*.ti,ab. or anemia/ or an?emia*.ti,ab. or obesity/ or (obesity or obese).ti,ab. or prostatic hyperplasia/ or (prostatic adj1 (hyperplasia or hypertrophy)).ti,ab. or tuberculosis/ or tuberculosis.ti,ab. or exp chronic hepatitis/ or chronic hepatitis.ti,ab. or substance-related disorders/ or (((substance or drug or marijuana or cocaine or amphetamine) adj2 abuse) or "substance abuse" or addiction?).ti,ab. or inflammatory bowel diseases/ or (inflammatory bowel disease? or ulcerative colitis or crohn or ibd).ti,ab. or deafness/ or deaf*.ti,ab. or blindness/ or (blind* or (visual adj1 loss)).ti,ab. or parkinson disease/ or parkinson*.ti,ab. or exp autism spectrum disorder/ or autis*.ti,ab.) |  |
| 15. (epilepsy/ or (epilep* or seizure?).ti,ab.) and (hiv infections/ or (hiv or acquired immun* deficiency syndrome? or aids).ti,ab. or exp liver diseases/ or (liver adj1 (disease? or disorder?)).ti,ab. or stroke/ or stroke*.ti,ab. or dementia/ or dementia.ti,ab. or exp arrhythmias, cardiac/ or cardiac arrhythmia*.ti,ab. or anemia/ or an?emia*.ti,ab. or obesity/ or (obesity or obese).ti,ab. or prostatic hyperplasia/ or (prostatic adj1 (hyperplasia or hypertrophy)).ti,ab. or tuberculosis/ or tuberculosis.ti,ab. or exp chronic hepatitis/ or chronic hepatitis.ti,ab. or substance-related disorders/ or (((substance or drug or marijuana or cocaine or amphetamine) adj2 abuse) or "substance abuse" or addiction?).ti,ab. or inflammatory bowel diseases/ or (inflammatory bowel disease? or ulcerative colitis or crohn or ibd).ti,ab. or deafness/ or deaf*.ti,ab. or blindness/ or (blind* or (visual adj1 loss)).ti,ab. or parkinson disease/ or parkinson*.ti,ab. or exp autism spectrum disorder/ or autis*.ti,ab.) |  |
| 16. (hiv infections/ or (hiv or acquired immun* deficiency syndrome? or aids).ti,ab.) and (exp liver diseases/ or (liver adj1 (disease? or disorder?)).ti,ab. or stroke/ or stroke*.ti,ab. or dementia/ or dementia.ti,ab. or exp arrhythmias, cardiac/ or cardiac arrhythmia*.ti,ab. or anemia/ or an?emia*.ti,ab. or obesity/ or (obesity or obese).ti,ab. or prostatic hyperplasia/ or (prostatic adj1 (hyperplasia or hypertrophy)).ti,ab. or tuberculosis/ or tuberculosis.ti,ab. or exp chronic hepatitis/ or chronic hepatitis.ti,ab. or substance-related disorders/ or (((substance or drug or marijuana or cocaine or amphetamine) adj2 abuse) or "substance abuse" or addiction?).ti,ab. or inflammatory bowel diseases/ or (inflammatory bowel disease? or ulcerative colitis or crohn or ibd).ti,ab. or deafness/ or deaf*.ti,ab. or blindness/ or (blind* or (visual adj1 loss)).ti,ab. or parkinson disease/ or parkinson*.ti,ab. or exp autism spectrum disorder/ or autis*.ti,ab.) |  |
| 17. (exp liver diseases/ or (liver adj1 (disease? or disorder?)).ti,ab.) and (stroke/ or stroke*.ti,ab. or dementia/ or dementia.ti,ab. or exp arrhythmias, cardiac/ or cardiac arrhythmia*.ti,ab. or anemia/ or an?emia*.ti,ab. or obesity/ or (obesity or obese).ti,ab. or prostatic hyperplasia/ or (prostatic adj1 (hyperplasia or hypertrophy)).ti,ab. or tuberculosis/ or tuberculosis.ti,ab. or exp chronic hepatitis/ or chronic hepatitis.ti,ab. or substance-related disorders/ or (((substance or drug or marijuana or cocaine or amphetamine) adj2 abuse) or "substance abuse" or addiction?).ti,ab. or inflammatory bowel diseases/ or (inflammatory bowel disease? or ulcerative colitis or crohn or ibd).ti,ab. or deafness/ or deaf*.ti,ab. or blindness/ or (blind* or (visual adj1 loss)).ti,ab. or parkinson disease/ or parkinson*.ti,ab. or exp autism spectrum disorder/ or autis*.ti,ab.) |  |
| 18. (stroke/ or stroke*.ti,ab.) and (dementia/ or dementia.ti,ab. or exp arrhythmias, cardiac/ or cardiac arrhythmia*.ti,ab. or anemia/ or an?emia*.ti,ab. or obesity/ or (obesity or obese).ti,ab. or prostatic hyperplasia/ or (prostatic adj1 (hyperplasia or hypertrophy)).ti,ab. or tuberculosis/ or tuberculosis.ti,ab. or exp chronic hepatitis/ or chronic hepatitis.ti,ab. or substance-related disorders/ or (((substance or drug or marijuana or cocaine or amphetamine) adj2 abuse) or "substance abuse" or addiction?).ti,ab. or inflammatory bowel diseases/ or (inflammatory bowel disease? or ulcerative colitis or crohn or ibd).ti,ab. or deafness/ or deaf*.ti,ab. or blindness/ or (blind* or (visual adj1 loss)).ti,ab. or parkinson disease/ or parkinson*.ti,ab. or exp autism spectrum disorder/ or autis*.ti,ab.) |  |
| 19. (dementia/ or dementia.ti,ab.) and (exp arrhythmias, cardiac/ or cardiac arrhythmia*.ti,ab. or anemia/ or an?emia*.ti,ab. or obesity/ or (obesity or obese).ti,ab. or prostatic hyperplasia/ or (prostatic adj1 (hyperplasia or hypertrophy)).ti,ab. or tuberculosis/ or tuberculosis.ti,ab. or exp chronic hepatitis/ or chronic hepatitis.ti,ab. or Substance-Related Disorders/ or (((substance or drug or marijuana or cocaine or amphetamine) adj2 abuse) or "substance abuse" or addiction?).ti,ab. or Inflammatory Bowel Diseases/ or (Inflammatory Bowel Disease? or ulcerative colitis or crohn or IBD).ti,ab. or deafness/ or deaf*.ti,ab. or blindness/ or (blind* or (visual adj1 loss)).ti,ab. or Parkinson Disease/ or parkinson*.ti,ab. or exp Autism Spectrum Disorder/ or autis*.ti,ab.) |  |
| 20. (exp arrhythmias, cardiac/ or cardiac arrhythmia*.ti,ab.) and (anemia/ or an?emia*.ti,ab. or obesity/ or (obesity or obese).ti,ab. or prostatic hyperplasia/ or (prostatic adj1 (hyperplasia or hypertrophy)).ti,ab. or tuberculosis/ or tuberculosis.ti,ab. or exp chronic hepatitis/ or chronic hepatitis.ti,ab. or substance-related disorders/ or (((substance or drug or marijuana or cocaine or amphetamine) adj2 abuse) or "substance abuse" or addiction?).ti,ab. or inflammatory bowel diseases/ or (inflammatory bowel disease? or ulcerative colitis or crohn or ibd).ti,ab. or deafness/ or deaf*.ti,ab. or blindness/ or (blind* or (visual adj1 loss)).ti,ab. or parkinson disease/ or parkinson*.ti,ab. or exp autism spectrum disorder/ or autis*.ti,ab.) |  |
| 21. (anemia/ or an?emia*.ti,ab.) and (obesity/ or (obesity or obese).ti,ab. or prostatic hyperplasia/ or (prostatic adj1 (hyperplasia or hypertrophy)).ti,ab. or tuberculosis/ or tuberculosis.ti,ab. or exp chronic hepatitis/ or chronic hepatitis.ti,ab. or substance-related disorders/ or (((substance or drug or marijuana or cocaine or amphetamine) adj2 abuse) or "substance abuse" or addiction?).ti,ab. or inflammatory bowel diseases/ or (inflammatory bowel disease? or ulcerative colitis or crohn or ibd).ti,ab. or deafness/ or deaf*.ti,ab. or blindness/ or (blind* or (visual adj1 loss)).ti,ab. or parkinson disease/ or parkinson*.ti,ab. or exp autism spectrum disorder/ or autis*.ti,ab.) |  |
| 22. (obesity/ or (obesity or obese).ti,ab.) and (prostatic hyperplasia/ or (prostatic adj1 (hyperplasia or hypertrophy)).ti,ab. or tuberculosis/ or tuberculosis.ti,ab. or exp chronic hepatitis/ or chronic hepatitis.ti,ab. or substance-related disorders/ or (((substance or drug or marijuana or cocaine or amphetamine) adj2 abuse) or "substance abuse" or addiction?).ti,ab. or inflammatory bowel diseases/ or (inflammatory bowel disease? or ulcerative colitis or crohn or ibd).ti,ab. or deafness/ or deaf*.ti,ab. or blindness/ or (blind* or (visual adj1 loss)).ti,ab. or parkinson disease/ or parkinson*.ti,ab. or exp autism spectrum disorder/ or autis*.ti,ab.) |  |
| 23. (prostatic hyperplasia/ or (prostatic adj1 (hyperplasia or hypertrophy)).ti,ab.) and (tuberculosis/ or tuberculosis.ti,ab. or exp chronic hepatitis/ or chronic hepatitis.ti,ab. or substance-related disorders/ or (((substance or drug or marijuana or cocaine or amphetamine) adj2 abuse) or "substance abuse" or addiction?).ti,ab. or inflammatory bowel diseases/ or (inflammatory bowel disease? or ulcerative colitis or crohn or ibd).ti,ab. or deafness/ or deaf*.ti,ab. or blindness/ or (blind* or (visual adj1 loss)).ti,ab. or parkinson disease/ or parkinson*.ti,ab. or exp autism spectrum disorder/ or autis*.ti,ab.) |  |
| 24. (tuberculosis/ or tuberculosis.ti,ab.) and (exp chronic hepatitis/ or chronic hepatitis.ti,ab. or substance-related disorders/ or (((substance or drug or marijuana or cocaine or amphetamine) adj2 abuse) or "substance abuse" or addiction?).ti,ab. or inflammatory bowel diseases/ or (inflammatory bowel disease? or ulcerative colitis or crohn or ibd).ti,ab. or deafness/ or deaf*.ti,ab. or blindness/ or (blind* or (visual adj1 loss)).ti,ab. or parkinson disease/ or parkinson*.ti,ab. or exp autism spectrum disorder/ or autis*.ti,ab.) |  |
| 25. (exp chronic hepatitis/ or chronic hepatitis.ti,ab.) and (substance-related disorders/ or (((substance or drug or marijuana or cocaine or amphetamine) adj2 abuse) or "substance abuse" or addiction?).ti,ab. or inflammatory bowel diseases/ or (inflammatory bowel disease? or ulcerative colitis or crohn or ibd).ti,ab. or deafness/ or deaf*.ti,ab. or blindness/ or (blind* or (visual adj1 loss)).ti,ab. or parkinson disease/ or parkinson*.ti,ab. or exp autism spectrum disorder/ or autis*.ti,ab.) |  |
| 26. (substance-related disorders/ or (((substance or drug or marijuana or cocaine or amphetamine) adj2 abuse) or "substance abuse" or addiction?).ti,ab.) and (inflammatory bowel diseases/ or (inflammatory bowel disease? or ulcerative colitis or crohn or ibd).ti,ab. or deafness/ or deaf*.ti,ab. or blindness/ or (blind* or (visual adj1 loss)).ti,ab. or parkinson disease/ or parkinson*.ti,ab. or exp autism spectrum disorder/ or autis*.ti,ab.) |  |
| 27. (inflammatory bowel diseases/ or (inflammatory bowel disease? or ulcerative colitis or crohn or ibd).ti,ab.) and (deafness/ or deaf*.ti,ab. or blindness/ or (blind* or (visual adj1 loss)).ti,ab. or parkinson disease/ or parkinson*.ti,ab. or exp autism spectrum disorder/ or autis*.ti,ab.) |  |
| 28. (deafness/ or deaf*.ti,ab.) and (blindness/ or (blind* or (visual adj1 loss)).ti,ab. or parkinson disease/ or parkinson*.ti,ab. or exp autism spectrum disorder/ or autis*.ti,ab.) |  |
| 29. (blindness/ or (blind* or (visual adj1 loss)).ti,ab.) and (parkinson disease/ or parkinson*.ti,ab. or exp autism spectrum disorder/ or autis*.ti,ab.) |  |
| 30. (parkinson disease/ or parkinson*.ti,ab.) and (exp autism spectrum disorder/ or autis*.ti,ab.) |  |
| 31. 1 or 2 or 3 or 4 or 5 or 6 or 7 or 8 or 9 or 10 or 11 or 12 or 13 or 14 or 15 or 16 or 17 or 18 or 19 or 20 or 21 or 22 or 23 or 24 or 25 or 26 or 27 or 28 or 29 or 30 |  |
| 32. exp comorbidity/ |  |
| 33. (((condition* or diagnos* or disease* or illness* or health problem* or patholog* or disorder* or syndrome*) adj1 (associated or coexisting or co-existing or comorbid or co-morbid or concurrent or co-occuring or cooccuring or multiple)) or associated morbidit*).ti,ab. |  |
| 34. (comorbidit* or co-morbidit* or multidisease* or multi-disease* or multimorbidit* or multi-morbidit* or multipatholog* or multi-patholog* or pluripatholog* or polypatholog* or poly-pathology*).ab,ti. |  |
| 35. 32 or 33 or 34 |  |
| 36. exp health care costs/ or exp health expenditures/ |  |
| 37. cost*.mp. or ((health care or health-care or healthcare or medical or hospital) adj3 (expenditure or expense or spend or pay*)).ti,ab. |  |
| 38. (((resource or "health service") adj3 (utilisation or utilization)) or ((cost or financial or economic) and (burden or impact or consequence)) or out of pocket or out-of-pocket or oop or absenteeism or (productivity adj3 (loss or lost)) or economic model*).ab,ti. |  |
| 39. 36 or 37 or 38 |  |
| 40. 31 and 35 and 39 |  |
| 41. limit 40 to yr="2010 -Current" |  |

Executed search Scopus

( ( TITLE-ABS-KEY ( cost*  OR  ( ( health  OR  health-care  OR  healthcare  OR  medical )  W/3  expenditure* )  OR  expense*  OR  "hospital resource*" ) )  OR  ( TITLE-ABS-KEY ( ( ( resource*  OR  "health service" )  W/3  ( "use"  OR  utilisation  OR  utilization ) )  OR  ( ( financial  OR  economic )  W/3  ( burden  OR  impact  OR  consequence ) )  OR  spending  OR  "out of pocket"  OR  out-of-pocket  OR  oop  OR  insurance  OR  absenteeism  OR  ( productivity  W/3  ( loss  OR  lost ) )  OR  "economic modelling" ) ) )  AND  ( ( TITLE-ABS-KEY ( ( condition*  OR  diagnos*  OR  disease*  OR  illness*  OR  "health problem*"  OR  patholog*  OR  disorder*  OR  syndrome* )  W/1  ( associated  OR  coexisting  OR  co-existing  OR  comorbid  OR  co-morbid  OR  concurrent  OR  co-occuring  OR  cooccuring  OR  multiple )  OR  "associated morbidit*" ) )  OR  ( TITLE-ABS-KEY ( comorbidit*  OR  co-morbidit*  OR  multidisease*  OR  multi-disease*  OR  multimorbidit*  OR  multi-morbidit*  OR  multipatholog*  OR  multi-patholog*  OR  pluripatholog*  OR  polypatholog*  OR  poly-pathology* ) ) )  AND  ( ( TITLE-ABS-KEY ( ( "chronic obstructive pulmonary disease"  OR  copd  OR  "pulmonary disease?"  OR  "pulmonary disorder?" )  AND  ( diabet*  OR  hypertens*  OR  "high blood pressure*"  OR  neoplasm?  OR  cancer?  OR  ( ( cardiac  OR  cardiovascular  OR  coronary )  W/1  ( disease?  OR  disorder?  OR  failure ) )  OR  "myocardial infarct*"  OR  "cardiovascular strok*"  OR  "heart failure"  OR  "cardiac failure"  OR  "myocardial failure"  OR  asthma*  OR  ( ( mental  OR  anxiety  OR  mood  OR  psychological )  W/1  ( disease?  OR  disorder? ) )  OR  "sleep disorder"  OR  depression?  OR  osteoporosis  OR  dyslipid?emia*  OR  hyperlipid?emia*  OR  hypercholesterolemia*  OR  hypertriglyceridemia*  OR  "thyroid disease?"  OR  "thyroid disorder?"  OR  hyperthyroid*  OR  hypothyroid*  OR  "joint disease*"  OR  "rheumatoid arthritis"  OR  osteoarthritis  OR  "kidney disease?"  OR  "kidney disorder?"  OR  epilep*  OR  seizure?  OR  hiv  OR  "acquired immun* deficiency syndrome?"  OR  aids  OR  "liver disease?"  OR  "liver disorder?"  OR  stroke*  OR  dementia  OR  "cardiac arrhythmia*"  OR  an?emia*  OR  obesity  OR  obese  OR  "prostatic hyperplasia"  OR  "prostatic hypertrophy"  OR  tuberculosis  OR  "chronic hepatitis"  OR  "substance-related disorder?"  OR  "substance abuse"  OR  "drug abuse"  OR  "marijuana abuse"  OR  "cocaine abuse"  OR  "amphetamine abuse"  OR  addiction?  OR  "inflammatory bowel disease?"  OR  "ulcerative colitis"  OR  crohn  OR  ibd  OR  deaf*  OR  blind*  OR  "visual loss"  OR  parkinson*  OR  autis* ) ) )  OR  ( TITLE-ABS-KEY ( ( diabet* )  AND  ( hypertens*  OR  "high blood pressure*"  OR  neoplasm?  OR  cancer?  OR  ( ( cardiac  OR  cardiovascular  OR  coronary )  W/1  ( disease?  OR  disorder?  OR  failure ) )  OR  "myocardial infarct*"  OR  "cardiovascular strok*"  OR  "heart failure"  OR  "cardiac failure"  OR  "myocardial failure"  OR  asthma*  OR  ( ( mental  OR  anxiety  OR  mood  OR  psychological )  W/1  ( disease?  OR  disorder? ) )  OR  "sleep disorder"  OR  depression?  OR  osteoporosis  OR  dyslipid?emia*  OR  hyperlipid?emia*  OR  hypercholesterolemia*  OR  hypertriglyceridemia*  OR  "thyroid disease?"  OR  "thyroid disorder?"  OR  hyperthyroid*  OR  hypothyroid*  OR  "joint disease*"  OR  "rheumatoid arthritis"  OR  osteoarthritis  OR  "kidney disease?"  OR  "kidney disorder?"  OR  epilep*  OR  seizure?  OR  hiv  OR  "acquired immun* deficiency syndrome?"  OR  aids  OR  "liver disease?"  OR  "liver disorder?"  OR  stroke*  OR  dementia  OR  "cardiac arrhythmia*"  OR  an?emia*  OR  obesity  OR  obese  OR  "prostatic hyperplasia"  OR  "prostatic hypertrophy"  OR  tuberculosis  OR  "chronic hepatitis"  OR  "substance-related disorder?"  OR  "substance abuse"  OR  "drug abuse"  OR  "marijuana abuse"  OR  "cocaine abuse"  OR  "amphetamine abuse"  OR  addiction?  OR  "inflammatory bowel disease?"  OR  "ulcerative colitis"  OR  crohn  OR  ibd  OR  deaf*  OR  blind*  OR  "visual loss"  OR  parkinson*  OR  autis* ) ) )  OR  ( TITLE-ABS-KEY ( ( hypertens*  OR  "high blood pressure*" )  AND  ( neoplasm?  OR  cancer?  OR  ( ( cardiac  OR  cardiovascular  OR  coronary )  W/1  ( disease?  OR  disorder?  OR  failure ) )  OR  "myocardial infarct*"  OR  "cardiovascular strok*"  OR  "heart failure"  OR  "cardiac failure"  OR  "myocardial failure"  OR  asthma*  OR  ( ( mental  OR  anxiety  OR  mood  OR  psychological )  W/1  ( disease?  OR  disorder? ) )  OR  "sleep disorder"  OR  depression?  OR  osteoporosis  OR  dyslipid?emia*  OR  hyperlipid?emia*  OR  hypercholesterolemia*  OR  hypertriglyceridemia*  OR  "thyroid disease?"  OR  "thyroid disorder?"  OR  hyperthyroid*  OR  hypothyroid*  OR  "joint disease*"  OR  "rheumatoid arthritis"  OR  osteoarthritis  OR  "kidney disease?"  OR  "kidney disorder?"  OR  epilep*  OR  seizure?  OR  hiv  OR  "acquired immun* deficiency syndrome?"  OR  aids  OR  "liver disease?"  OR  "liver disorder?"  OR  stroke*  OR  dementia  OR  "cardiac arrhythmia*"  OR  an?emia*  OR  obesity  OR  obese  OR  "prostatic hyperplasia"  OR  "prostatic hypertrophy"  OR  tuberculosis  OR  "chronic hepatitis"  OR  "substance-related disorder?"  OR  "substance abuse"  OR  "drug abuse"  OR  "marijuana abuse"  OR  "cocaine abuse"  OR  "amphetamine abuse"  OR  addiction?  OR  "inflammatory bowel disease?"  OR  "ulcerative colitis"  OR  crohn  OR  ibd  OR  deaf*  OR  blind*  OR  "visual loss"  OR  parkinson*  OR  autis* ) ) )  OR  ( TITLE-ABS-KEY ( ( neoplasm?  OR  cancer? )  AND  ( ( ( cardiac  OR  cardiovascular  OR  coronary )  W/1  ( disease?  OR  disorder?  OR  failure ) )  OR  "myocardial infarct*"  OR  "cardiovascular strok*"  OR  "heart failure"  OR  "cardiac failure"  OR  "myocardial failure"  OR  asthma*  OR  ( ( mental  OR  anxiety  OR  mood  OR  psychological )  W/1  ( disease?  OR  disorder? ) )  OR  "sleep disorder"  OR  depression?  OR  osteoporosis  OR  dyslipid?emia*  OR  hyperlipid?emia*  OR  hypercholesterolemia*  OR  hypertriglyceridemia*  OR  "thyroid disease?"  OR  "thyroid disorder?"  OR  hyperthyroid*  OR  hypothyroid*  OR  "joint disease*"  OR  "rheumatoid arthritis"  OR  osteoarthritis  OR  "kidney disease?"  OR  "kidney disorder?"  OR  epilep*  OR  seizure?  OR  hiv  OR  "acquired immun* deficiency syndrome?"  OR  aids  OR  "liver disease?"  OR  "liver disorder?"  OR  stroke*  OR  dementia  OR  "cardiac arrhythmia*"  OR  an?emia*  OR  obesity  OR  obese  OR  "prostatic hyperplasia"  OR  "prostatic hypertrophy"  OR  tuberculosis  OR  "chronic hepatitis"  OR  "substance-related disorder?"  OR  "substance abuse"  OR  "drug abuse"  OR  "marijuana abuse"  OR  "cocaine abuse"  OR  "amphetamine abuse"  OR  addiction?  OR  "inflammatory bowel disease?"  OR  "ulcerative colitis"  OR  crohn  OR  ibd  OR  deaf*  OR  blind*  OR  "visual loss"  OR  parkinson*  OR  autis* ) ) )  OR  ( TITLE-ABS-KEY ( ( ( cardiac  OR  cardiovascular  OR  coronary )  W/1  ( disease?  OR  disorder?  OR  failure ) )  AND  ( "myocardial infarct*"  OR  "cardiovascular strok*"  OR  "heart failure"  OR  "cardiac failure"  OR  "myocardial failure"  OR  asthma*  OR  ( ( mental  OR  anxiety  OR  mood  OR  psychological )  W/1  ( disease?  OR  disorder? ) )  OR  "sleep disorder"  OR  depression?  OR  osteoporosis  OR  dyslipid?emia*  OR  hyperlipid?emia*  OR  hypercholesterolemia*  OR  hypertriglyceridemia*  OR  "thyroid disease?"  OR  "thyroid disorder?"  OR  hyperthyroid*  OR  hypothyroid*  OR  "joint disease*"  OR  "rheumatoid arthritis"  OR  osteoarthritis  OR  "kidney disease?"  OR  "kidney disorder?"  OR  epilep*  OR  seizure?  OR  hiv  OR  "acquired immun* deficiency syndrome?"  OR  aids  OR  "liver disease?"  OR  "liver disorder?"  OR  stroke*  OR  dementia  OR  "cardiac arrhythmia*"  OR  an?emia*  OR  obesity  OR  obese  OR  "prostatic hyperplasia"  OR  "prostatic hypertrophy"  OR  tuberculosis  OR  "chronic hepatitis"  OR  "substance-related disorder?"  OR  "substance abuse"  OR  "drug abuse"  OR  "marijuana abuse"  OR  "cocaine abuse"  OR  "amphetamine abuse"  OR  addiction?  OR  "inflammatory bowel disease?"  OR  "ulcerative colitis"  OR  crohn  OR  ibd  OR  deaf*  OR  blind*  OR  "visual loss"  OR  parkinson*  OR  autis* ) ) )  OR  ( TITLE-ABS-KEY ( ( "myocardial infarct*"  OR  "cardiovascular strok*" )  AND  ( "heart failure"  OR  "cardiac failure"  OR  "myocardial failure"  OR  asthma*  OR  ( ( mental  OR  anxiety  OR  mood  OR  psychological )  W/1  ( disease?  OR  disorder? ) )  OR  "sleep disorder"  OR  depression?  OR  osteoporosis  OR  dyslipid?emia*  OR  hyperlipid?emia*  OR  hypercholesterolemia*  OR  hypertriglyceridemia*  OR  "thyroid disease?"  OR  "thyroid disorder?"  OR  hyperthyroid*  OR  hypothyroid*  OR  "joint disease*"  OR  "rheumatoid arthritis"  OR  osteoarthritis  OR  "kidney disease?"  OR  "kidney disorder?"  OR  epilep*  OR  seizure?  OR  hiv  OR  "acquired immun* deficiency syndrome?"  OR  aids  OR  "liver disease?"  OR  "liver disorder?"  OR  stroke*  OR  dementia  OR  "cardiac arrhythmia*"  OR  an?emia*  OR  obesity  OR  obese  OR  "prostatic hyperplasia"  OR  "prostatic hypertrophy"  OR  tuberculosis  OR  "chronic hepatitis"  OR  "substance-related disorder?"  OR  "substance abuse"  OR  "drug abuse"  OR  "marijuana abuse"  OR  "cocaine abuse"  OR  "amphetamine abuse"  OR  addiction?  OR  "inflammatory bowel disease?"  OR  "ulcerative colitis"  OR  crohn  OR  ibd  OR  deaf*  OR  blind*  OR  "visual loss"  OR  parkinson*  OR  autis* ) ) )  OR  ( TITLE-ABS-KEY ( ( "heart failure"  OR  "cardiac failure"  OR  "myocardial failure" )  AND  ( asthma*  OR  ( ( mental  OR  anxiety  OR  mood  OR  psychological )  W/1  ( disease?  OR  disorder? ) )  OR  "sleep disorder"  OR  depression?  OR  osteoporosis  OR  dyslipid?emia*  OR  hyperlipid?emia*  OR  hypercholesterolemia*  OR  hypertriglyceridemia*  OR  "thyroid disease?"  OR  "thyroid disorder?"  OR  hyperthyroid*  OR  hypothyroid*  OR  "joint disease*"  OR  "rheumatoid arthritis"  OR  osteoarthritis  OR  "kidney disease?"  OR  "kidney disorder?"  OR  epilep*  OR  seizure?  OR  hiv  OR  "acquired immun* deficiency syndrome?"  OR  aids  OR  "liver disease?"  OR  "liver disorder?"  OR  stroke*  OR  dementia  OR  "cardiac arrhythmia*"  OR  an?emia*  OR  obesity  OR  obese  OR  "prostatic hyperplasia"  OR  "prostatic hypertrophy"  OR  tuberculosis  OR  "chronic hepatitis"  OR  "substance-related disorder?"  OR  "substance abuse"  OR  "drug abuse"  OR  "marijuana abuse"  OR  "cocaine abuse"  OR  "amphetamine abuse"  OR  addiction?  OR  "inflammatory bowel disease?"  OR  "ulcerative colitis"  OR  crohn  OR  ibd  OR  deaf*  OR  blind*  OR  "visual loss"  OR  parkinson*  OR  autis* ) ) )  OR  ( TITLE-ABS-KEY ( ( asthma* )  AND  ( ( ( mental  OR  anxiety  OR  mood  OR  psychological )  W/1  ( disease?  OR  disorder? ) )  OR  "sleep disorder"  OR  depression?  OR  osteoporosis  OR  dyslipid?emia*  OR  hyperlipid?emia*  OR  hypercholesterolemia*  OR  hypertriglyceridemia*  OR  "thyroid disease?"  OR  "thyroid disorder?"  OR  hyperthyroid*  OR  hypothyroid*  OR  "joint disease*"  OR  "rheumatoid arthritis"  OR  osteoarthritis  OR  "kidney disease?"  OR  "kidney disorder?"  OR  epilep*  OR  seizure?  OR  hiv  OR  "acquired immun* deficiency syndrome?"  OR  aids  OR  "liver disease?"  OR  "liver disorder?"  OR  stroke*  OR  dementia  OR  "cardiac arrhythmia*"  OR  an?emia*  OR  obesity  OR  obese  OR  "prostatic hyperplasia"  OR  "prostatic hypertrophy"  OR  tuberculosis  OR  "chronic hepatitis"  OR  "substance-related disorder?"  OR  "substance abuse"  OR  "drug abuse"  OR  "marijuana abuse"  OR  "cocaine abuse"  OR  "amphetamine abuse"  OR  addiction?  OR  "inflammatory bowel disease?"  OR  "ulcerative colitis"  OR  crohn  OR  ibd  OR  deaf*  OR  blind*  OR  "visual loss"  OR  parkinson*  OR  autis* ) ) )  OR  ( TITLE-ABS-KEY ( ( ( ( mental  OR  anxiety  OR  mood  OR  psychological )  W/1  ( disease?  OR  disorder? ) )  OR  "sleep disorder"  OR  depression? )  AND  ( osteoporosis  OR  dyslipid?emia*  OR  hyperlipid?emia*  OR  hypercholesterolemia*  OR  hypertriglyceridemia*  OR  "thyroid disease?"  OR  "thyroid disorder?"  OR  hyperthyroid*  OR  hypothyroid*  OR  "joint disease*"  OR  "rheumatoid arthritis"  OR  osteoarthritis  OR  "kidney disease?"  OR  "kidney disorder?"  OR  epilep*  OR  seizure?  OR  hiv  OR  "acquired immun* deficiency syndrome?"  OR  aids  OR  "liver disease?"  OR  "liver disorder?"  OR  stroke*  OR  dementia  OR  "cardiac arrhythmia*"  OR  an?emia*  OR  obesity  OR  obese  OR  "prostatic hyperplasia"  OR  "prostatic hypertrophy"  OR  tuberculosis  OR  "chronic hepatitis"  OR  "substance-related disorder?"  OR  "substance abuse"  OR  "drug abuse"  OR  "marijuana abuse"  OR  "cocaine abuse"  OR  "amphetamine abuse"  OR  addiction?  OR  "inflammatory bowel disease?"  OR  "ulcerative colitis"  OR  crohn  OR  ibd  OR  deaf*  OR  blind*  OR  "visual loss"  OR  parkinson*  OR  autis* ) ) )  OR  ( TITLE-ABS-KEY ( ( osteoporosis )  AND  ( dyslipid?emia*  OR  hyperlipid?emia*  OR  hypercholesterolemia*  OR  hypertriglyceridemia*  OR  "thyroid disease?"  OR  "thyroid disorder?"  OR  hyperthyroid*  OR  hypothyroid*  OR  "joint disease*"  OR  "rheumatoid arthritis"  OR  osteoarthritis  OR  "kidney disease?"  OR  "kidney disorder?"  OR  epilep*  OR  seizure?  OR  hiv  OR  "acquired immun* deficiency syndrome?"  OR  aids  OR  "liver disease?"  OR  "liver disorder?"  OR  stroke*  OR  dementia  OR  "cardiac arrhythmia*"  OR  an?emia*  OR  obesity  OR  obese  OR  "prostatic hyperplasia"  OR  "prostatic hypertrophy"  OR  tuberculosis  OR  "chronic hepatitis"  OR  "substance-related disorder?"  OR  "substance abuse"  OR  "drug abuse"  OR  "marijuana abuse"  OR  "cocaine abuse"  OR  "amphetamine abuse"  OR  addiction?  OR  "inflammatory bowel disease?"  OR  "ulcerative colitis"  OR  crohn  OR  ibd  OR  deaf*  OR  blind*  OR  "visual loss"  OR  parkinson*  OR  autis* ) ) )  OR  ( TITLE-ABS-KEY ( ( dyslipid?emia*  OR  hyperlipid?emia*  OR  hypercholesterolemia*  OR  hypertriglyceridemia* )  AND  ( "thyroid disease?"  OR  "thyroid disorder?"  OR  hyperthyroid*  OR  hypothyroid*  OR  "joint disease*"  OR  "rheumatoid arthritis"  OR  osteoarthritis  OR  "kidney disease?"  OR  "kidney disorder?"  OR  epilep*  OR  seizure?  OR  hiv  OR  "acquired immun* deficiency syndrome?"  OR  aids  OR  "liver disease?"  OR  "liver disorder?"  OR  stroke*  OR  dementia  OR  "cardiac arrhythmia*"  OR  an?emia*  OR  obesity  OR  obese  OR  "prostatic hyperplasia"  OR  "prostatic hypertrophy"  OR  tuberculosis  OR  "chronic hepatitis"  OR  "substance-related disorder?"  OR  "substance abuse"  OR  "drug abuse"  OR  "marijuana abuse"  OR  "cocaine abuse"  OR  "amphetamine abuse"  OR  addiction?  OR  "inflammatory bowel disease?"  OR  "ulcerative colitis"  OR  crohn  OR  ibd  OR  deaf*  OR  blind*  OR  "visual loss"  OR  parkinson*  OR  autis* ) ) )  OR  ( TITLE-ABS-KEY ( ( "thyroid disease?"  OR  "thyroid disorder?"  OR  hyperthyroid*  OR  hypothyroid* )  AND  ( "joint disease*"  OR  "rheumatoid arthritis"  OR  osteoarthritis  OR  "kidney disease?"  OR  "kidney disorder?"  OR  epilep*  OR  seizure?  OR  hiv  OR  "acquired immun* deficiency syndrome?"  OR  aids  OR  "liver disease?"  OR  "liver disorder?"  OR  stroke*  OR  dementia  OR  "cardiac arrhythmia*"  OR  an?emia*  OR  obesity  OR  obese  OR  "prostatic hyperplasia"  OR  "prostatic hypertrophy"  OR  tuberculosis  OR  "chronic hepatitis"  OR  "substance-related disorder?"  OR  "substance abuse"  OR  "drug abuse"  OR  "marijuana abuse"  OR  "cocaine abuse"  OR  "amphetamine abuse"  OR  addiction?  OR  "inflammatory bowel disease?"  OR  "ulcerative colitis"  OR  crohn  OR  ibd  OR  deaf*  OR  blind*  OR  "visual loss"  OR  parkinson*  OR  autis* ) ) )  OR  ( TITLE-ABS-KEY ( ( "joint disease*"  OR  "rheumatoid arthritis"  OR  osteoarthritis )  AND  ( "kidney disease?"  OR  "kidney disorder?"  OR  epilep*  OR  seizure?  OR  hiv  OR  "acquired immun* deficiency syndrome?"  OR  aids  OR  "liver disease?"  OR  "liver disorder?"  OR  stroke*  OR  dementia  OR  "cardiac arrhythmia*"  OR  an?emia*  OR  obesity  OR  obese  OR  "prostatic hyperplasia"  OR  "prostatic hypertrophy"  OR  tuberculosis  OR  "chronic hepatitis"  OR  "substance-related disorder?"  OR  "substance abuse"  OR  "drug abuse"  OR  "marijuana abuse"  OR  "cocaine abuse"  OR  "amphetamine abuse"  OR  addiction?  OR  "inflammatory bowel disease?"  OR  "ulcerative colitis"  OR  crohn  OR  ibd  OR  deaf*  OR  blind*  OR  "visual loss"  OR  parkinson*  OR  autis* ) ) )  OR  ( TITLE-ABS-KEY ( ( "kidney disease?"  OR  "kidney disorder?" )  AND  ( epilep*  OR  seizure?  OR  hiv  OR  "acquired immun* deficiency syndrome?"  OR  aids  OR  "liver disease?"  OR  "liver disorder?"  OR  stroke*  OR  dementia  OR  "cardiac arrhythmia*"  OR  an?emia*  OR  obesity  OR  obese  OR  "prostatic hyperplasia"  OR  "prostatic hypertrophy"  OR  tuberculosis  OR  "chronic hepatitis"  OR  "substance-related disorder?"  OR  "substance abuse"  OR  "drug abuse"  OR  "marijuana abuse"  OR  "cocaine abuse"  OR  "amphetamine abuse"  OR  addiction?  OR  "inflammatory bowel disease?"  OR  "ulcerative colitis"  OR  crohn  OR  ibd  OR  deaf*  OR  blind*  OR  "visual loss"  OR  parkinson*  OR  autis* ) ) )  OR  ( TITLE-ABS-KEY ( ( epilep*  OR  seizure? )  AND  ( hiv  OR  "acquired immun* deficiency syndrome?"  OR  aids  OR  "liver disease?"  OR  "liver disorder?"  OR  stroke*  OR  dementia  OR  "cardiac arrhythmia*"  OR  an?emia*  OR  obesity  OR  obese  OR  "prostatic hyperplasia"  OR  "prostatic hypertrophy"  OR  tuberculosis  OR  "chronic hepatitis"  OR  "substance-related disorder?"  OR  "substance abuse"  OR  "drug abuse"  OR  "marijuana abuse"  OR  "cocaine abuse"  OR  "amphetamine abuse"  OR  addiction?  OR  "inflammatory bowel disease?"  OR  "ulcerative colitis"  OR  crohn  OR  ibd  OR  deaf*  OR  blind*  OR  "visual loss"  OR  parkinson*  OR  autis* ) ) )  OR  ( TITLE-ABS-KEY ( ( hiv  OR  "acquired immun* deficiency syndrome?"  OR  aids )  AND  ( "liver disease?"  OR  "liver disorder?"  OR  stroke*  OR  dementia  OR  "cardiac arrhythmia*"  OR  an?emia*  OR  obesity  OR  obese  OR  "prostatic hyperplasia"  OR  "prostatic hypertrophy"  OR  tuberculosis  OR  "chronic hepatitis"  OR  "substance-related disorder?"  OR  "substance abuse"  OR  "drug abuse"  OR  "marijuana abuse"  OR  "cocaine abuse"  OR  "amphetamine abuse"  OR  addiction?  OR  "inflammatory bowel disease?"  OR  "ulcerative colitis"  OR  crohn  OR  ibd  OR  deaf*  OR  blind*  OR  "visual loss"  OR  parkinson*  OR  autis* ) ) )  OR  ( TITLE-ABS-KEY ( ( "liver disease?"  OR  "liver disorder?" )  AND  ( stroke*  OR  dementia  OR  "cardiac arrhythmia*"  OR  an?emia*  OR  obesity  OR  obese  OR  "prostatic hyperplasia"  OR  "prostatic hypertrophy"  OR  tuberculosis  OR  "chronic hepatitis"  OR  "substance-related disorder?"  OR  "substance abuse"  OR  "drug abuse"  OR  "marijuana abuse"  OR  "cocaine abuse"  OR  "amphetamine abuse"  OR  addiction?  OR  "inflammatory bowel disease?"  OR  "ulcerative colitis"  OR  crohn  OR  ibd  OR  deaf*  OR  blind*  OR  "visual loss"  OR  parkinson*  OR  autis* ) ) )  OR  ( TITLE-ABS-KEY ( ( stroke* )  AND  ( dementia  OR  "cardiac arrhythmia*"  OR  an?emia*  OR  obesity  OR  obese  OR  "prostatic hyperplasia"  OR  "prostatic hypertrophy"  OR  tuberculosis  OR  "chronic hepatitis"  OR  "substance-related disorder?"  OR  "substance abuse"  OR  "drug abuse"  OR  "marijuana abuse"  OR  "cocaine abuse"  OR  "amphetamine abuse"  OR  addiction?  OR  "inflammatory bowel disease?"  OR  "ulcerative colitis"  OR  crohn  OR  ibd  OR  deaf*  OR  blind*  OR  "visual loss"  OR  parkinson*  OR  autis* ) ) )  OR  ( TITLE-ABS-KEY ( ( dementia )  AND  ( "cardiac arrhythmia*"  OR  an?emia*  OR  obesity  OR  obese  OR  "prostatic hyperplasia"  OR  "prostatic hypertrophy"  OR  tuberculosis  OR  "chronic hepatitis"  OR  "substance-related disorder?"  OR  "substance abuse"  OR  "drug abuse"  OR  "marijuana abuse"  OR  "cocaine abuse"  OR  "amphetamine abuse"  OR  addiction?  OR  "inflammatory bowel disease?"  OR  "ulcerative colitis"  OR  crohn  OR  ibd  OR  deaf*  OR  blind*  OR  "visual loss"  OR  parkinson*  OR  autis* ) ) )  OR  ( TITLE-ABS-KEY ( ( "cardiac arrhythmia*" )  AND  ( an?emia*  OR  obesity  OR  obese  OR  "prostatic hyperplasia"  OR  "prostatic hypertrophy"  OR  tuberculosis  OR  "chronic hepatitis"  OR  "substance-related disorder?"  OR  "substance abuse"  OR  "drug abuse"  OR  "marijuana abuse"  OR  "cocaine abuse"  OR  "amphetamine abuse"  OR  addiction?  OR  "inflammatory bowel disease?"  OR  "ulcerative colitis"  OR  crohn  OR  ibd  OR  deaf*  OR  blind*  OR  "visual loss"  OR  parkinson*  OR  autis* ) ) )  OR  ( TITLE-ABS-KEY ( ( an?emia* )  AND  ( obesity  OR  obese  OR  "prostatic hyperplasia"  OR  "prostatic hypertrophy"  OR  tuberculosis  OR  "chronic hepatitis"  OR  "substance-related disorder?"  OR  "substance abuse"  OR  "drug abuse"  OR  "marijuana abuse"  OR  "cocaine abuse"  OR  "amphetamine abuse"  OR  addiction?  OR  "inflammatory bowel disease?"  OR  "ulcerative colitis"  OR  crohn  OR  ibd  OR  deaf*  OR  blind*  OR  "visual loss"  OR  parkinson*  OR  autis* ) ) )  OR  ( TITLE-ABS-KEY ( ( obesity  OR  obese )  AND  ( "prostatic hyperplasia"  OR  "prostatic hypertrophy"  OR  tuberculosis  OR  "chronic hepatitis"  OR  "substance-related disorder?"  OR  "substance abuse"  OR  "drug abuse"  OR  "marijuana abuse"  OR  "cocaine abuse"  OR  "amphetamine abuse"  OR  addiction?  OR  "inflammatory bowel disease?"  OR  "ulcerative colitis"  OR  crohn  OR  ibd  OR  deaf*  OR  blind*  OR  "visual loss"  OR  parkinson*  OR  autis* ) ) )  OR  ( TITLE-ABS-KEY ( ( "prostatic hyperplasia"  OR  "prostatic hypertrophy" )  AND  ( tuberculosis  OR  "chronic hepatitis"  OR  "substance-related disorder?"  OR  "substance abuse"  OR  "drug abuse"  OR  "marijuana abuse"  OR  "cocaine abuse"  OR  "amphetamine abuse"  OR  addiction?  OR  "inflammatory bowel disease?"  OR  "ulcerative colitis"  OR  crohn  OR  ibd  OR  deaf*  OR  blind*  OR  "visual loss"  OR  parkinson*  OR  autis* ) ) )  OR  ( TITLE-ABS-KEY ( ( tuberculosis )  AND  ( "chronic hepatitis"  OR  "substance-related disorder?"  OR  "substance abuse"  OR  "drug abuse"  OR  "marijuana abuse"  OR  "cocaine abuse"  OR  "amphetamine abuse"  OR  addiction?  OR  "inflammatory bowel disease?"  OR  "ulcerative colitis"  OR  crohn  OR  ibd  OR  deaf*  OR  blind*  OR  "visual loss"  OR  parkinson*  OR  autis* ) ) )  OR  ( TITLE-ABS-KEY ( ( "chronic hepatitis" )  AND  ( "substance-related disorder?"  OR  "substance abuse"  OR  "drug abuse"  OR  "marijuana abuse"  OR  "cocaine abuse"  OR  "amphetamine abuse"  OR  addiction?  OR  "inflammatory bowel disease?"  OR  "ulcerative colitis"  OR  crohn  OR  ibd  OR  deaf*  OR  blind*  OR  "visual loss"  OR  parkinson*  OR  autis* ) ) )  OR  ( TITLE-ABS-KEY ( ( "substance-related disorder?"  OR  "substance abuse"  OR  "drug abuse"  OR  "marijuana abuse"  OR  "cocaine abuse"  OR  "amphetamine abuse"  OR  addiction? )  AND  ( "inflammatory bowel disease?"  OR  "ulcerative colitis"  OR  crohn  OR  ibd  OR  deaf*  OR  blind*  OR  "visual loss"  OR  parkinson*  OR  autis* ) ) )  OR  ( TITLE-ABS-KEY ( ( "inflammatory bowel disease?"  OR  "ulcerative colitis"  OR  crohn  OR  ibd )  AND  ( deaf*  OR  blind*  OR  "visual loss"  OR  parkinson*  OR  autis* ) ) )  OR  ( TITLE-ABS-KEY ( ( deaf* )  AND  ( blind*  OR  "visual loss"  OR  parkinson*  OR  autis* ) ) )  OR  ( TITLE-ABS-KEY ( ( blind*  OR  "visual loss" )  AND  ( parkinson*  OR  autis* ) ) )  OR  ( TITLE-ABS-KEY ( parkinson*  AND  autis* ) ) )  AND NOT  INDEX ( medline )  AND  ( LIMIT-TO ( PUBYEAR ,  2020 )  OR  LIMIT-TO ( PUBYEAR ,  2019 )  OR  LIMIT-TO ( PUBYEAR ,  2018 )  OR  LIMIT-TO ( PUBYEAR ,  2017 )  OR  LIMIT-TO ( PUBYEAR ,  2016 )  OR  LIMIT-TO ( PUBYEAR ,  2015 )  OR  LIMIT-TO ( PUBYEAR ,  2014 )  OR  LIMIT-TO ( PUBYEAR ,  2013 )  OR  LIMIT-TO ( PUBYEAR ,  2012 )  OR  LIMIT-TO ( PUBYEAR ,  2011 )  OR  LIMIT-TO ( PUBYEAR ,  2010 ) )

Executed search BSC

| **#** | **Query** | **Limiters/Expanders** | **Last Run Via** | **Results** |
| --- | --- | --- | --- | --- |
| S38 | S31 AND S34 AND S37 | Limiters - Articles on Several Companies  Expanders - Apply equivalent subjects  Search modes - Boolean/Phrase | Interface - EBSCOhost Research Databases  Search Screen - Advanced Search  Database - Business Source Complete | 1   - from 2003 |
| S37 | S35 OR S36 | Limiters - Articles on Several Companies  Expanders - Apply equivalent subjects  Search modes - Boolean/Phrase | Interface - EBSCOhost Research Databases  Search Screen - Advanced Search  Database - Business Source Complete | 147,121 |
| S36 | TI ( (((resource* or "health service") N3 ("use" or utilisation or utilization)) or ((financial or economic) N3 (burden or impact or consequence)) or spending or “out of pocket” or out-of-pocket or oop or insurance or absenteeism or (productivity N3 (loss or lost)) or "economic modelling") ) OR AB ( (((resource* or "health service") N3 ("use" or utilisation or utilization)) or ((financial or economic) N3 (burden or impact or consequence)) or spending or “out of pocket” or out-of-pocket or oop or insurance or absenteeism or (productivity N3 (loss or lost)) or "economic modelling") ) | Limiters - Articles on Several Companies  Expanders - Apply equivalent subjects  Search modes - Boolean/Phrase | Interface - EBSCOhost Research Databases  Search Screen - Advanced Search  Database - Business Source Complete | 71,984 |
| S35 | TI ( (cost* or ((health or health-care or healthcare or medical) N3 expenditure*) or expense* or “hospital resource*”) ) OR AB ( (cost* or ((health or health-care or healthcare or medical) N3 expenditure*) or expense* or “hospital resource*”) ) | Limiters - Articles on Several Companies  Expanders - Apply equivalent subjects  Search modes - Boolean/Phrase | Interface - EBSCOhost Research Databases  Search Screen - Advanced Search  Database - Business Source Complete | 79,464 |
| S34 | (S32 OR S33) | Limiters - Articles on Several Companies  Expanders - Apply equivalent subjects  Search modes - Boolean/Phrase | Interface - EBSCOhost Research Databases  Search Screen - Advanced Search  Database - Business Source Complete | 132 |
| S33 | TI ( (comorbidit* or co-morbidit* or multidisease* or multi-disease* or multimorbidit* or multi-morbidit* or multipatholog* or multi-patholog* or pluripatholog* or polypatholog* or poly-pathology*) ) OR AB ( (comorbidit* or co-morbidit* or multidisease* or multi-disease* or multimorbidit* or multi-morbidit* or multipatholog* or multi-patholog* or pluripatholog* or polypatholog* or poly-pathology*) ) | Limiters - Articles on Several Companies  Expanders - Apply equivalent subjects  Search modes - Boolean/Phrase | Interface - EBSCOhost Research Databases  Search Screen - Advanced Search  Database - Business Source Complete | 15 |
| S32 | TI ( ((condition* or diagnos* or disease* or illness* or “health problem*” or patholog* or disorder* or syndrome*) N1 (associated or coexisting or co-existing or comorbid or co-morbid or concurrent or co-occuring or cooccuring or multiple) or “associated morbidit*”) ) OR AB ( ((condition* or diagnos* or disease* or illness* or “health problem*” or patholog* or disorder* or syndrome*) N1 (associated or coexisting or co-existing or comorbid or co-morbid or concurrent or co-occuring or cooccuring or multiple) or “associated morbidit*”) ) | Limiters - Articles on Several Companies  Expanders - Apply equivalent subjects  Search modes - Boolean/Phrase | Interface - EBSCOhost Research Databases  Search Screen - Advanced Search  Database - Business Source Complete | 117 |
| S31 | (S1 OR S2 OR S3 OR S4 OR S5 OR S6 OR S7 OR S8 OR S9 OR S10 OR S11 OR S12 OR S13 OR S14 OR S15 OR S16 OR S17 OR S18 OR S19 OR S20 OR S21 OR S22 OR S23 OR S24 OR S25 OR S26 OR S27 OR S28 OR S29 OR S30) | Limiters - Articles on Several Companies  Expanders - Apply equivalent subjects  Search modes - Boolean/Phrase | Interface - EBSCOhost Research Databases  Search Screen - Advanced Search  Database - Business Source Complete | 1,138 |
| S30 | TI ( (parkinson* and autis*) ) OR AB ( (parkinson* and autis*) ) | Limiters - Articles on Several Companies  Expanders - Apply equivalent subjects  Search modes - Boolean/Phrase | Interface - EBSCOhost Research Databases  Search Screen - Advanced Search  Database - Business Source Complete | 2 |
| S29 | TI ( ((blind* or “visual loss”) and (parkinson* or autis*)) ) OR AB ( ((blind* or “visual loss”) and (parkinson* or autis*)) ) | Limiters - Articles on Several Companies  Expanders - Apply equivalent subjects  Search modes - Boolean/Phrase | Interface - EBSCOhost Research Databases  Search Screen - Advanced Search  Database - Business Source Complete | 4 |
| S28 | TI ( ((deaf*) and (blind* or “visual loss” or parkinson* or autis*)) ) OR AB ( ((deaf*) and (blind* or “visual loss” or parkinson* or autis*)) ) | Limiters - Articles on Several Companies  Expanders - Apply equivalent subjects  Search modes - Boolean/Phrase | Interface - EBSCOhost Research Databases  Search Screen - Advanced Search  Database - Business Source Complete | 17 |
| S27 | TI ( ((“inflammatory bowel disease?” or “ulcerative colitis” or crohn or ibd) and (deaf* or blind* or “visual loss” or parkinson* or autis*)) ) OR AB ( ((“inflammatory bowel disease?” or “ulcerative colitis” or crohn or ibd) and (deaf* or blind* or “visual loss” or parkinson* or autis*)) ) | Limiters - Articles on Several Companies  Expanders - Apply equivalent subjects  Search modes - Boolean/Phrase | Interface - EBSCOhost Research Databases  Search Screen - Advanced Search  Database - Business Source Complete | 2 |
| S26 | TI ( ((“substance-related disorder?” or “substance abuse” or “drug abuse” or “marijuana abuse” or “cocaine abuse” or “amphetamine abuse” or addiction?) and (“inflammatory bowel disease?” or “ulcerative colitis” or crohn or ibd or deaf* or blind* or “visual loss” or parkinson* or autis*)) ) OR AB ( ((“substance-related disorder?” or “substance abuse” or “drug abuse” or “marijuana abuse” or “cocaine abuse” or “amphetamine abuse” or addiction?) and (“inflammatory bowel disease?” or “ulcerative colitis” or crohn or ibd or deaf* or blind* or “visual loss” or parkinson* or autis*)) ) | Limiters - Articles on Several Companies  Expanders - Apply equivalent subjects  Search modes - Boolean/Phrase | Interface - EBSCOhost Research Databases  Search Screen - Advanced Search  Database - Business Source Complete | 1 |
| S25 | TI ( ((“chronic hepatitis”) and (“substance-related disorder?” or “substance abuse” or “drug abuse” or “marijuana abuse” or “cocaine abuse” or “amphetamine abuse” or addiction? or “inflammatory bowel disease?” or “ulcerative colitis” or crohn or ibd or deaf* or blind* or “visual loss” or parkinson* or autis*)) ) OR AB ( ((“chronic hepatitis”) and (“substance-related disorder?” or “substance abuse” or “drug abuse” or “marijuana abuse” or “cocaine abuse” or “amphetamine abuse” or addiction? or “inflammatory bowel disease?” or “ulcerative colitis” or crohn or ibd or deaf* or blind* or “visual loss” or parkinson* or autis*)) ) | Limiters - Articles on Several Companies  Expanders - Apply equivalent subjects  Search modes - Boolean/Phrase | Interface - EBSCOhost Research Databases  Search Screen - Advanced Search  Database - Business Source Complete | 1 |
| S24 | TI ( ((tuberculosis) and (“chronic hepatitis” or “substance-related disorder?” or “substance abuse” or “drug abuse” or “marijuana abuse” or “cocaine abuse” or “amphetamine abuse” or addiction? or “inflammatory bowel disease?” or “ulcerative colitis” or crohn or ibd or deaf* or blind* or “visual loss” or parkinson* or autis*)) ) OR AB ( ((tuberculosis) and (“chronic hepatitis” or “substance-related disorder?” or “substance abuse” or “drug abuse” or “marijuana abuse” or “cocaine abuse” or “amphetamine abuse” or addiction? or “inflammatory bowel disease?” or “ulcerative colitis” or crohn or ibd or deaf* or blind* or “visual loss” or parkinson* or autis*)) ) | Limiters - Articles on Several Companies  Expanders - Apply equivalent subjects  Search modes - Boolean/Phrase | Interface - EBSCOhost Research Databases  Search Screen - Advanced Search  Database - Business Source Complete | 4 |
| S23 | TI ( ((“prostatic hyperplasia” or “prostatic hypertrophy”) and (tuberculosis or “chronic hepatitis” or “substance-related disorder?” or “substance abuse” or “drug abuse” or “marijuana abuse” or “cocaine abuse” or “amphetamine abuse” or addiction? or “inflammatory bowel disease?” or “ulcerative colitis” or crohn or ibd or deaf* or blind* or “visual loss” or parkinson* or autis*)) ) OR AB ( ((“prostatic hyperplasia” or “prostatic hypertrophy”) and (tuberculosis or “chronic hepatitis” or “substance-related disorder?” or “substance abuse” or “drug abuse” or “marijuana abuse” or “cocaine abuse” or “amphetamine abuse” or addiction? or “inflammatory bowel disease?” or “ulcerative colitis” or crohn or ibd or deaf* or blind* or “visual loss” or parkinson* or autis*)) ) | Limiters - Articles on Several Companies  Expanders - Apply equivalent subjects  Search modes - Boolean/Phrase | Interface - EBSCOhost Research Databases  Search Screen - Advanced Search  Database - Business Source Complete | 0 |
| S22 | TI ( ((obesity or obese) and (“prostatic hyperplasia” or “prostatic hypertrophy” or tuberculosis or “chronic hepatitis” or “substance-related disorder?” or “substance abuse” or “drug abuse” or “marijuana abuse” or “cocaine abuse” or “amphetamine abuse” or addiction? or “inflammatory bowel disease?” or “ulcerative colitis” or crohn or ibd or deaf* or blind* or “visual loss” or parkinson* or autis*)) ) OR AB ( ((obesity or obese) and (“prostatic hyperplasia” or “prostatic hypertrophy” or tuberculosis or “chronic hepatitis” or “substance-related disorder?” or “substance abuse” or “drug abuse” or “marijuana abuse” or “cocaine abuse” or “amphetamine abuse” or addiction? or “inflammatory bowel disease?” or “ulcerative colitis” or crohn or ibd or deaf* or blind* or “visual loss” or parkinson* or autis*)) ) | Limiters - Articles on Several Companies  Expanders - Apply equivalent subjects  Search modes - Boolean/Phrase | Interface - EBSCOhost Research Databases  Search Screen - Advanced Search  Database - Business Source Complete | 3 |
| S21 | TI ( ((an?emia*) and (obesity or obese or “prostatic hyperplasia” or “prostatic hypertrophy” or tuberculosis or “chronic hepatitis” or “substance-related disorder?” or “substance abuse” or “drug abuse” or “marijuana abuse” or “cocaine abuse” or “amphetamine abuse” or addiction? or “inflammatory bowel disease?” or “ulcerative colitis” or crohn or ibd or deaf* or blind* or “visual loss” or parkinson* or autis*)) ) OR AB ( ((an?emia*) and (obesity or obese or “prostatic hyperplasia” or “prostatic hypertrophy” or tuberculosis or “chronic hepatitis” or “substance-related disorder?” or “substance abuse” or “drug abuse” or “marijuana abuse” or “cocaine abuse” or “amphetamine abuse” or addiction? or “inflammatory bowel disease?” or “ulcerative colitis” or crohn or ibd or deaf* or blind* or “visual loss” or parkinson* or autis*)) ) | Limiters - Articles on Several Companies  Expanders - Apply equivalent subjects  Search modes - Boolean/Phrase | Interface - EBSCOhost Research Databases  Search Screen - Advanced Search  Database - Business Source Complete | 1 |
| S20 | TI ( ((“cardiac arrhythmia*”) and (an?emia* or obesity or obese or “prostatic hyperplasia” or “prostatic hypertrophy” or tuberculosis or “chronic hepatitis” or “substance-related disorder?” or “substance abuse” or “drug abuse” or “marijuana abuse” or “cocaine abuse” or “amphetamine abuse” or addiction? or “inflammatory bowel disease?” or “ulcerative colitis” or crohn or ibd or deaf* or blind* or “visual loss” or parkinson* or autis*)) ) OR AB ( ((“cardiac arrhythmia*”) and (an?emia* or obesity or obese or “prostatic hyperplasia” or “prostatic hypertrophy” or tuberculosis or “chronic hepatitis” or “substance-related disorder?” or “substance abuse” or “drug abuse” or “marijuana abuse” or “cocaine abuse” or “amphetamine abuse” or addiction? or “inflammatory bowel disease?” or “ulcerative colitis” or crohn or ibd or deaf* or blind* or “visual loss” or parkinson* or autis*)) ) | Limiters - Articles on Several Companies  Expanders - Apply equivalent subjects  Search modes - Boolean/Phrase | Interface - EBSCOhost Research Databases  Search Screen - Advanced Search  Database - Business Source Complete | 1 |
| S19 | TI ( ((dementia) and (“cardiac arrhythmia*” or an?emia* or obesity or obese or “prostatic hyperplasia” or “prostatic hypertrophy” or tuberculosis or “chronic hepatitis” or “substance-related disorder?” or “substance abuse” or “drug abuse” or “marijuana abuse” or “cocaine abuse” or “amphetamine abuse” or addiction? or “inflammatory bowel disease?” or “ulcerative colitis” or crohn or ibd or deaf* or blind* or “visual loss” or parkinson* or autis*)) ) OR AB ( ((dementia) and (“cardiac arrhythmia*” or an?emia* or obesity or obese or “prostatic hyperplasia” or “prostatic hypertrophy” or tuberculosis or “chronic hepatitis” or “substance-related disorder?” or “substance abuse” or “drug abuse” or “marijuana abuse” or “cocaine abuse” or “amphetamine abuse” or addiction? or “inflammatory bowel disease?” or “ulcerative colitis” or crohn or ibd or deaf* or blind* or “visual loss” or parkinson* or autis*)) ) | Limiters - Articles on Several Companies  Expanders - Apply equivalent subjects  Search modes - Boolean/Phrase | Interface - EBSCOhost Research Databases  Search Screen - Advanced Search  Database - Business Source Complete | 4 |
| S18 | TI ( ((stroke*) and (dementia or “cardiac arrhythmia*” or an?emia* or obesity or obese or “prostatic hyperplasia” or “prostatic hypertrophy” or tuberculosis or “chronic hepatitis” or “substance-related disorder?” or “substance abuse” or “drug abuse” or “marijuana abuse” or “cocaine abuse” or “amphetamine abuse” or addiction? or “inflammatory bowel disease?” or “ulcerative colitis” or crohn or ibd or deaf* or blind* or “visual loss” or parkinson* or autis*)) ) OR AB ( ((stroke*) and (dementia or “cardiac arrhythmia*” or an?emia* or obesity or obese or “prostatic hyperplasia” or “prostatic hypertrophy” or tuberculosis or “chronic hepatitis” or “substance-related disorder?” or “substance abuse” or “drug abuse” or “marijuana abuse” or “cocaine abuse” or “amphetamine abuse” or addiction? or “inflammatory bowel disease?” or “ulcerative colitis” or crohn or ibd or deaf* or blind* or “visual loss” or parkinson* or autis*)) ) | Limiters - Articles on Several Companies  Expanders - Apply equivalent subjects  Search modes - Boolean/Phrase | Interface - EBSCOhost Research Databases  Search Screen - Advanced Search  Database - Business Source Complete | 18 |
| S17 | TI ( ((“liver disease?” or “liver disorder?”) and (stroke* or dementia or “cardiac arrhythmia*” or an?emia* or obesity or obese or “prostatic hyperplasia” or “prostatic hypertrophy” or tuberculosis or “chronic hepatitis” or “substance-related disorder?” or “substance abuse” or “drug abuse” or “marijuana abuse” or “cocaine abuse” or “amphetamine abuse” or addiction? or “inflammatory bowel disease?” or “ulcerative colitis” or crohn or ibd or deaf* or blind* or “visual loss” or parkinson* or autis*)) ) OR AB ( ((“liver disease?” or “liver disorder?”) and (stroke* or dementia or “cardiac arrhythmia*” or an?emia* or obesity or obese or “prostatic hyperplasia” or “prostatic hypertrophy” or tuberculosis or “chronic hepatitis” or “substance-related disorder?” or “substance abuse” or “drug abuse” or “marijuana abuse” or “cocaine abuse” or “amphetamine abuse” or addiction? or “inflammatory bowel disease?” or “ulcerative colitis” or crohn or ibd or deaf* or blind* or “visual loss” or parkinson* or autis*)) ) | Limiters - Articles on Several Companies  Expanders - Apply equivalent subjects  Search modes - Boolean/Phrase | Interface - EBSCOhost Research Databases  Search Screen - Advanced Search  Database - Business Source Complete | 2 |
| S16 | TI ( ((hiv or “acquired immun* deficiency syndrome?” or aids) and (“liver disease?” or “liver disorder?” or stroke* or dementia or “cardiac arrhythmia*” or an?emia* or obesity or obese or “prostatic hyperplasia” or “prostatic hypertrophy” or tuberculosis or “chronic hepatitis” or “substance-related disorder?” or “substance abuse” or “drug abuse” or “marijuana abuse” or “cocaine abuse” or “amphetamine abuse” or addiction? or “inflammatory bowel disease?” or “ulcerative colitis” or crohn or ibd or deaf* or blind* or “visual loss” or parkinson* or autis*)) ) OR AB ( ((hiv or “acquired immun* deficiency syndrome?” or aids) and (“liver disease?” or “liver disorder?” or stroke* or dementia or “cardiac arrhythmia*” or an?emia* or obesity or obese or “prostatic hyperplasia” or “prostatic hypertrophy” or tuberculosis or “chronic hepatitis” or “substance-related disorder?” or “substance abuse” or “drug abuse” or “marijuana abuse” or “cocaine abuse” or “amphetamine abuse” or addiction? or “inflammatory bowel disease?” or “ulcerative colitis” or crohn or ibd or deaf* or blind* or “visual loss” or parkinson* or autis*)) ) | Limiters - Articles on Several Companies  Expanders - Apply equivalent subjects  Search modes - Boolean/Phrase | Interface - EBSCOhost Research Databases  Search Screen - Advanced Search  Database - Business Source Complete | 113 |
| S15 | TI ( ((epilep* or seizure?) and (hiv or “acquired immun* deficiency syndrome?” or aids or “liver disease?” or “liver disorder?” or stroke* or dementia or “cardiac arrhythmia*” or an?emia* or obesity or obese or “prostatic hyperplasia” or “prostatic hypertrophy” or tuberculosis or “chronic hepatitis” or “substance-related disorder?” or “substance abuse” or “drug abuse” or “marijuana abuse” or “cocaine abuse” or “amphetamine abuse” or addiction? or “inflammatory bowel disease?” or “ulcerative colitis” or crohn or ibd or deaf* or blind* or “visual loss” or parkinson* or autis*)) ) OR AB ( ((epilep* or seizure?) and (hiv or “acquired immun* deficiency syndrome?” or aids or “liver disease?” or “liver disorder?” or stroke* or dementia or “cardiac arrhythmia*” or an?emia* or obesity or obese or “prostatic hyperplasia” or “prostatic hypertrophy” or tuberculosis or “chronic hepatitis” or “substance-related disorder?” or “substance abuse” or “drug abuse” or “marijuana abuse” or “cocaine abuse” or “amphetamine abuse” or addiction? or “inflammatory bowel disease?” or “ulcerative colitis” or crohn or ibd or deaf* or blind* or “visual loss” or parkinson* or autis*)) ) | Limiters - Articles on Several Companies  Expanders - Apply equivalent subjects  Search modes - Boolean/Phrase | Interface - EBSCOhost Research Databases  Search Screen - Advanced Search  Database - Business Source Complete | 15 |
| S14 | TI ( ((“kidney disease?” or “kidney disorder?”) and (epilep* or seizure? or hiv or “acquired immun* deficiency syndrome?” or aids or “liver disease?” or “liver disorder?” or stroke* or dementia or “cardiac arrhythmia*” or an?emia* or obesity or obese or “prostatic hyperplasia” or “prostatic hypertrophy” or tuberculosis or “chronic hepatitis” or “substance-related disorder?” or “substance abuse” or “drug abuse” or “marijuana abuse” or “cocaine abuse” or “amphetamine abuse” or addiction? or “inflammatory bowel disease?” or “ulcerative colitis” or crohn or ibd or deaf* or blind* or “visual loss” or parkinson* or autis*)) ) OR AB ( ((“kidney disease?” or “kidney disorder?”) and (epilep* or seizure? or hiv or “acquired immun* deficiency syndrome?” or aids or “liver disease?” or “liver disorder?” or stroke* or dementia or “cardiac arrhythmia*” or an?emia* or obesity or obese or “prostatic hyperplasia” or “prostatic hypertrophy” or tuberculosis or “chronic hepatitis” or “substance-related disorder?” or “substance abuse” or “drug abuse” or “marijuana abuse” or “cocaine abuse” or “amphetamine abuse” or addiction? or “inflammatory bowel disease?” or “ulcerative colitis” or crohn or ibd or deaf* or blind* or “visual loss” or parkinson* or autis*)) ) | Limiters - Articles on Several Companies  Expanders - Apply equivalent subjects  Search modes - Boolean/Phrase | Interface - EBSCOhost Research Databases  Search Screen - Advanced Search  Database - Business Source Complete | 10 |
| S13 | TI ( ((“joint disease*” or “rheumatoid arthritis” or osteoarthritis) and (“kidney disease?” or “kidney disorder?” or epilep* or seizure? or hiv or “acquired immun* deficiency syndrome?” or aids or “liver disease?” or “liver disorder?” or stroke* or dementia or “cardiac arrhythmia*” or an?emia* or obesity or obese or “prostatic hyperplasia” or “prostatic hypertrophy” or tuberculosis or “chronic hepatitis” or “substance-related disorder?” or “substance abuse” or “drug abuse” or “marijuana abuse” or “cocaine abuse” or “amphetamine abuse” or addiction? or “inflammatory bowel disease?” or “ulcerative colitis” or crohn or ibd or deaf* or blind* or “visual loss” or parkinson* or autis*)) ) OR AB ( ((“joint disease*” or “rheumatoid arthritis” or osteoarthritis) and (“kidney disease?” or “kidney disorder?” or epilep* or seizure? or hiv or “acquired immun* deficiency syndrome?” or aids or “liver disease?” or “liver disorder?” or stroke* or dementia or “cardiac arrhythmia*” or an?emia* or obesity or obese or “prostatic hyperplasia” or “prostatic hypertrophy” or tuberculosis or “chronic hepatitis” or “substance-related disorder?” or “substance abuse” or “drug abuse” or “marijuana abuse” or “cocaine abuse” or “amphetamine abuse” or addiction? or “inflammatory bowel disease?” or “ulcerative colitis” or crohn or ibd or deaf* or blind* or “visual loss” or parkinson* or autis*)) ) | Limiters - Articles on Several Companies  Expanders - Apply equivalent subjects  Search modes - Boolean/Phrase | Interface - EBSCOhost Research Databases  Search Screen - Advanced Search  Database - Business Source Complete | 34 |
| S12 | TI ( ((“thyroid disease?” or “thyroid disorder?” or hyperthyroid* or hypothyroid*) and (“joint disease*” or “rheumatoid arthritis” or osteoarthritis or “kidney disease?” or “kidney disorder?” or epilep* or seizure? or hiv or “acquired immun* deficiency syndrome?” or aids or “liver disease?” or “liver disorder?” or stroke* or dementia or “cardiac arrhythmia*” or an?emia* or obesity or obese or “prostatic hyperplasia” or “prostatic hypertrophy” or tuberculosis or “chronic hepatitis” or “substance-related disorder?” or “substance abuse” or “drug abuse” or “marijuana abuse” or “cocaine abuse” or “amphetamine abuse” or addiction? or “inflammatory bowel disease?” or “ulcerative colitis” or crohn or ibd or deaf* or blind* or “visual loss” or parkinson* or autis*)) ) OR AB ( ((“thyroid disease?” or “thyroid disorder?” or hyperthyroid* or hypothyroid*) and (“joint disease*” or “rheumatoid arthritis” or osteoarthritis or “kidney disease?” or “kidney disorder?” or epilep* or seizure? or hiv or “acquired immun* deficiency syndrome?” or aids or “liver disease?” or “liver disorder?” or stroke* or dementia or “cardiac arrhythmia*” or an?emia* or obesity or obese or “prostatic hyperplasia” or “prostatic hypertrophy” or tuberculosis or “chronic hepatitis” or “substance-related disorder?” or “substance abuse” or “drug abuse” or “marijuana abuse” or “cocaine abuse” or “amphetamine abuse” or addiction? or “inflammatory bowel disease?” or “ulcerative colitis” or crohn or ibd or deaf* or blind* or “visual loss” or parkinson* or autis*)) ) | Limiters - Articles on Several Companies  Expanders - Apply equivalent subjects  Search modes - Boolean/Phrase | Interface - EBSCOhost Research Databases  Search Screen - Advanced Search  Database - Business Source Complete | 1 |
| S11 | TI ( ((dyslipid?emia* or hyperlipid?emia* or hypercholesterolemia* or hypertriglyceridemia*) and (“thyroid disease?” or “thyroid disorder?” or hyperthyroid* or hypothyroid* or “joint disease*” or “rheumatoid arthritis” or osteoarthritis or “kidney disease?” or “kidney disorder?” or epilep* or seizure? or hiv or “acquired immun* deficiency syndrome?” or aids or “liver disease?” or “liver disorder?” or stroke* or dementia or “cardiac arrhythmia*” or an?emia* or obesity or obese or “prostatic hyperplasia” or “prostatic hypertrophy” or tuberculosis or “chronic hepatitis” or “substance-related disorder?” or “substance abuse” or “drug abuse” or “marijuana abuse” or “cocaine abuse” or “amphetamine abuse” or addiction? or “inflammatory bowel disease?” or “ulcerative colitis” or crohn or ibd or deaf* or blind* or “visual loss” or parkinson* or autis*)) ) OR AB ( ((dyslipid?emia* or hyperlipid?emia* or hypercholesterolemia* or hypertriglyceridemia*) and (“thyroid disease?” or “thyroid disorder?” or hyperthyroid* or hypothyroid* or “joint disease*” or “rheumatoid arthritis” or osteoarthritis or “kidney disease?” or “kidney disorder?” or epilep* or seizure? or hiv or “acquired immun* deficiency syndrome?” or aids or “liver disease?” or “liver disorder?” or stroke* or dementia or “cardiac arrhythmia*” or an?emia* or obesity or obese or “prostatic hyperplasia” or “prostatic hypertrophy” or tuberculosis or “chronic hepatitis” or “substance-related disorder?” or “substance abuse” or “drug abuse” or “marijuana abuse” or “cocaine abuse” or “amphetamine abuse” or addiction? or “inflammatory bowel disease?” or “ulcerative colitis” or crohn or ibd or deaf* or blind* or “visual loss” or parkinson* or autis*)) ) | Limiters - Articles on Several Companies  Expanders - Apply equivalent subjects  Search modes - Boolean/Phrase | Interface - EBSCOhost Research Databases  Search Screen - Advanced Search  Database - Business Source Complete | 5 |
| S10 | TI ( ((osteoporosis) and (dyslipid?emia* or hyperlipid?emia* or hypercholesterolemia* or hypertriglyceridemia* or “thyroid disease?” or “thyroid disorder?” or hyperthyroid* or hypothyroid* or “joint disease*” or “rheumatoid arthritis” or osteoarthritis or “kidney disease?” or “kidney disorder?” or epilep* or seizure? or hiv or “acquired immun* deficiency syndrome?” or aids or “liver disease?” or “liver disorder?” or stroke* or dementia or “cardiac arrhythmia*” or an?emia* or obesity or obese or “prostatic hyperplasia” or “prostatic hypertrophy” or tuberculosis or “chronic hepatitis” or “substance-related disorder?” or “substance abuse” or “drug abuse” or “marijuana abuse” or “cocaine abuse” or “amphetamine abuse” or addiction? or “inflammatory bowel disease?” or “ulcerative colitis” or crohn or ibd or deaf* or blind* or “visual loss” or parkinson* or autis*)) ) OR AB ( ((osteoporosis) and (dyslipid?emia* or hyperlipid?emia* or hypercholesterolemia* or hypertriglyceridemia* or “thyroid disease?” or “thyroid disorder?” or hyperthyroid* or hypothyroid* or “joint disease*” or “rheumatoid arthritis” or osteoarthritis or “kidney disease?” or “kidney disorder?” or epilep* or seizure? or hiv or “acquired immun* deficiency syndrome?” or aids or “liver disease?” or “liver disorder?” or stroke* or dementia or “cardiac arrhythmia*” or an?emia* or obesity or obese or “prostatic hyperplasia” or “prostatic hypertrophy” or tuberculosis or “chronic hepatitis” or “substance-related disorder?” or “substance abuse” or “drug abuse” or “marijuana abuse” or “cocaine abuse” or “amphetamine abuse” or addiction? or “inflammatory bowel disease?” or “ulcerative colitis” or crohn or ibd or deaf* or blind* or “visual loss” or parkinson* or autis*)) ) | Limiters - Articles on Several Companies  Expanders - Apply equivalent subjects  Search modes - Boolean/Phrase | Interface - EBSCOhost Research Databases  Search Screen - Advanced Search  Database - Business Source Complete | 20 |
| S9 | TI ( ((((mental or anxiety or mood or psychological) N1 (disease? or disorder?)) or “sleep disorder” or depression?) and (osteoporosis or dyslipid?emia* or hyperlipid?emia* or hypercholesterolemia* or hypertriglyceridemia* or “thyroid disease?” or “thyroid disorder?” or hyperthyroid* or hypothyroid* or “joint disease*” or “rheumatoid arthritis” or osteoarthritis or “kidney disease?” or “kidney disorder?” or epilep* or seizure? or hiv or “acquired immun* deficiency syndrome?” or aids or “liver disease?” or “liver disorder?” or stroke* or dementia or “cardiac arrhythmia*” or an?emia* or obesity or obese or “prostatic hyperplasia” or “prostatic hypertrophy” or tuberculosis or “chronic hepatitis” or “substance-related disorder?” or “substance abuse” or “drug abuse” or “marijuana abuse” or “cocaine abuse” or “amphetamine abuse” or addiction? or “inflammatory bowel disease?” or “ulcerative colitis” or crohn or ibd or deaf* or blind* or “visual loss” or parkinson* or autis*)) ) OR AB ( ((((mental or anxiety or mood or psychological) N1 (disease? or disorder?)) or “sleep disorder” or depression?) and (osteoporosis or dyslipid?emia* or hyperlipid?emia* or hypercholesterolemia* or hypertriglyceridemia* or “thyroid disease?” or “thyroid disorder?” or hyperthyroid* or hypothyroid* or “joint disease*” or “rheumatoid arthritis” or osteoarthritis or “kidney disease?” or “kidney disorder?” or epilep* or seizure? or hiv or “acquired immun* deficiency syndrome?” or aids or “liver disease?” or “liver disorder?” or stroke* or dementia or “cardiac arrhythmia*” or an?emia* or obesity or obese or “prostatic hyperplasia” or “prostatic hypertrophy” or tuberculosis or “chronic hepatitis” or “substance-related disorder?” or “substance abuse” or “drug abuse” or “marijuana abuse” or “cocaine abuse” or “amphetamine abuse” or addiction? or “inflammatory bowel disease?” or “ulcerative colitis” or crohn or ibd or deaf* or blind* or “visual loss” or parkinson* or autis*)) ) | Limiters - Articles on Several Companies  Expanders - Apply equivalent subjects  Search modes - Boolean/Phrase | Interface - EBSCOhost Research Databases  Search Screen - Advanced Search  Database - Business Source Complete | 49 |
| S8 | TI ( ((asthma*) AND (((mental or anxiety or mood or psychological) N1 (disease? or disorder?)) or “sleep disorder” or depression? or osteoporosis or dyslipid?emia* or hyperlipid?emia* or hypercholesterolemia* or hypertriglyceridemia* or “thyroid disease?” or “thyroid disorder?” or hyperthyroid* or hypothyroid* or “joint disease*” or “rheumatoid arthritis” or osteoarthritis or “kidney disease?” or “kidney disorder?” or epilep* or seizure? or hiv or “acquired immun* deficiency syndrome?” or aids or “liver disease?” or “liver disorder?” or stroke* or dementia or “cardiac arrhythmia*” or an?emia* or obesity or obese or “prostatic hyperplasia” or “prostatic hypertrophy” or tuberculosis or “chronic hepatitis” or “substance-related disorder?” or “substance abuse” or “drug abuse” or “marijuana abuse” or “cocaine abuse” or “amphetamine abuse” or addiction? or “inflammatory bowel disease?” or “ulcerative colitis” or crohn or ibd or deaf* or blind* or “visual loss” or parkinson* or autis*)) ) OR AB ( ((asthma*) AND (((mental or anxiety or mood or psychological) N1 (disease? or disorder?)) or “sleep disorder” or depression? or osteoporosis or dyslipid?emia* or hyperlipid?emia* or hypercholesterolemia* or hypertriglyceridemia* or “thyroid disease?” or “thyroid disorder?” or hyperthyroid* or hypothyroid* or “joint disease*” or “rheumatoid arthritis” or osteoarthritis or “kidney disease?” or “kidney disorder?” or epilep* or seizure? or hiv or “acquired immun* deficiency syndrome?” or aids or “liver disease?” or “liver disorder?” or stroke* or dementia or “cardiac arrhythmia*” or an?emia* or obesity or obese or “prostatic hyperplasia” or “prostatic hypertrophy” or tuberculosis or “chronic hepatitis” or “substance-related disorder?” or “substance abuse” or “drug abuse” or “marijuana abuse” or “cocaine abuse” or “amphetamine abuse” or addiction? or “inflammatory bowel disease?” or “ulcerative colitis” or crohn or ibd or deaf* or blind* or “visual loss” or parkinson* or autis*)) ) | Limiters - Articles on Several Companies  Expanders - Apply equivalent subjects  Search modes - Boolean/Phrase | Interface - EBSCOhost Research Databases  Search Screen - Advanced Search  Database - Business Source Complete | 32 |
| S7 | TI ( ((“heart failure” or “cardiac failure” or “myocardial failure”) and (asthma* or ((mental or anxiety or mood or psychological) N1 (disease? or disorder?)) or “sleep disorder” or depression? or osteoporosis or dyslipid?emia* or hyperlipid?emia* or hypercholesterolemia* or hypertriglyceridemia* or “thyroid disease?” or “thyroid disorder?” or hyperthyroid* or hypothyroid* or “joint disease*” or “rheumatoid arthritis” or osteoarthritis or “kidney disease?” or “kidney disorder?” or epilep* or seizure? or hiv or “acquired immun* deficiency syndrome?” or aids or “liver disease?” or “liver disorder?” or stroke* or dementia or “cardiac arrhythmia*” or an?emia* or obesity or obese or “prostatic hyperplasia” or “prostatic hypertrophy” or tuberculosis or “chronic hepatitis” or “substance-related disorder?” or “substance abuse” or “drug abuse” or “marijuana abuse” or “cocaine abuse” or “amphetamine abuse” or addiction? or “inflammatory bowel disease?” or “ulcerative colitis” or crohn or ibd or deaf* or blind* or “visual loss” or parkinson* or autis*)) ) OR AB ( ((“heart failure” or “cardiac failure” or “myocardial failure”) and (asthma* or ((mental or anxiety or mood or psychological) N1 (disease? or disorder?)) or “sleep disorder” or depression? or osteoporosis or dyslipid?emia* or hyperlipid?emia* or hypercholesterolemia* or hypertriglyceridemia* or “thyroid disease?” or “thyroid disorder?” or hyperthyroid* or hypothyroid* or “joint disease*” or “rheumatoid arthritis” or osteoarthritis or “kidney disease?” or “kidney disorder?” or epilep* or seizure? or hiv or “acquired immun* deficiency syndrome?” or aids or “liver disease?” or “liver disorder?” or stroke* or dementia or “cardiac arrhythmia*” or an?emia* or obesity or obese or “prostatic hyperplasia” or “prostatic hypertrophy” or tuberculosis or “chronic hepatitis” or “substance-related disorder?” or “substance abuse” or “drug abuse” or “marijuana abuse” or “cocaine abuse” or “amphetamine abuse” or addiction? or “inflammatory bowel disease?” or “ulcerative colitis” or crohn or ibd or deaf* or blind* or “visual loss” or parkinson* or autis*)) ) | Limiters - Articles on Several Companies  Expanders - Apply equivalent subjects  Search modes - Boolean/Phrase | Interface - EBSCOhost Research Databases  Search Screen - Advanced Search  Database - Business Source Complete | 20 |
| S6 | TI ( ((“myocardial infarct*” or “cardiovascular strok*”) and (“heart failure” or “cardiac failure” or “myocardial failure” or asthma* or ((mental or anxiety or mood or psychological) N1 (disease? or disorder?)) or “sleep disorder” or depression? or osteoporosis or dyslipid?emia* or hyperlipid?emia* or hypercholesterolemia* or hypertriglyceridemia* or “thyroid disease?” or “thyroid disorder?” or hyperthyroid* or hypothyroid* or “joint disease*” or “rheumatoid arthritis” or osteoarthritis or “kidney disease?” or “kidney disorder?” or epilep* or seizure? or hiv or “acquired immun* deficiency syndrome?” or aids or “liver disease?” or “liver disorder?” or stroke* or dementia or “cardiac arrhythmia*” or an?emia* or obesity or obese or “prostatic hyperplasia” or “prostatic hypertrophy” or tuberculosis or “chronic hepatitis” or “substance-related disorder?” or “substance abuse” or “drug abuse” or “marijuana abuse” or “cocaine abuse” or “amphetamine abuse” or addiction? or “inflammatory bowel disease?” or “ulcerative colitis” or crohn or ibd or deaf* or blind* or “visual loss” or parkinson* or autis*)) ) OR AB ( ((“myocardial infarct*” or “cardiovascular strok*”) and (“heart failure” or “cardiac failure” or “myocardial failure” or asthma* or ((mental or anxiety or mood or psychological) N1 (disease? or disorder?)) or “sleep disorder” or depression? or osteoporosis or dyslipid?emia* or hyperlipid?emia* or hypercholesterolemia* or hypertriglyceridemia* or “thyroid disease?” or “thyroid disorder?” or hyperthyroid* or hypothyroid* or “joint disease*” or “rheumatoid arthritis” or osteoarthritis or “kidney disease?” or “kidney disorder?” or epilep* or seizure? or hiv or “acquired immun* deficiency syndrome?” or aids or “liver disease?” or “liver disorder?” or stroke* or dementia or “cardiac arrhythmia*” or an?emia* or obesity or obese or “prostatic hyperplasia” or “prostatic hypertrophy” or tuberculosis or “chronic hepatitis” or “substance-related disorder?” or “substance abuse” or “drug abuse” or “marijuana abuse” or “cocaine abuse” or “amphetamine abuse” or addiction? or “inflammatory bowel disease?” or “ulcerative colitis” or crohn or ibd or deaf* or blind* or “visual loss” or parkinson* or autis*)) ) | Limiters - Articles on Several Companies  Expanders - Apply equivalent subjects  Search modes - Boolean/Phrase | Interface - EBSCOhost Research Databases  Search Screen - Advanced Search  Database - Business Source Complete | 6 |
| S5 | TI ( (((cardiac or cardiovascular or coronary) N1 (disease? or disorder? or failure)) AND (“myocardial infarct*” or “cardiovascular strok*” or “heart failure” or “cardiac failure” or “myocardial failure” or asthma* or ((mental or anxiety or mood or psychological) N1 (disease? or disorder?)) or “sleep disorder” or depression? or osteoporosis or dyslipid?emia* or hyperlipid?emia* or hypercholesterolemia* or hypertriglyceridemia* or “thyroid disease?” or “thyroid disorder?” or hyperthyroid* or hypothyroid* or “joint disease*” or “rheumatoid arthritis” or osteoarthritis or “kidney disease?” or “kidney disorder?” or epilep* or seizure? or hiv or “acquired immun* deficiency syndrome?” or aids or “liver disease?” or “liver disorder?” or stroke* or dementia or “cardiac arrhythmia*” or an?emia* or obesity or obese or “prostatic hyperplasia” or “prostatic hypertrophy” or tuberculosis or “chronic hepatitis” or “substance-related disorder?” or “substance abuse” or “drug abuse” or “marijuana abuse” or “cocaine abuse” or “amphetamine abuse” or addiction? or “inflammatory bowel disease?” or “ulcerative colitis” or crohn or ibd or deaf* or blind* or “visual loss” or parkinson* or autis*)) ) OR AB ( (((cardiac or cardiovascular or coronary) N1 (disease? or disorder? or failure)) AND (“myocardial infarct*” or “cardiovascular strok*” or “heart failure” or “cardiac failure” or “myocardial failure” or asthma* or ((mental or anxiety or mood or psychological) N1 (disease? or disorder?)) or “sleep disorder” or depression? or osteoporosis or dyslipid?emia* or hyperlipid?emia* or hypercholesterolemia* or hypertriglyceridemia* or “thyroid disease?” or “thyroid disorder?” or hyperthyroid* or hypothyroid* or “joint disease*” or “rheumatoid arthritis” or osteoarthritis or “kidney disease?” or “kidney disorder?” or epilep* or seizure? or hiv or “acquired immun* deficiency syndrome?” or aids or “liver disease?” or “liver disorder?” or stroke* or dementia or “cardiac arrhythmia*” or an?emia* or obesity or obese or “prostatic hyperplasia” or “prostatic hypertrophy” or tuberculosis or “chronic hepatitis” or “substance-related disorder?” or “substance abuse” or “drug abuse” or “marijuana abuse” or “cocaine abuse” or “amphetamine abuse” or addiction? or “inflammatory bowel disease?” or “ulcerative colitis” or crohn or ibd or deaf* or blind* or “visual loss” or parkinson* or autis*)) ) | Limiters - Articles on Several Companies  Expanders - Apply equivalent subjects  Search modes - Boolean/Phrase | Interface - EBSCOhost Research Databases  Search Screen - Advanced Search  Database - Business Source Complete | 48 |
| S4 | TI ( ((neoplasm? or cancer?) AND (((cardiac or cardiovascular or coronary) N1 (disease? or disorder? or failure)) or “myocardial infarct*” or “cardiovascular strok*” or “heart failure” or “cardiac failure” or “myocardial failure” or asthma* or ((mental or anxiety or mood or psychological) N1 (disease? or disorder?)) or “sleep disorder” or depression? or osteoporosis or dyslipid?emia* or hyperlipid?emia* or hypercholesterolemia* or hypertriglyceridemia* or “thyroid disease?” or “thyroid disorder?” or hyperthyroid* or hypothyroid* or “joint disease*” or “rheumatoid arthritis” or osteoarthritis or “kidney disease?” or “kidney disorder?” or epilep* or seizure? or hiv or “acquired immun* deficiency syndrome?” or aids or “liver disease?” or “liver disorder?” or stroke* or dementia or “cardiac arrhythmia*” or an?emia* or obesity or obese or “prostatic hyperplasia” or “prostatic hypertrophy” or tuberculosis or “chronic hepatitis” or “substance-related disorder?” or “substance abuse” or “drug abuse” or “marijuana abuse” or “cocaine abuse” or “amphetamine abuse” or addiction? or “inflammatory bowel disease?” or “ulcerative colitis” or crohn or ibd or deaf* or blind* or “visual loss” or parkinson* or autis*)) ) OR AB ( ((neoplasm? or cancer?) AND (((cardiac or cardiovascular or coronary) N1 (disease? or disorder? or failure)) or “myocardial infarct*” or “cardiovascular strok*” or “heart failure” or “cardiac failure” or “myocardial failure” or asthma* or ((mental or anxiety or mood or psychological) N1 (disease? or disorder?)) or “sleep disorder” or depression? or osteoporosis or dyslipid?emia* or hyperlipid?emia* or hypercholesterolemia* or hypertriglyceridemia* or “thyroid disease?” or “thyroid disorder?” or hyperthyroid* or hypothyroid* or “joint disease*” or “rheumatoid arthritis” or osteoarthritis or “kidney disease?” or “kidney disorder?” or epilep* or seizure? or hiv or “acquired immun* deficiency syndrome?” or aids or “liver disease?” or “liver disorder?” or stroke* or dementia or “cardiac arrhythmia*” or an?emia* or obesity or obese or “prostatic hyperplasia” or “prostatic hypertrophy” or tuberculosis or “chronic hepatitis” or “substance-related disorder?” or “substance abuse” or “drug abuse” or “marijuana abuse” or “cocaine abuse” or “amphetamine abuse” or addiction? or “inflammatory bowel disease?” or “ulcerative colitis” or crohn or ibd or deaf* or blind* or “visual loss” or parkinson* or autis*)) ) | Limiters - Articles on Several Companies  Expanders - Apply equivalent subjects  Search modes - Boolean/Phrase | Interface - EBSCOhost Research Databases  Search Screen - Advanced Search  Database - Business Source Complete | 413 |
| S3 | TI ( ((hypertens* or "high blood pressure*") AND (neoplasm? or cancer? or ((cardiac or cardiovascular or coronary) N1 (disease? or disorder? or failure)) or “myocardial infarct*” or “cardiovascular strok*” or “heart failure” or “cardiac failure” or “myocardial failure” or asthma* or ((mental or anxiety or mood or psychological) N1 (disease? or disorder?)) or “sleep disorder” or depression? or osteoporosis or dyslipid?emia* or hyperlipid?emia* or hypercholesterolemia* or hypertriglyceridemia* or “thyroid disease?” or “thyroid disorder?” or hyperthyroid* or hypothyroid* or “joint disease*” or “rheumatoid arthritis” or osteoarthritis or “kidney disease?” or “kidney disorder?” or epilep* or seizure? or hiv or “acquired immun* deficiency syndrome?” or aids or “liver disease?” or “liver disorder?” or stroke* or dementia or “cardiac arrhythmia*” or an?emia* or obesity or obese or “prostatic hyperplasia” or “prostatic hypertrophy” or tuberculosis or “chronic hepatitis” or “substance-related disorder?” or “substance abuse” or “drug abuse” or “marijuana abuse” or “cocaine abuse” or “amphetamine abuse” or addiction? or “inflammatory bowel disease?” or “ulcerative colitis” or crohn or ibd or deaf* or blind* or “visual loss” or parkinson* or autis*)) ) OR AB ( ((hypertens* or "high blood pressure*") AND (neoplasm? or cancer? or ((cardiac or cardiovascular or coronary) N1 (disease? or disorder? or failure)) or “myocardial infarct*” or “cardiovascular strok*” or “heart failure” or “cardiac failure” or “myocardial failure” or asthma* or ((mental or anxiety or mood or psychological) N1 (disease? or disorder?)) or “sleep disorder” or depression? or osteoporosis or dyslipid?emia* or hyperlipid?emia* or hypercholesterolemia* or hypertriglyceridemia* or “thyroid disease?” or “thyroid disorder?” or hyperthyroid* or hypothyroid* or “joint disease*” or “rheumatoid arthritis” or osteoarthritis or “kidney disease?” or “kidney disorder?” or epilep* or seizure? or hiv or “acquired immun* deficiency syndrome?” or aids or “liver disease?” or “liver disorder?” or stroke* or dementia or “cardiac arrhythmia*” or an?emia* or obesity or obese or “prostatic hyperplasia” or “prostatic hypertrophy” or tuberculosis or “chronic hepatitis” or “substance-related disorder?” or “substance abuse” or “drug abuse” or “marijuana abuse” or “cocaine abuse” or “amphetamine abuse” or addiction? or “inflammatory bowel disease?” or “ulcerative colitis” or crohn or ibd or deaf* or blind* or “visual loss” or parkinson* or autis*)) ) | Limiters - Articles on Several Companies  Expanders - Apply equivalent subjects  Search modes - Boolean/Phrase | Interface - EBSCOhost Research Databases  Search Screen - Advanced Search  Database - Business Source Complete | 84 |
| S2 | TI ( ((diabet*) AND (hypertens* or "high blood pressure*" or neoplasm? or cancer? or ((cardiac or cardiovascular or coronary) N1 (disease? or disorder? or failure)) or “myocardial infarct*” or “cardiovascular strok*” or “heart failure” or “cardiac failure” or “myocardial failure” or asthma* or ((mental or anxiety or mood or psychological) N1 (disease? or disorder?)) or “sleep disorder” or depression? or osteoporosis or dyslipid?emia* or hyperlipid?emia* or hypercholesterolemia* or hypertriglyceridemia* or “thyroid disease?” or “thyroid disorder?” or hyperthyroid* or hypothyroid* or “joint disease*” or “rheumatoid arthritis” or osteoarthritis or “kidney disease?” or “kidney disorder?” or epilep* or seizure? or hiv or “acquired immun* deficiency syndrome?” or aids or “liver disease?” or “liver disorder?” or stroke* or dementia or “cardiac arrhythmia*” or an?emia* or obesity or obese or “prostatic hyperplasia” or “prostatic hypertrophy” or tuberculosis or “chronic hepatitis” or “substance-related disorder?” or “substance abuse” or “drug abuse” or “marijuana abuse” or “cocaine abuse” or “amphetamine abuse” or addiction? or “inflammatory bowel disease?” or “ulcerative colitis” or crohn or ibd or deaf* or blind* or “visual loss” or parkinson* or autis*)) ) OR AB ( ((diabet*) AND (hypertens* or "high blood pressure*" or neoplasm? or cancer? or ((cardiac or cardiovascular or coronary) N1 (disease? or disorder? or failure)) or “myocardial infarct*” or “cardiovascular strok*” or “heart failure” or “cardiac failure” or “myocardial failure” or asthma* or ((mental or anxiety or mood or psychological) N1 (disease? or disorder?)) or “sleep disorder” or depression? or osteoporosis or dyslipid?emia* or hyperlipid?emia* or hypercholesterolemia* or hypertriglyceridemia* or “thyroid disease?” or “thyroid disorder?” or hyperthyroid* or hypothyroid* or “joint disease*” or “rheumatoid arthritis” or osteoarthritis or “kidney disease?” or “kidney disorder?” or epilep* or seizure? or hiv or “acquired immun* deficiency syndrome?” or aids or “liver disease?” or “liver disorder?” or stroke* or dementia or “cardiac arrhythmia*” or an?emia* or obesity or obese or “prostatic hyperplasia” or “prostatic hypertrophy” or tuberculosis or “chronic hepatitis” or “substance-related disorder?” or “substance abuse” or “drug abuse” or “marijuana abuse” or “cocaine abuse” or “amphetamine abuse” or addiction? or “inflammatory bowel disease?” or “ulcerative colitis” or crohn or ibd or deaf* or blind* or “visual loss” or parkinson* or autis*)) ) | Limiters - Articles on Several Companies  Expanders - Apply equivalent subjects  Search modes - Boolean/Phrase | Interface - EBSCOhost Research Databases  Search Screen - Advanced Search  Database - Business Source Complete | 348 |
| S1 | TI ( ((“chronic obstructive pulmonary disease” or copd or “pulmonary disease?” or “pulmonary disorder?”) and (diabet* or hypertens* or "high blood pressure*" or neoplasm? or cancer? or ((cardiac or cardiovascular or coronary) N1 (disease? or disorder? or failure)) or “myocardial infarct*” or “cardiovascular strok*” or “heart failure” or “cardiac failure” or “myocardial failure” or asthma* or ((mental or anxiety or mood or psychological) N1 (disease? or disorder?)) or “sleep disorder” or depression? or osteoporosis or dyslipid?emia* or hyperlipid?emia* or hypercholesterolemia* or hypertriglyceridemia* or “thyroid disease?” or “thyroid disorder?” or hyperthyroid* or hypothyroid* or “joint disease*” or “rheumatoid arthritis” or osteoarthritis or “kidney disease?” or “kidney disorder?” or epilep* or seizure? or hiv or “acquired immun* deficiency syndrome?” or aids or “liver disease?” or “liver disorder?” or stroke* or dementia or “cardiac arrhythmia*” or an?emia* or obesity or obese or “prostatic hyperplasia” or “prostatic hypertrophy” or tuberculosis or “chronic hepatitis” or “substance-related disorder?” or “substance abuse” or “drug abuse” or “marijuana abuse” or “cocaine abuse” or “amphetamine abuse” or addiction? or “inflammatory bowel disease?” or “ulcerative colitis” or crohn or ibd or deaf* or blind* or “visual loss” or parkinson* or autis*)) ) OR AB ( ((“chronic obstructive pulmonary disease” or copd or “pulmonary disease?” or “pulmonary disorder?”) and (diabet* or hypertens* or "high blood pressure*" or neoplasm? or cancer? or ((cardiac or cardiovascular or coronary) N1 (disease? or disorder? or failure)) or “myocardial infarct*” or “cardiovascular strok*” or “heart failure” or “cardiac failure” or “myocardial failure” or asthma* or ((mental or anxiety or mood or psychological) N1 (disease? or disorder?)) or “sleep disorder” or depression? or osteoporosis or dyslipid?emia* or hyperlipid?emia* or hypercholesterolemia* or hypertriglyceridemia* or “thyroid disease?” or “thyroid disorder?” or hyperthyroid* or hypothyroid* or “joint disease*” or “rheumatoid arthritis” or osteoarthritis or “kidney disease?” or “kidney disorder?” or epilep* or seizure? or hiv or “acquired immun* deficiency syndrome?” or aids or “liver disease?” or “liver disorder?” or stroke* or dementia or “cardiac arrhythmia*” or an?emia* or obesity or obese or “prostatic hyperplasia” or “prostatic hypertrophy” or tuberculosis or “chronic hepatitis” or “substance-related disorder?” or “substance abuse” or “drug abuse” or “marijuana abuse” or “cocaine abuse” or “amphetamine abuse” or addiction? or “inflammatory bowel disease?” or “ulcerative colitis” or crohn or ibd or deaf* or blind* or “visual loss” or parkinson* or autis*)) ) | Limiters - Articles on Several Companies  Expanders - Apply equivalent subjects  Search modes - Boolean/Phrase | Interface - EBSCOhost Research Databases  Search Screen - Advanced Search  Database - Business Source Complete | 45 |

**Executed search Web of Science**

| # 39 | [4,988](https://apps.webofknowledge.com/summary.do?product=WOS&doc=1&qid=42&SID=F6DCGio8oZtFJuIldhR&search_mode=CombineSearches&update_back2search_link_param=yes) | #37  AND  #34  AND  #31  **Refined by:** **PUBLICATION YEARS:** ( 2020 OR 2012 OR 2019 OR 2011 OR 2018 OR 2010 OR 2017 OR 2016 OR 2015 OR 2014 OR 2013 )  Indexes=SCI-EXPANDED, SSCI, A&HCI, CPCI-S, CPCI-SSH, ESCI Timespan=All years |  |  |  |
| --- | --- | --- | --- | --- | --- |
|  | | | | | |
| # 38 | [6,233](https://apps.webofknowledge.com/summary.do?product=WOS&doc=1&qid=41&SID=F6DCGio8oZtFJuIldhR&search_mode=CombineSearches&update_back2search_link_param=yes) | #37  AND  #34  AND  #31  Indexes=SCI-EXPANDED, SSCI, A&HCI, CPCI-S, CPCI-SSH, ESCI Timespan=All years | [Edit](https://apps.webofknowledge.com/WOS_AdvancedSearch_input.do?product=WOS&SID=F6DCGio8oZtFJuIldhR&search_mode=AdvancedSearch&replaceSetId=38&editState=init) |  |  |
|  | | | | | |
| # 37 | [2,256,867](https://apps.webofknowledge.com/summary.do?product=WOS&doc=1&qid=40&SID=F6DCGio8oZtFJuIldhR&search_mode=CombineSearches&update_back2search_link_param=yes) | #36  OR  #35  Indexes=SCI-EXPANDED, SSCI, A&HCI, CPCI-S, CPCI-SSH, ESCI Timespan=All years | [Edit](https://apps.webofknowledge.com/WOS_AdvancedSearch_input.do?product=WOS&SID=F6DCGio8oZtFJuIldhR&search_mode=AdvancedSearch&replaceSetId=37&editState=init) |  |  |
|  | | | | | |
| # 36 | [487,846](https://apps.webofknowledge.com/summary.do?product=WOS&doc=1&qid=39&SID=F6DCGio8oZtFJuIldhR&search_mode=AdvancedSearch&update_back2search_link_param=yes) | TS=(((resource*  or  "health  service")  NEAR/3  ("use" or utilisation or utilization) )  or  ((financial or economic)  NEAR/3  (burden or impact or consequence) )  or  spending  or  “out  of  pocket”  or  out-of-pocket  or  oop  or  insurance  or  absenteeism  or  (productivity NEAR/3 (loss or lost) )  or  "economic  modelling")  Indexes=SCI-EXPANDED, SSCI, A&HCI, CPCI-S, CPCI-SSH, ESCI Timespan=All years | [Edit](https://apps.webofknowledge.com/WOS_AdvancedSearch_input.do?product=WOS&SID=F6DCGio8oZtFJuIldhR&search_mode=AdvancedSearch&replaceSetId=36&editState=init) |  |  |
|  | | | | | |
| # 35 | [1,873,109](https://apps.webofknowledge.com/summary.do?product=WOS&doc=1&qid=38&SID=F6DCGio8oZtFJuIldhR&search_mode=AdvancedSearch&update_back2search_link_param=yes) | TS=(cost*  or  ((health or health-care or healthcare or medical)  NEAR/3  expenditure*)  or  expense*  or  “hospital  resource*”)  Indexes=SCI-EXPANDED, SSCI, A&HCI, CPCI-S, CPCI-SSH, ESCI Timespan=All years | [Edit](https://apps.webofknowledge.com/WOS_AdvancedSearch_input.do?product=WOS&SID=F6DCGio8oZtFJuIldhR&search_mode=AdvancedSearch&replaceSetId=35&editState=init) |  |  |
|  | | | | | |
| # 34 | [427,157](https://apps.webofknowledge.com/summary.do?product=WOS&doc=1&qid=37&SID=F6DCGio8oZtFJuIldhR&search_mode=CombineSearches&update_back2search_link_param=yes) | #33  OR  #32  Indexes=SCI-EXPANDED, SSCI, A&HCI, CPCI-S, CPCI-SSH, ESCI Timespan=All years | [Edit](https://apps.webofknowledge.com/WOS_AdvancedSearch_input.do?product=WOS&SID=F6DCGio8oZtFJuIldhR&search_mode=AdvancedSearch&replaceSetId=34&editState=init) |  |  |
|  | | | | | |
| # 33 | [178,490](https://apps.webofknowledge.com/summary.do?product=WOS&doc=1&qid=36&SID=F6DCGio8oZtFJuIldhR&search_mode=AdvancedSearch&update_back2search_link_param=yes) | TS=(comorbidit*  or  co-morbidit*  or  multidisease*  or  multi-disease*  or  multimorbidit*  or  multi-morbidit*  or  multipatholog*  or  multi-patholog*  or  pluripatholog*  or  polypatholog*  or  poly-pathology*)  Indexes=SCI-EXPANDED, SSCI, A&HCI, CPCI-S, CPCI-SSH, ESCI Timespan=All years | [Edit](https://apps.webofknowledge.com/WOS_AdvancedSearch_input.do?product=WOS&SID=F6DCGio8oZtFJuIldhR&search_mode=AdvancedSearch&replaceSetId=33&editState=init) |  |  |
|  | | | | | |
| # 32 | [267,885](https://apps.webofknowledge.com/summary.do?product=WOS&doc=1&qid=33&SID=F6DCGio8oZtFJuIldhR&search_mode=AdvancedSearch&update_back2search_link_param=yes) | TS=((condition*  or  diagnos*  or  disease*  or  illness*  or  “health  problem*”  or  patholog*  or  disorder*  or  syndrome*)  NEAR/1  (associated or coexisting or co-existing or comorbid or co-morbid or concurrent or co-occuring or cooccuring or multiple)  or  “associated  morbidit*”)  Indexes=SCI-EXPANDED, SSCI, A&HCI, CPCI-S, CPCI-SSH, ESCI Timespan=All years | [Edit](https://apps.webofknowledge.com/WOS_AdvancedSearch_input.do?product=WOS&SID=F6DCGio8oZtFJuIldhR&search_mode=AdvancedSearch&replaceSetId=32&editState=init) |  |  |
|  | | | | | |
| # 31 | [779,780](https://apps.webofknowledge.com/summary.do?product=WOS&doc=1&qid=32&SID=F6DCGio8oZtFJuIldhR&search_mode=CombineSearches&update_back2search_link_param=yes) | #30  OR  #29  OR  #28  OR  #27  OR  #26  OR  #25  OR  #24  OR  #23  OR  #22  OR  #21  OR  #20  OR  #19  OR  #18  OR  #17  OR  #16  OR  #15  OR  #14  OR  #13  OR  #12  OR  #11  OR  #10  OR  #9  OR  #8  OR  #7  OR  #6  OR  #5  OR  #4  OR  #3  OR  #2  OR  #1  Indexes=SCI-EXPANDED, SSCI, A&HCI, CPCI-S, CPCI-SSH, ESCI Timespan=All years | [Edit](https://apps.webofknowledge.com/WOS_AdvancedSearch_input.do?product=WOS&SID=F6DCGio8oZtFJuIldhR&search_mode=AdvancedSearch&replaceSetId=31&editState=init) |  |  |
|  | | | | | |
| # 30 | [742](https://apps.webofknowledge.com/summary.do?product=WOS&doc=1&qid=31&SID=F6DCGio8oZtFJuIldhR&search_mode=AdvancedSearch&update_back2search_link_param=yes) | TS=(parkinson*  and  autis*)  Indexes=SCI-EXPANDED, SSCI, A&HCI, CPCI-S, CPCI-SSH, ESCI Timespan=All years | [Edit](https://apps.webofknowledge.com/WOS_AdvancedSearch_input.do?product=WOS&SID=F6DCGio8oZtFJuIldhR&search_mode=AdvancedSearch&replaceSetId=30&editState=init) |  |  |
|  | | | | | |
| # 29 | [8,452](https://apps.webofknowledge.com/summary.do?product=WOS&doc=1&qid=30&SID=F6DCGio8oZtFJuIldhR&search_mode=AdvancedSearch&update_back2search_link_param=yes) | TS=((blind*  or  “visual  loss”)  and  (parkinson* or autis*) )  Indexes=SCI-EXPANDED, SSCI, A&HCI, CPCI-S, CPCI-SSH, ESCI Timespan=All years | [Edit](https://apps.webofknowledge.com/WOS_AdvancedSearch_input.do?product=WOS&SID=F6DCGio8oZtFJuIldhR&search_mode=AdvancedSearch&replaceSetId=29&editState=init) |  |  |
|  | | | | | |
| # 28 | [3,043](https://apps.webofknowledge.com/summary.do?product=WOS&doc=1&qid=29&SID=F6DCGio8oZtFJuIldhR&search_mode=AdvancedSearch&update_back2search_link_param=yes) | TS=((deaf*)  and  (blind* or “visual loss” or parkinson* or autis*) )  Indexes=SCI-EXPANDED, SSCI, A&HCI, CPCI-S, CPCI-SSH, ESCI Timespan=All years | [Edit](https://apps.webofknowledge.com/WOS_AdvancedSearch_input.do?product=WOS&SID=F6DCGio8oZtFJuIldhR&search_mode=AdvancedSearch&replaceSetId=28&editState=init) |  |  |
|  | | | | | |
| # 27 | [5,089](https://apps.webofknowledge.com/summary.do?product=WOS&doc=1&qid=28&SID=F6DCGio8oZtFJuIldhR&search_mode=AdvancedSearch&update_back2search_link_param=yes) | TS=((“inflammatory  bowel  disease?”  or  “ulcerative  colitis”  or  crohn  or  ibd)  and  (deaf* or blind* or “visual loss” or parkinson* or autis*) )  Indexes=SCI-EXPANDED, SSCI, A&HCI, CPCI-S, CPCI-SSH, ESCI Timespan=All years | [Edit](https://apps.webofknowledge.com/WOS_AdvancedSearch_input.do?product=WOS&SID=F6DCGio8oZtFJuIldhR&search_mode=AdvancedSearch&replaceSetId=27&editState=init) |  |  |
|  | | | | | |
| # 26 | [2,129](https://apps.webofknowledge.com/summary.do?product=WOS&doc=1&qid=27&SID=F6DCGio8oZtFJuIldhR&search_mode=AdvancedSearch&update_back2search_link_param=yes) | TS=((“substance-related  disorder?”  or  “substance  abuse”  or  “drug  abuse”  or  “marijuana  abuse”  or  “cocaine  abuse”  or  “amphetamine  abuse”  or  addiction?)  and  (“inflammatory bowel disease?” or “ulcerative colitis” or crohn or ibd or deaf* or blind* or “visual loss” or parkinson* or autis*) )  Indexes=SCI-EXPANDED, SSCI, A&HCI, CPCI-S, CPCI-SSH, ESCI Timespan=All years | [Edit](https://apps.webofknowledge.com/WOS_AdvancedSearch_input.do?product=WOS&SID=F6DCGio8oZtFJuIldhR&search_mode=AdvancedSearch&replaceSetId=26&editState=init) |  |  |
|  | | | | | |
| # 25 | [1,945](https://apps.webofknowledge.com/summary.do?product=WOS&doc=1&qid=26&SID=F6DCGio8oZtFJuIldhR&search_mode=AdvancedSearch&update_back2search_link_param=yes) | TS=((“chronic  hepatitis”)  and  (“substance-related disorder?” or “substance abuse” or “drug abuse” or “marijuana abuse” or “cocaine abuse” or “amphetamine abuse” or addiction? or “inflammatory bowel disease?” or “ulcerative colitis” or crohn or ibd or deaf* or blind* or “visual loss” or parkinson* or autis*) )  Indexes=SCI-EXPANDED, SSCI, A&HCI, CPCI-S, CPCI-SSH, ESCI Timespan=All years | [Edit](https://apps.webofknowledge.com/WOS_AdvancedSearch_input.do?product=WOS&SID=F6DCGio8oZtFJuIldhR&search_mode=AdvancedSearch&replaceSetId=25&editState=init) |  |  |
|  | | | | | |
| # 24 | [3,541](https://apps.webofknowledge.com/summary.do?product=WOS&doc=1&qid=25&SID=F6DCGio8oZtFJuIldhR&search_mode=AdvancedSearch&update_back2search_link_param=yes) | TS=((tuberculosis)  and  (“chronic hepatitis” or “substance-related disorder?” or “substance abuse” or “drug abuse” or “marijuana abuse” or “cocaine abuse” or “amphetamine abuse” or addiction? or “inflammatory bowel disease?” or “ulcerative colitis” or crohn or ibd or deaf* or blind* or “visual loss” or parkinson* or autis*) )  Indexes=SCI-EXPANDED, SSCI, A&HCI, CPCI-S, CPCI-SSH, ESCI Timespan=All years | [Edit](https://apps.webofknowledge.com/WOS_AdvancedSearch_input.do?product=WOS&SID=F6DCGio8oZtFJuIldhR&search_mode=AdvancedSearch&replaceSetId=24&editState=init) |  |  |
|  | | | | | |
| # 23 | [1,420](https://apps.webofknowledge.com/summary.do?product=WOS&doc=1&qid=24&SID=F6DCGio8oZtFJuIldhR&search_mode=AdvancedSearch&update_back2search_link_param=yes) | TS=((“prostatic  hyperplasia”  or  “prostatic  hypertrophy”)  and  (tuberculosis or “chronic hepatitis” or “substance-related disorder?” or “substance abuse” or “drug abuse” or “marijuana abuse” or “cocaine abuse” or “amphetamine abuse” or addiction? or “inflammatory bowel disease?” or “ulcerative colitis” or crohn or ibd or deaf* or blind* or “visual loss” or parkinson* or autis*) )  Indexes=SCI-EXPANDED, SSCI, A&HCI, CPCI-S, CPCI-SSH, ESCI Timespan=All years | [Edit](https://apps.webofknowledge.com/WOS_AdvancedSearch_input.do?product=WOS&SID=F6DCGio8oZtFJuIldhR&search_mode=AdvancedSearch&replaceSetId=23&editState=init) |  |  |
|  | | | | | |
| # 22 | [13,242](https://apps.webofknowledge.com/summary.do?product=WOS&doc=1&qid=23&SID=F6DCGio8oZtFJuIldhR&search_mode=AdvancedSearch&update_back2search_link_param=yes) | TS=((obesity  or  obese)  and  ("prostatic hyperplasia" or "prostatic hypertrophy" or tuberculosis or "chronic hepatitis" or "substance-related disorder?" or "substance abuse" or "drug abuse" or "marijuana abuse" or "cocaine abuse" or "amphetamine abuse" or addiction? or "inflammatory bowel disease?" or "ulcerative colitis" or crohn or ibd or deaf* or blind* or "visual loss" or parkinson* or autis*) )  Indexes=SCI-EXPANDED, SSCI, A&HCI, CPCI-S, CPCI-SSH, ESCI Timespan=All years | [Edit](https://apps.webofknowledge.com/WOS_AdvancedSearch_input.do?product=WOS&SID=F6DCGio8oZtFJuIldhR&search_mode=AdvancedSearch&replaceSetId=22&editState=init) |  |  |
|  | | | | | |
| # 21 | [2,089](https://apps.webofknowledge.com/summary.do?product=WOS&doc=1&qid=22&SID=F6DCGio8oZtFJuIldhR&search_mode=AdvancedSearch&update_back2search_link_param=yes) | TS=((an?emia*)  and  (obesity or obese or “prostatic hyperplasia” or “prostatic hypertrophy” or tuberculosis or “chronic hepatitis” or “substance-related disorder?” or “substance abuse” or “drug abuse” or “marijuana abuse” or “cocaine abuse” or “amphetamine abuse” or addiction? or “inflammatory bowel disease?” or “ulcerative colitis” or crohn or ibd or deaf* or blind* or “visual loss” or parkinson* or autis*) )  Indexes=SCI-EXPANDED, SSCI, A&HCI, CPCI-S, CPCI-SSH, ESCI Timespan=All years | [Edit](https://apps.webofknowledge.com/WOS_AdvancedSearch_input.do?product=WOS&SID=F6DCGio8oZtFJuIldhR&search_mode=AdvancedSearch&replaceSetId=21&editState=init) |  |  |
|  | | | | | |
| # 20 | [1,148](https://apps.webofknowledge.com/summary.do?product=WOS&doc=1&qid=21&SID=F6DCGio8oZtFJuIldhR&search_mode=AdvancedSearch&update_back2search_link_param=yes) | TS=((“cardiac  arrhythmia*”)  and  (an?emia* or obesity or obese or “prostatic hyperplasia” or “prostatic hypertrophy” or tuberculosis or “chronic hepatitis” or “substance-related disorder?” or “substance abuse” or “drug abuse” or “marijuana abuse” or “cocaine abuse” or “amphetamine abuse” or addiction? or “inflammatory bowel disease?” or “ulcerative colitis” or crohn or ibd or deaf* or blind* or “visual loss” or parkinson* or autis*) )  Indexes=SCI-EXPANDED, SSCI, A&HCI, CPCI-S, CPCI-SSH, ESCI Timespan=All years | [Edit](https://apps.webofknowledge.com/WOS_AdvancedSearch_input.do?product=WOS&SID=F6DCGio8oZtFJuIldhR&search_mode=AdvancedSearch&replaceSetId=20&editState=init) |  |  |
|  | | | | | |
| # 19 | [26,947](https://apps.webofknowledge.com/summary.do?product=WOS&doc=1&qid=20&SID=F6DCGio8oZtFJuIldhR&search_mode=AdvancedSearch&update_back2search_link_param=yes) | TS=((dementia)  and  (“cardiac arrhythmia*” or an?emia* or obesity or obese or “prostatic hyperplasia” or “prostatic hypertrophy” or tuberculosis or “chronic hepatitis” or “substance-related disorder?” or “substance abuse” or “drug abuse” or “marijuana abuse” or “cocaine abuse” or “amphetamine abuse” or addiction? or “inflammatory bowel disease?” or “ulcerative colitis” or crohn or ibd or deaf* or blind* or “visual loss” or parkinson* or autis*) )  Indexes=SCI-EXPANDED, SSCI, A&HCI, CPCI-S, CPCI-SSH, ESCI Timespan=All years | [Edit](https://apps.webofknowledge.com/WOS_AdvancedSearch_input.do?product=WOS&SID=F6DCGio8oZtFJuIldhR&search_mode=AdvancedSearch&replaceSetId=19&editState=init) |  |  |
|  | | | | | |
| # 18 | [33,847](https://apps.webofknowledge.com/summary.do?product=WOS&doc=1&qid=19&SID=F6DCGio8oZtFJuIldhR&search_mode=AdvancedSearch&update_back2search_link_param=yes) | TS=((stroke*)  and  (dementia or “cardiac arrhythmia*” or an?emia* or obesity or obese or “prostatic hyperplasia” or “prostatic hypertrophy” or tuberculosis or “chronic hepatitis” or “substance-related disorder?” or “substance abuse” or “drug abuse” or “marijuana abuse” or “cocaine abuse” or “amphetamine abuse” or addiction? or “inflammatory bowel disease?” or “ulcerative colitis” or crohn or ibd or deaf* or blind* or “visual loss” or parkinson* or autis*) )  Indexes=SCI-EXPANDED, SSCI, A&HCI, CPCI-S, CPCI-SSH, ESCI Timespan=All years | [Edit](https://apps.webofknowledge.com/WOS_AdvancedSearch_input.do?product=WOS&SID=F6DCGio8oZtFJuIldhR&search_mode=AdvancedSearch&replaceSetId=18&editState=init) |  |  |
|  | | | | | |
| # 17 | [4,521](https://apps.webofknowledge.com/summary.do?product=WOS&doc=1&qid=18&SID=F6DCGio8oZtFJuIldhR&search_mode=AdvancedSearch&update_back2search_link_param=yes) | TS=((“liver  disease?”  or  “liver  disorder?”)  and  (stroke* or dementia or “cardiac arrhythmia*” or an?emia* or obesity or obese or “prostatic hyperplasia” or “prostatic hypertrophy” or tuberculosis or “chronic hepatitis” or “substance-related disorder?” or “substance abuse” or “drug abuse” or “marijuana abuse” or “cocaine abuse” or “amphetamine abuse” or addiction? or “inflammatory bowel disease?” or “ulcerative colitis” or crohn or ibd or deaf* or blind* or “visual loss” or parkinson* or autis*) )  Indexes=SCI-EXPANDED, SSCI, A&HCI, CPCI-S, CPCI-SSH, ESCI Timespan=All years | [Edit](https://apps.webofknowledge.com/WOS_AdvancedSearch_input.do?product=WOS&SID=F6DCGio8oZtFJuIldhR&search_mode=AdvancedSearch&replaceSetId=17&editState=init) |  |  |
|  | | | | | |
| # 16 | [66,285](https://apps.webofknowledge.com/summary.do?product=WOS&doc=1&qid=17&SID=F6DCGio8oZtFJuIldhR&search_mode=AdvancedSearch&update_back2search_link_param=yes) | TS=((hiv  or  “acquired  immun*  deficiency  syndrome?”  or  aids)  and  (“liver disease?” or “liver disorder?” or stroke* or dementia or “cardiac arrhythmia*” or an?emia* or obesity or obese or “prostatic hyperplasia” or “prostatic hypertrophy” or tuberculosis or “chronic hepatitis” or “substance-related disorder?” or “substance abuse” or “drug abuse” or “marijuana abuse” or “cocaine abuse” or “amphetamine abuse” or addiction? or “inflammatory bowel disease?” or “ulcerative colitis” or crohn or ibd or deaf* or blind* or “visual loss” or parkinson* or autis*) )  Indexes=SCI-EXPANDED, SSCI, A&HCI, CPCI-S, CPCI-SSH, ESCI Timespan=All years | [Edit](https://apps.webofknowledge.com/WOS_AdvancedSearch_input.do?product=WOS&SID=F6DCGio8oZtFJuIldhR&search_mode=AdvancedSearch&replaceSetId=16&editState=init) |  |  |
|  | | | | | |
| # 15 | [26,044](https://apps.webofknowledge.com/summary.do?product=WOS&doc=1&qid=16&SID=F6DCGio8oZtFJuIldhR&search_mode=AdvancedSearch&update_back2search_link_param=yes) | TS=((epilep*  or  seizure?)  and  (hiv or “acquired immun* deficiency syndrome?” or aids or “liver disease?” or “liver disorder?” or stroke* or dementia or “cardiac arrhythmia*” or an?emia* or obesity or obese or “prostatic hyperplasia” or “prostatic hypertrophy” or tuberculosis or “chronic hepatitis” or “substance-related disorder?” or “substance abuse” or “drug abuse” or “marijuana abuse” or “cocaine abuse” or “amphetamine abuse” or addiction? or “inflammatory bowel disease?” or “ulcerative colitis” or crohn or ibd or deaf* or blind* or “visual loss” or parkinson* or autis*) )  Indexes=SCI-EXPANDED, SSCI, A&HCI, CPCI-S, CPCI-SSH, ESCI Timespan=All years | [Edit](https://apps.webofknowledge.com/WOS_AdvancedSearch_input.do?product=WOS&SID=F6DCGio8oZtFJuIldhR&search_mode=AdvancedSearch&replaceSetId=15&editState=init) |  |  |
|  | | | | | |
| # 14 | [948](https://apps.webofknowledge.com/summary.do?product=WOS&doc=1&qid=15&SID=F6DCGio8oZtFJuIldhR&search_mode=AdvancedSearch&update_back2search_link_param=yes) | TS=((“kidney  disease?”  or  “kidney  disorder?”)  and  (epilep* or seizure? or hiv or “acquired immun* deficiency syndrome?” or aids or “liver disease?” or “liver disorder?” or stroke* or dementia or “cardiac arrhythmia*” or an?emia* or obesity or obese or “prostatic hyperplasia” or “prostatic hypertrophy” or tuberculosis or “chronic hepatitis” or “substance-related disorder?” or “substance abuse” or “drug abuse” or “marijuana abuse” or “cocaine abuse” or “amphetamine abuse” or addiction? or “inflammatory bowel disease?” or “ulcerative colitis” or crohn or ibd or deaf* or blind* or “visual loss” or parkinson* or autis*) )  Indexes=SCI-EXPANDED, SSCI, A&HCI, CPCI-S, CPCI-SSH, ESCI Timespan=All years | [Edit](https://apps.webofknowledge.com/WOS_AdvancedSearch_input.do?product=WOS&SID=F6DCGio8oZtFJuIldhR&search_mode=AdvancedSearch&replaceSetId=14&editState=init) |  |  |
|  | | | | | |
| # 13 | [33,586](https://apps.webofknowledge.com/summary.do?product=WOS&doc=1&qid=14&SID=F6DCGio8oZtFJuIldhR&search_mode=AdvancedSearch&update_back2search_link_param=yes) | TS=((“joint  disease*”  or  “rheumatoid  arthritis”  or  osteoarthritis)  and  (“kidney disease?” or “kidney disorder?” or epilep* or seizure? or hiv or “acquired immun* deficiency syndrome?” or aids or “liver disease?” or “liver disorder?” or stroke* or dementia or “cardiac arrhythmia*” or an?emia* or obesity or obese or “prostatic hyperplasia” or “prostatic hypertrophy” or tuberculosis or “chronic hepatitis” or “substance-related disorder?” or “substance abuse” or “drug abuse” or “marijuana abuse” or “cocaine abuse” or “amphetamine abuse” or addiction? or “inflammatory bowel disease?” or “ulcerative colitis” or crohn or ibd or deaf* or blind* or “visual loss” or parkinson* or autis*) )  Indexes=SCI-EXPANDED, SSCI, A&HCI, CPCI-S, CPCI-SSH, ESCI Timespan=All years | [Edit](https://apps.webofknowledge.com/WOS_AdvancedSearch_input.do?product=WOS&SID=F6DCGio8oZtFJuIldhR&search_mode=AdvancedSearch&replaceSetId=13&editState=init) |  |  |
|  | | | | | |
| # 12 | [5,601](https://apps.webofknowledge.com/summary.do?product=WOS&doc=1&qid=13&SID=F6DCGio8oZtFJuIldhR&search_mode=AdvancedSearch&update_back2search_link_param=yes) | TS=((“thyroid  disease?”  or  “thyroid  disorder?”  or  hyperthyroid*  or  hypothyroid*)  and  (“joint disease*” or “rheumatoid arthritis” or osteoarthritis or “kidney disease?” or “kidney disorder?” or epilep* or seizure? or hiv or “acquired immun* deficiency syndrome?” or aids or “liver disease?” or “liver disorder?” or stroke* or dementia or “cardiac arrhythmia*” or an?emia* or obesity or obese or “prostatic hyperplasia” or “prostatic hypertrophy” or tuberculosis or “chronic hepatitis” or “substance-related disorder?” or “substance abuse” or “drug abuse” or “marijuana abuse” or “cocaine abuse” or “amphetamine abuse” or addiction? or “inflammatory bowel disease?” or “ulcerative colitis” or crohn or ibd or deaf* or blind* or “visual loss” or parkinson* or autis*) )  Indexes=SCI-EXPANDED, SSCI, A&HCI, CPCI-S, CPCI-SSH, ESCI Timespan=All years | [Edit](https://apps.webofknowledge.com/WOS_AdvancedSearch_input.do?product=WOS&SID=F6DCGio8oZtFJuIldhR&search_mode=AdvancedSearch&replaceSetId=12&editState=init) |  |  |
|  | | | | | |
| # 11 | [15,178](https://apps.webofknowledge.com/summary.do?product=WOS&doc=1&qid=12&SID=F6DCGio8oZtFJuIldhR&search_mode=AdvancedSearch&update_back2search_link_param=yes) | TS=((dyslipid?emia*  or  hyperlipid?emia*  or  hypercholesterolemia*  or  hypertriglyceridemia*)  and  (“thyroid disease?” or “thyroid disorder?” or hyperthyroid* or hypothyroid* or “joint disease*” or “rheumatoid arthritis” or osteoarthritis or “kidney disease?” or “kidney disorder?” or epilep* or seizure? or hiv or “acquired immun* deficiency syndrome?” or aids or “liver disease?” or “liver disorder?” or stroke* or dementia or “cardiac arrhythmia*” or an?emia* or obesity or obese or “prostatic hyperplasia” or “prostatic hypertrophy” or tuberculosis or “chronic hepatitis” or “substance-related disorder?” or “substance abuse” or “drug abuse” or “marijuana abuse” or “cocaine abuse” or “amphetamine abuse” or addiction? or “inflammatory bowel disease?” or “ulcerative colitis” or crohn or ibd or deaf* or blind* or “visual loss” or parkinson* or autis*) )  Indexes=SCI-EXPANDED, SSCI, A&HCI, CPCI-S, CPCI-SSH, ESCI Timespan=All years | [Edit](https://apps.webofknowledge.com/WOS_AdvancedSearch_input.do?product=WOS&SID=F6DCGio8oZtFJuIldhR&search_mode=AdvancedSearch&replaceSetId=11&editState=init) |  |  |
|  | | | | | |
| # 10 | [16,057](https://apps.webofknowledge.com/summary.do?product=WOS&doc=1&qid=11&SID=F6DCGio8oZtFJuIldhR&search_mode=AdvancedSearch&update_back2search_link_param=yes) | TS=((osteoporosis)  and  (dyslipid?emia* or hyperlipid?emia* or hypercholesterolemia* or hypertriglyceridemia* or “thyroid disease?” or “thyroid disorder?” or hyperthyroid* or hypothyroid* or “joint disease*” or “rheumatoid arthritis” or osteoarthritis or “kidney disease?” or “kidney disorder?” or epilep* or seizure? or hiv or “acquired immun* deficiency syndrome?” or aids or “liver disease?” or “liver disorder?” or stroke* or dementia or “cardiac arrhythmia*” or an?emia* or obesity or obese or “prostatic hyperplasia” or “prostatic hypertrophy” or tuberculosis or “chronic hepatitis” or “substance-related disorder?” or “substance abuse” or “drug abuse” or “marijuana abuse” or “cocaine abuse” or “amphetamine abuse” or addiction? or “inflammatory bowel disease?” or “ulcerative colitis” or crohn or ibd or deaf* or blind* or “visual loss” or parkinson* or autis*) )  Indexes=SCI-EXPANDED, SSCI, A&HCI, CPCI-S, CPCI-SSH, ESCI Timespan=All years | [Edit](https://apps.webofknowledge.com/WOS_AdvancedSearch_input.do?product=WOS&SID=F6DCGio8oZtFJuIldhR&search_mode=AdvancedSearch&replaceSetId=10&editState=init) |  |  |
|  | | | | | |
| # 9 | [22,639](https://apps.webofknowledge.com/summary.do?product=WOS&doc=1&qid=10&SID=F6DCGio8oZtFJuIldhR&search_mode=AdvancedSearch&update_back2search_link_param=yes) | TS=((((mental  or  anxiety  or  mood  or  psychological)  NEAR/1  (disease? or disorder?) )  or  “sleep  disorder”  or  depression?)  and  (osteoporosis or dyslipid?emia* or hyperlipid?emia* or hypercholesterolemia* or hypertriglyceridemia* or “thyroid disease?” or “thyroid disorder?” or hyperthyroid* or hypothyroid* or “joint disease*” or “rheumatoid arthritis” or osteoarthritis or “kidney disease?” or “kidney disorder?” or epilep* or seizure? or hiv or “acquired immun* deficiency syndrome?” or aids or “liver disease?” or “liver disorder?” or stroke* or dementia or “cardiac arrhythmia*” or an?emia* or obesity or obese or “prostatic hyperplasia” or “prostatic hypertrophy” or tuberculosis or “chronic hepatitis” or “substance-related disorder?” or “substance abuse” or “drug abuse” or “marijuana abuse” or “cocaine abuse” or “amphetamine abuse” or addiction? or “inflammatory bowel disease?” or “ulcerative colitis” or crohn or ibd or deaf* or blind* or “visual loss” or parkinson* or autis*) )  Indexes=SCI-EXPANDED, SSCI, A&HCI, CPCI-S, CPCI-SSH, ESCI Timespan=All years | [Edit](https://apps.webofknowledge.com/WOS_AdvancedSearch_input.do?product=WOS&SID=F6DCGio8oZtFJuIldhR&search_mode=AdvancedSearch&replaceSetId=9&editState=init) |  |  |
|  | | | | | |
| # 8 | [22,960](https://apps.webofknowledge.com/summary.do?product=WOS&doc=1&qid=9&SID=F6DCGio8oZtFJuIldhR&search_mode=AdvancedSearch&update_back2search_link_param=yes) | TS=((asthma*)  AND  (((mental or anxiety or mood or psychological)  NEAR/1  (disease? or disorder?) )  or  “sleep  disorder”  or  depression?  or  osteoporosis  or  dyslipid?emia*  or  hyperlipid?emia*  or  hypercholesterolemia*  or  hypertriglyceridemia*  or  “thyroid  disease?”  or  “thyroid  disorder?”  or  hyperthyroid*  or  hypothyroid*  or  “joint  disease*”  or  “rheumatoid  arthritis”  or  osteoarthritis  or  “kidney  disease?”  or  “kidney  disorder?”  or  epilep*  or  seizure?  or  hiv  or  “acquired  immun*  deficiency  syndrome?”  or  aids  or  “liver  disease?”  or  “liver  disorder?”  or  stroke*  or  dementia  or  “cardiac  arrhythmia*”  or  an?emia*  or  obesity  or  obese  or  “prostatic  hyperplasia”  or  “prostatic  hypertrophy”  or  tuberculosis  or  “chronic  hepatitis”  or  “substance-related  disorder?”  or  “substance  abuse”  or  “drug  abuse”  or  “marijuana  abuse”  or  “cocaine  abuse”  or  “amphetamine  abuse”  or  addiction?  or  “inflammatory  bowel  disease?”  or  “ulcerative  colitis”  or  crohn  or  ibd  or  deaf*  or  blind*  or  “visual  loss”  or  parkinson*  or  autis*))  Indexes=SCI-EXPANDED, SSCI, A&HCI, CPCI-S, CPCI-SSH, ESCI Timespan=All years | [Edit](https://apps.webofknowledge.com/WOS_AdvancedSearch_input.do?product=WOS&SID=F6DCGio8oZtFJuIldhR&search_mode=AdvancedSearch&replaceSetId=8&editState=init) |  |  |
|  | | | | | |
| # 7 | [40,415](https://apps.webofknowledge.com/summary.do?product=WOS&doc=1&qid=8&SID=F6DCGio8oZtFJuIldhR&search_mode=AdvancedSearch&update_back2search_link_param=yes) | TS=((“heart  failure”  or  “cardiac  failure”  or  “myocardial  failure”)  and  (asthma* or ((mental or anxiety or mood or psychological)  NEAR/1  (disease? or disorder?) )  or  “sleep  disorder”  or  depression?  or  osteoporosis  or  dyslipid?emia*  or  hyperlipid?emia*  or  hypercholesterolemia*  or  hypertriglyceridemia*  or  “thyroid  disease?”  or  “thyroid  disorder?”  or  hyperthyroid*  or  hypothyroid*  or  “joint  disease*”  or  “rheumatoid  arthritis”  or  osteoarthritis  or  “kidney  disease?”  or  “kidney  disorder?”  or  epilep*  or  seizure?  or  hiv  or  “acquired  immun*  deficiency  syndrome?”  or  aids  or  “liver  disease?”  or  “liver  disorder?”  or  stroke*  or  dementia  or  “cardiac  arrhythmia*”  or  an?emia*  or  obesity  or  obese  or  “prostatic  hyperplasia”  or  “prostatic  hypertrophy”  or  tuberculosis  or  “chronic  hepatitis”  or  “substance-related  disorder?”  or  “substance  abuse”  or  “drug  abuse”  or  “marijuana  abuse”  or  “cocaine  abuse”  or  “amphetamine  abuse”  or  addiction?  or  “inflammatory  bowel  disease?”  or  “ulcerative  colitis”  or  crohn  or  ibd  or  deaf*  or  blind*  or  “visual  loss”  or  parkinson*  or  autis*))  Indexes=SCI-EXPANDED, SSCI, A&HCI, CPCI-S, CPCI-SSH, ESCI Timespan=All years | [Edit](https://apps.webofknowledge.com/WOS_AdvancedSearch_input.do?product=WOS&SID=F6DCGio8oZtFJuIldhR&search_mode=AdvancedSearch&replaceSetId=7&editState=init) |  |  |
|  | | | | | |
| # 6 | [91,092](https://apps.webofknowledge.com/summary.do?product=WOS&doc=1&qid=7&SID=F6DCGio8oZtFJuIldhR&search_mode=AdvancedSearch&update_back2search_link_param=yes) | TS=((“myocardial  infarct*”  or  “cardiovascular  strok*”)  and  (“heart failure” or “cardiac failure” or “myocardial failure” or asthma* or ((mental or anxiety or mood or psychological)  NEAR/1  (disease? or disorder?) )  or  “sleep  disorder”  or  depression?  or  osteoporosis  or  dyslipid?emia*  or  hyperlipid?emia*  or  hypercholesterolemia*  or  hypertriglyceridemia*  or  “thyroid  disease?”  or  “thyroid  disorder?”  or  hyperthyroid*  or  hypothyroid*  or  “joint  disease*”  or  “rheumatoid  arthritis”  or  osteoarthritis  or  “kidney  disease?”  or  “kidney  disorder?”  or  epilep*  or  seizure?  or  hiv  or  “acquired  immun*  deficiency  syndrome?”  or  aids  or  “liver  disease?”  or  “liver  disorder?”  or  stroke*  or  dementia  or  “cardiac  arrhythmia*”  or  an?emia*  or  obesity  or  obese  or  “prostatic  hyperplasia”  or  “prostatic  hypertrophy”  or  tuberculosis  or  “chronic  hepatitis”  or  “substance-related  disorder?”  or  “substance  abuse”  or  “drug  abuse”  or  “marijuana  abuse”  or  “cocaine  abuse”  or  “amphetamine  abuse”  or  addiction?  or  “inflammatory  bowel  disease?”  or  “ulcerative  colitis”  or  crohn  or  ibd  or  deaf*  or  blind*  or  “visual  loss”  or  parkinson*  or  autis*))  Indexes=SCI-EXPANDED, SSCI, A&HCI, CPCI-S, CPCI-SSH, ESCI Timespan=All years | [Edit](https://apps.webofknowledge.com/WOS_AdvancedSearch_input.do?product=WOS&SID=F6DCGio8oZtFJuIldhR&search_mode=AdvancedSearch&replaceSetId=6&editState=init) |  |  |
|  | | | | | |
| # 5 | [47,179](https://apps.webofknowledge.com/summary.do?product=WOS&doc=1&qid=6&SID=F6DCGio8oZtFJuIldhR&search_mode=AdvancedSearch&update_back2search_link_param=yes) | TS=(((cardiac  or  cardiovascular  or  coronary)  NEAR/1  (disease? or disorder? or failure) )  AND  (“myocardial infarct*” or “cardiovascular strok*” or “heart failure” or “cardiac failure” or “myocardial failure” or asthma* or ((mental or anxiety or mood or psychological)  NEAR/1  (disease? or disorder?) )  or  “sleep  disorder”  or  depression?  or  osteoporosis  or  dyslipid?emia*  or  hyperlipid?emia*  or  hypercholesterolemia*  or  hypertriglyceridemia*  or  “thyroid  disease?”  or  “thyroid  disorder?”  or  hyperthyroid*  or  hypothyroid*  or  “joint  disease*”  or  “rheumatoid  arthritis”  or  osteoarthritis  or  “kidney  disease?”  or  “kidney  disorder?”  or  epilep*  or  seizure?  or  hiv  or  “acquired  immun*  deficiency  syndrome?”  or  aids  or  “liver  disease?”  or  “liver  disorder?”  or  stroke*  or  dementia  or  “cardiac  arrhythmia*”  or  an?emia*  or  obesity  or  obese  or  “prostatic  hyperplasia”  or  “prostatic  hypertrophy”  or  tuberculosis  or  “chronic  hepatitis”  or  “substance-related  disorder?”  or  “substance  abuse”  or  “drug  abuse”  or  “marijuana  abuse”  or  “cocaine  abuse”  or  “amphetamine  abuse”  or  addiction?  or  “inflammatory  bowel  disease?”  or  “ulcerative  colitis”  or  crohn  or  ibd  or  deaf*  or  blind*  or  “visual  loss”  or  parkinson*  or  autis*))  Indexes=SCI-EXPANDED, SSCI, A&HCI, CPCI-S, CPCI-SSH, ESCI Timespan=All years | [Edit](https://apps.webofknowledge.com/WOS_AdvancedSearch_input.do?product=WOS&SID=F6DCGio8oZtFJuIldhR&search_mode=AdvancedSearch&replaceSetId=5&editState=init) |  |  |
|  | | | | | |
| # 4 | [31,059](https://apps.webofknowledge.com/summary.do?product=WOS&doc=1&qid=5&SID=F6DCGio8oZtFJuIldhR&search_mode=AdvancedSearch&update_back2search_link_param=yes) | TS=((neoplasm?  or  cancer?)  AND  (((cardiac or cardiovascular or coronary)  NEAR/1  (disease? or disorder? or failure) )  or  “myocardial  infarct*”  or  “cardiovascular  strok*”  or  “heart  failure”  or  “cardiac  failure”  or  “myocardial  failure”  or  asthma*  or  ((mental or anxiety or mood or psychological)  NEAR/1  (disease? or disorder?) )  or  “sleep  disorder”  or  depression?  or  osteoporosis  or  dyslipid?emia*  or  hyperlipid?emia*  or  hypercholesterolemia*  or  hypertriglyceridemia*  or  “thyroid  disease?”  or  “thyroid  disorder?”  or  hyperthyroid*  or  hypothyroid*  or  “joint  disease*”  or  “rheumatoid  arthritis”  or  osteoarthritis  or  “kidney  disease?”  or  “kidney  disorder?”  or  epilep*  or  seizure?  or  hiv  or  “acquired  immun*  deficiency  syndrome?”  or  aids  or  “liver  disease?”  or  “liver  disorder?”  or  stroke*  or  dementia  or  “cardiac  arrhythmia*”  or  an?emia*  or  obesity  or  obese  or  “prostatic  hyperplasia”  or  “prostatic  hypertrophy”  or  tuberculosis  or  “chronic  hepatitis”  or  “substance-related  disorder?”  or  “substance  abuse”  or  “drug  abuse”  or  “marijuana  abuse”  or  “cocaine  abuse”  or  “amphetamine  abuse”  or  addiction?  or  “inflammatory  bowel  disease?”  or  “ulcerative  colitis”  or  crohn  or  ibd  or  deaf*  or  blind*  or  “visual  loss”  or  parkinson*  or  autis*))  Indexes=SCI-EXPANDED, SSCI, A&HCI, CPCI-S, CPCI-SSH, ESCI Timespan=All years | [Edit](https://apps.webofknowledge.com/WOS_AdvancedSearch_input.do?product=WOS&SID=F6DCGio8oZtFJuIldhR&search_mode=AdvancedSearch&replaceSetId=4&editState=init) |  |  |
|  | | | | | |
| # 3 | [162,988](https://apps.webofknowledge.com/summary.do?product=WOS&doc=1&qid=4&SID=F6DCGio8oZtFJuIldhR&search_mode=AdvancedSearch&update_back2search_link_param=yes) | TS=((hypertens*  or  "high  blood  pressure*")  AND  (neoplasm? or cancer? or ((cardiac or cardiovascular or coronary)  NEAR/1  (disease? or disorder? or failure) )  or  “myocardial  infarct*”  or  “cardiovascular  strok*”  or  “heart  failure”  or  “cardiac  failure”  or  “myocardial  failure”  or  asthma*  or  ((mental or anxiety or mood or psychological)  NEAR/1  (disease? or disorder?) )  or  “sleep  disorder”  or  depression?  or  osteoporosis  or  dyslipid?emia*  or  hyperlipid?emia*  or  hypercholesterolemia*  or  hypertriglyceridemia*  or  “thyroid  disease?”  or  “thyroid  disorder?”  or  hyperthyroid*  or  hypothyroid*  or  “joint  disease*”  or  “rheumatoid  arthritis”  or  osteoarthritis  or  “kidney  disease?”  or  “kidney  disorder?”  or  epilep*  or  seizure?  or  hiv  or  “acquired  immun*  deficiency  syndrome?”  or  aids  or  “liver  disease?”  or  “liver  disorder?”  or  stroke*  or  dementia  or  “cardiac  arrhythmia*”  or  an?emia*  or  obesity  or  obese  or  “prostatic  hyperplasia”  or  “prostatic  hypertrophy”  or  tuberculosis  or  “chronic  hepatitis”  or  “substance-related  disorder?”  or  “substance  abuse”  or  “drug  abuse”  or  “marijuana  abuse”  or  “cocaine  abuse”  or  “amphetamine  abuse”  or  addiction?  or  “inflammatory  bowel  disease?”  or  “ulcerative  colitis”  or  crohn  or  ibd  or  deaf*  or  blind*  or  “visual  loss”  or  parkinson*  or  autis*))  Indexes=SCI-EXPANDED, SSCI, A&HCI, CPCI-S, CPCI-SSH, ESCI Timespan=All years | [Edit](https://apps.webofknowledge.com/WOS_AdvancedSearch_input.do?product=WOS&SID=F6DCGio8oZtFJuIldhR&search_mode=AdvancedSearch&replaceSetId=3&editState=init) |  |  |
|  | | | | | |
| # 2 | [265,318](https://apps.webofknowledge.com/summary.do?product=WOS&doc=1&qid=3&SID=F6DCGio8oZtFJuIldhR&search_mode=AdvancedSearch&update_back2search_link_param=yes) | TS=((diabet*)  AND  (hypertens* or "high blood pressure*" or neoplasm? or cancer? or ((cardiac or cardiovascular or coronary)  NEAR/1  (disease? or disorder? or failure) )  or  “myocardial  infarct*”  or  “cardiovascular  strok*”  or  “heart  failure”  or  “cardiac  failure”  or  “myocardial  failure”  or  asthma*  or  ((mental or anxiety or mood or psychological)  NEAR/1  (disease? or disorder?) )  or  “sleep  disorder”  or  depression?  or  osteoporosis  or  dyslipid?emia*  or  hyperlipid?emia*  or  hypercholesterolemia*  or  hypertriglyceridemia*  or  “thyroid  disease?”  or  “thyroid  disorder?”  or  hyperthyroid*  or  hypothyroid*  or  “joint  disease*”  or  “rheumatoid  arthritis”  or  osteoarthritis  or  “kidney  disease?”  or  “kidney  disorder?”  or  epilep*  or  seizure?  or  hiv  or  “acquired  immun*  deficiency  syndrome?”  or  aids  or  “liver  disease?”  or  “liver  disorder?”  or  stroke*  or  dementia  or  “cardiac  arrhythmia*”  or  an?emia*  or  obesity  or  obese  or  “prostatic  hyperplasia”  or  “prostatic  hypertrophy”  or  tuberculosis  or  “chronic  hepatitis”  or  “substance-related  disorder?”  or  “substance  abuse”  or  “drug  abuse”  or  “marijuana  abuse”  or  “cocaine  abuse”  or  “amphetamine  abuse”  or  addiction?  or  “inflammatory  bowel  disease?”  or  “ulcerative  colitis”  or  crohn  or  ibd  or  deaf*  or  blind*  or  “visual  loss”  or  parkinson*  or  autis*))  Indexes=SCI-EXPANDED, SSCI, A&HCI, CPCI-S, CPCI-SSH, ESCI Timespan=All years | [Edit](https://apps.webofknowledge.com/WOS_AdvancedSearch_input.do?product=WOS&SID=F6DCGio8oZtFJuIldhR&search_mode=AdvancedSearch&replaceSetId=2&editState=init) |  |  |
|  | | | | | |
| # 1 | [30,826](https://apps.webofknowledge.com/summary.do?product=WOS&doc=1&qid=2&SID=F6DCGio8oZtFJuIldhR&search_mode=AdvancedSearch&update_back2search_link_param=yes) | TS=(("chronic  obstructive  pulmonary  disease"  or  copd  or  "pulmonary  disease?"  or  "pulmonary  disorder?")  and  (diabet* or hypertens* or "high blood pressure*" or neoplasm? or cancer? or ((cardiac or cardiovascular or coronary)  NEAR/1  (disease? or disorder? or failure) )  or  "myocardial  infarct*"  or  "cardiovascular  strok*"  or  "heart  failure"  or  "cardiac  failure"  or  "myocardial  failure"  or  asthma*  or  ((mental or anxiety or mood or psychological)  NEAR/1  (disease? or disorder?) )  or  "sleep  disorder"  or  depression?  or  osteoporosis  or  dyslipid?emia*  or  hyperlipid?emia*  or  hypercholesterolemia*  or  hypertriglyceridemia*  or  "thyroid  disease?"  or  "thyroid  disorder?"  or  hyperthyroid*  or  hypothyroid*  or  "joint  disease*"  or  "rheumatoid  arthritis"  or  osteoarthritis  or  "kidney  disease?"  or  "kidney  disorder?"  or  epilep*  or  seizure?  or  hiv  or  "acquired  immun*  deficiency  syndrome?"  or  aids  or  "liver  disease?"  or  "liver  disorder?"  or  stroke*  or  dementia  or  "cardiac  arrhythmia*"  or  an?emia*  or  obesity  or  obese  or  "prostatic  hyperplasia"  or  "prostatic  hypertrophy"  or  tuberculosis  or  "chronic  hepatitis"  or  "substance-related  disorder?"  or  "substance  abuse"  or  "drug  abuse"  or  "marijuana  abuse"  or  "cocaine  abuse"  or  "amphetamine  abuse"  or  addiction?  or  "inflammatory  bowel  disease?"  or  "ulcerative  colitis"  or  crohn  or  ibd  or  deaf*  or  blind*  or  "visual  loss"  or  parkinson*  or  autis*))  Indexes=SCI-EXPANDED, SSCI, A&HCI, CPCI-S, CPCI-SSH, ESCI Timespan=All years | [Edit](https://apps.webofknowledge.com/WOS_AdvancedSearch_input.do?product=WOS&SID=F6DCGio8oZtFJuIldhR&search_mode=AdvancedSearch&replaceSetId=1&editState=init) |  |  |
