# Supplementary material for: Costs of multimorbidity: a systematic review and meta-analyses
Source: BMC Med. 2022 Jul 19;20:234. doi: 10.1186/s12916-022-02427-9 (PMC9295506; doi:10.1186/s12916-022-02427-9)
Supplement: Supplementary file 2 — Additional file 2. Screening checklists [file 12916_2022_2427_MOESM2_ESM.docx]

**Additional file 3: Checklists**

**Phase one: Title and abstract screening and first classification**

This checklist will be used for the first screening of articles after deduplication. For those articles where the question cannot be answered by reading the title, the full abstract will be read.

All retained articles will be classified as “Retained phase 1”. All classified articles will be compared between the two independent researchers.

| **Q** | **Question** | **Answer** | **Action following answer** | **Comments /examples** |
| --- | --- | --- | --- | --- |
| **1** | **Does the article talk about cost of multimorbidity?** | Yes | Go to Q2 |  |
|  |  | No | Classify as “No cost” | If clear that the study does not concern cost of MM. |
|  |  | Maybe | Go to Q2 |  |
| **2** | **Does the article talk about resource utilization related to multimorbidity?** | Yes | Go to Q3 |  |
|  |  | No | Classify as “No RU” and go to Q3 | If clear that the study does not concern resource utilization related to MM. |
|  |  | Maybe | Go to Q3 |  |
| **3** | **Does the article talk about any kind of predictors of high costs related to multimorbidity?** | Yes | Go to Q4 |  |
|  |  | No | Classify as “No predictors” and go to Q4 | If clear that the study does not concern predictors of high costs related to multimorbidity. |
|  |  | Maybe | Go to Q4 |  |
| **4** | **Is the study design appropriate?** | Yes | Classify as “Retained phase 1” | RCT, cohort study, case control study, cross sectional study |
|  |  | No | Classify as “Not quantitative” | If only qualitative data or commentary. |
|  |  | Maybe | Classify as “Retained phase 1” | In case of a Review, classify as “Review”, this can be used for screening of references and discussion. |

**Phase two: Full article screening and second classification**

For all articles classified a “Retained Phase 1” the full article will be read and the articles will be further classified as below. For those articles classified by one of the researchers as “Maybe”, discussion between the researchers and if necessary, communication with the author will be used to decide on final inclusion. In case of discrepancies between researchers on classification and inclusion, discussion between the researchers and a third researchers will be used to reach a final decision.

| **Q** | **Question** | **Answer** | **Action following answer** | **Inclusion/ Exclusion criteria** |
| --- | --- | --- | --- | --- |
| **1** | **Is the full text of the article available?** | Yes | Go to Q2 |  |
|  |  | No | Classify as “No full text” |  |
|  |  | Maybe | Classify as “To be checked for full text” |  |
| **2** | **Is the full text article available in English?** | Yes | Go to Q3 |  |
|  |  | No | Classify as “Not English” |  |
|  |  | Maybe | Classify as “To be checked for English availability” |  |
| **3** | **Does the study specify the specific combination(s) of chronic conditions** | Yes | Go to Q4 | E.g. diabetes and hypertension |
|  |  | No | Classify as “No specified MM”, and “Retained phase 2” | Studies that do not specify the specific combination(s) of chronic conditions may be considered to be retained for research question 1. |
|  |  | Maybe | Classify as “Maybe” |  |
| **4** | **Does the study specify total costs (not incremental costs)?** | Yes | Classify as “Retained phase 2” |  |
|  |  | No | Classify as “No total cost”, and “Retained phase 2” | Studies that do not specify the total costs may be considered to be retained for research question 1. |
|  |  | Maybe | Classify as “Maybe” |  |
