# Supplementary material for: Costs of multimorbidity: a systematic review and meta-analyses
Source: BMC Med. 2022 Jul 19;20:234. doi: 10.1186/s12916-022-02427-9 (PMC9295506; doi:10.1186/s12916-022-02427-9)
Supplement: Supplementary file 4 — Additional file 4. Definition of terminologies [file 12916_2022_2427_MOESM4_ESM.docx]

Additional file 1: Definition of terminologies

1. ***Multimorbidity***: co-existence of two or more chronic conditions
2. ***Chronic conditions***: conditions that last for a period of one year or longer, which demands continuous care and/or hinder daily activities and functioning
3. ***Costs of multimorbidity***: encompasses direct and indirect costs associated with the prevention, diagnosis, treatment and management of multimorbidity, regardless of the payer
4. ***Direct cost***: all costs incurred that are attributable to the use of healthcare resource, intervention or service. Direct costs can be further categorized into direct medical costs and direct non-medical costs
5. ***Direct medical cost***: the cost of a defined health service or intervention and all follow-up costs for medication and medical equipment; e.g. Diagnostics, hospitalization, outpatient, emergency, drugs and medicine, equipment.
6. ***Direct non-medical cost***: costs incurred in the process of seeking and after receiving health services, that are not involved in the direct purchasing of medical products or services; e.g. Transportation/travel costs, food, accommodation and additional paid caregiver time.
7. ***Indirect cost***: costs incurred as a result of losses from the disease(s) or disease management; e.g. Time loss, wage loss, interest from debst/loans.
